# A quadratically regularized functional canonical correlation analysis for identifying the global structure of pleiotropy with NGS data

**DOI:** 10.1371/journal.pcbi.1005788

**Published:** 2017-10-17

**Authors:** Nan Lin, Yun Zhu, Ruzong Fan, Momiao Xiong

**Affiliations:** 1 Department of Biostatistics and Data Science, School of Public Health, The University of Texas Health Science Center at Houston, Houston, TX, United States of America; 2 Department of Epidemiology, Tulane University School of Public Health and Tropical Medicine, New Orleans, LA, United States of America; 3 Biostatistics and Bioinformatics Branch (BBB), Division of Intramural Population Health Research (DIPHR), *Eunice Kennedy Shriver* National Institute of Child Health and Human Development, National Institutes of Health (NIH), Bethesda, MD, United States of America; University of Minnesota, UNITED STATES

## Abstract

Investigating the pleiotropic effects of genetic variants can increase statistical power, provide important information to achieve deep understanding of the complex genetic structures of disease, and offer powerful tools for designing effective treatments with fewer side effects. However, the current multiple phenotype association analysis paradigm lacks breadth (number of phenotypes and genetic variants jointly analyzed at the same time) and depth (hierarchical structure of phenotype and genotypes). A key issue for high dimensional pleiotropic analysis is to effectively extract informative internal representation and features from high dimensional genotype and phenotype data. To explore correlation information of genetic variants, effectively reduce data dimensions, and overcome critical barriers in advancing the development of novel statistical methods and computational algorithms for genetic pleiotropic analysis, we proposed a new statistic method referred to as a quadratically regularized functional CCA (QRFCCA) for association analysis which combines three approaches: (1) quadratically regularized matrix factorization, (2) functional data analysis and (3) canonical correlation analysis (CCA). Large-scale simulations show that the QRFCCA has a much higher power than that of the ten competing statistics while retaining the appropriate type 1 errors. To further evaluate performance, the QRFCCA and ten other statistics are applied to the whole genome sequencing dataset from the TwinsUK study. We identify a total of 79 genes with rare variants and 67 genes with common variants significantly associated with the 46 traits using QRFCCA. The results show that the QRFCCA substantially outperforms the ten other statistics.

## Author summary

Association analysis of multiple phenotypes will unravel the genetic pleiotropic structures of multiple phenotypes, provide a powerful tool for developing drug with fewer side effects. To increase the power of the tests for high dimensional association analysis of multiple phenotypes with next-generation sequencing data, a key issue is to develop novel statistics that can effectively extract informative internal representation and features from high dimensional data. However, the current paradigm of association analysis of multiple phenotypes does not efficiently utilize the rich correlation structure of the genotype and phenotype data. To shift the paradigm of association analysis from shallow multivariate analysis to comprehensive functional analysis, we proposed a new general statistical framework referred to as a quadratically regularized functional canonical correlation analysis (QRFCCA) for association test which explores rich correlation information in the genotype and phenotype data. Large-scale simulations demonstrate that the QRFCCA has a much higher power than that of the many existing statistics while retaining the appropriate type 1 errors. To further evaluate the new approach, the QRFCCA are also applied to the TwinsUK study with 46 traits and sequencing data. The results show that the QRFCCA substantially outperforms the other statistics.

## Introduction

As of February 6th, 2017, a catalog of published Genome-Wide Association Studies (GWAS) had reported significant association of 26,791 SNPs with more than 1704 traits in 2,337 publications [1]. It is reported that more than 4.6% of the SNPs and 16.9% of the genes were significantly associated with more than one trait [2]. These results demonstrate that genetic pleiotropic effects, which refers to the effects of a genetic variant affecting multiple traits, play a crucial role in uncovering genetic structures of correlated phenotypes [3–10]. Most genetic analyses of quantitative traits have focused on a single trait association analysis, analyzing each phenotype independently [11]. Less attention has been paid to comprehensive analysis of pleiotropic effects [12]. However, multiple phenotypes are correlated due to shared genetic and environmental effects [13]. The integrative analysis of correlated phenotypes which tests the association of a genetic variant with multiple traits often increases the statistical power to identify genetic associations and increases the precision of genetic effect estimation [13–16]. It is increasingly recognized that the genetic effect can be detected only when the association of the genetic variant with the multiple traits are jointly tested [17]. It is also noted that directional pleiotropy indicating that the genetic effects of the variant on the multiple traits are in the same direction (all positive or all negative) widely exists [18]. Changes of one trait may cause undesired changes of other traits. Investigation of pleiotropy provides a tool for designing the effective treatment with fewer side effects.

Two types of approaches can be used for genetic pleiotropic analysis. One approach is to utilize summary statistics for estimating genetic correlations and testing association of genetic variants with multiple traits [17–22]. An alternative approach is to use individual genotypic information for association analysis of multiple correlated traits [23]. The focus of this paper is to use individual genotypes for pleiotropic analysis. Three major types of methods are commonly used to explore the association of genetic variants with multiple correlated phenotypes: multivariate techniques including multivariate linear models [15,24–33], linear mixed models [11,16,33,34] and functional linear models [35], the combinations of univariate association measures for different phenotypes [36–39, 43], and dimension reduction methods including principal component analysis (PCA) [14,40–43], and canonical correlation analysis [30,44–47].

Statistical methods for testing the association of common variants with multiple traits have been well developed and successfully applied [2]. The methods for pleiotropic analysis of rare variants are still under development [25, 48]. Next-generation sequencing and modern biosensing techniques have generated dozens of millions of SNPs and large numbers of clinical and intermediate phenotypes. The current multiple phenotype association analysis paradigm lacks breadth (the number of phenotypes and genetic variants jointly analyzed at a time) and depth (hierarchical structure of phenotype and genotypes). Most approaches perform analysis on the subsets of the full data space that are often missing, but now available. A key issue for high dimensional pleiotropic analysis is to effectively extract rich correlation information from extremely high dimensional genotypic and phenotypic data. The statistical power of the methods that do not efficiently explore dimension reduction of both phenotype and genotype data will be limited. Despite their wide applications to the pleiotropic analysis, the current pleiotropic analysis methods share the same drawbacks. These methods, particularly multivariate analysis methods, either do not use data dimension reduction or ignore the rich linkage disequilibrium structure of genomic data when data dimension reduction is used. The most widely used methods for pleiotropic analysis are originally designed for analyzing a small number of phenotypes and common variant data. Due to the lack of efficient analytic platforms, the current pleiotropic analysis methods have not been applied to large-scale real genetic pleiotropic analysis with a large number of phenotypes and next-generation sequencing (NGS) data. To overcome these limitations and fully take the advantages of the rich linkage disequilibrium information across a genomic region, we combine two approaches: (1) functional data analysis and (2) quadratically regularized CCA to develop a novel statistical method that is referred to as a quadratically regularized functional canonical correlation analysis (QRFCCA) for testing the association of genomic regions with multiple traits. The QRFCCA first transforms the high dimensional correlated discrete genotype data across the genes or genomic regions to a few regularized functional principal components in the low orthonormal eigenfunctional space by functional principal component analysis (FPCA). Then, the QRFCCA will further utilize the quadratically recognized matrix factorization to project both the phenotype data and compressed genomic data by FPCA to low dimensional space with much fewer number of bases (components) than the traditional matrix factorization or PCA and changed distribution of eigenvalues in which the proportion of top eigenvalues substantially increases. The QRFCCA dramatically reduces the dimensions of both genotype and phenotype data while fully retaining the original genotypic and phenotypic information.

To evaluate the performance of the developed QRFCCA for association analysis of multiple phenotypes, we conduct large-scale simulations comparing QRFCCA to ten statistics: Sparse CCA (SCCA) [49], MSKAT [50], GAMuT [25], FCCA [51], kernel CCA (KCCA) [52], CCA, A Unified Score-Based Association Test (USAT) [53], PCA (applying to both phenotypes and genotypes), MANOVA (multivariate ANOVA applied to multiple phenotypes and SNPs), and minP (minimum of P-values for testing the association of single SNP with multiple phenotypes) and demonstrate that the QRFCCA has a much higher power than other competing statistics while retaining the correct type 1 error rates. Finally, the QRFCCA and ten other statistics are applied to the whole genome sequencing dataset from the TwinsUK study where 756 individuals with 33,746 genes and 46 traits in 13 major phenotype groups are included in the analysis. We find that the QRFCCA for pleiotropic analysis substantially outperforms the ten other statistics. A program for implementing the developed QRFCCA for association analysis of multiple phenotypes can be downloaded from our website https://sph.uth.edu/research/centers/hgc/xiong/software.htm and https://cran.r-project.org/web/packages/.

The UK10K data can be downloaded from http://www.uk10k.org.

## Materials and methods

The essential component of the QRFCCA is data dimension reduction. To efficiently reduce the data dimension while exploring the rich correlation information in the data, the data dimension reduction pipelines of proposed QRFCCA for pleiotropic genetic analysis consist of four steps: (1) FPCA, (2) matrix factorization, (3) quadratic regularization, and (4) CCA. The FPCA changes the raw data (genotype data only or both genotype and phenotype data when the phenotypes are function-valued, for example, RNA-seq data) to the functional principal component (FPC) representation that can capture the correlation features. The matrix factorization is to embed the FPC scores or multiple scale phenotypes into the low dimensional vector space. It compresses the FPC score or phenotype data to a few new features that are another level of representation of data. Quadratic regularization further compresses the data and changes the representation of FPC scores or phenotypes. Finally, CCA is used as an effective tool for two-view dimension reduction. QRFCCA combines dimension reduction in different levels of data representations.

Genotype-phenotype association attempts to unravel the relationships between certain combinations of genetic variants from multiple loci and certain combinations of multiple phenotypes. The CCA measures the linear relationships between two multidimensional sets of variables and hence will be naturally used as a general framework for identifying the association between genotypes and phenotypes.

### Smooth functional principal component analysis

We first briefly introduce the smooth functional principal component analysis (FPCA) for genetic variant data [54]. We first review the definition of genetic variant profiles. Let *t* be the position of a genetic variant within a genomic region and *T* be the length of the genomic region being considered. For convenience, we rescale the region from [0,*T*] to [0,1]. We can view *t* as a continuous variable in the interval [0,1] because the density of genetic variants is high. We define the genotype function of the *i*-th individual as

$$x_{i}(t)=\{\begin{array}{c} 2\;\mathrm{MM}\\ 1\;\mathrm{Mm}\\ 0\;\mathrm{mm} \end{array},\;i=1,\cdots ,n$$ (1)

where M is an allele at the genomic position *t* and *n* is the number of sampled individuals. Although the FPCA can also be applied to function-valued phenotypes, for example, RNA-seq data, this paper will focus on genotype function, assuming that the phenotypes are scale variables.

A key step of the FPCA is that the functional data are projected into a finite-dimensional space of FPCs or eigenfunction [51, 54]. Let *β*_*j*_(*t*),*j* = 1,2,… be a set of FPCs which can be obtained from solving integral eigenequation (details are referred to the book [51] or paper [54]). Similar to Fourier series or wavelet expansions, the genotype profile function *x*_*i*_(*t*) can be expanded in terms of orthogonal FPCs where FPCs were taken as basis functions:

$$x_{i}(t)=\sum_{j=1}^{J}\xi_{ij}\beta_{j}(t),$$ (2)

where *ξ*_*ij*_ is the FPC score of the *i*^*th*^ individual which can be estimated by

$$\xi_{ij}=\int_{T}x_{i}(t)\beta_{j}(t)dt,$$ (3)

where integral was calculated numerically [51]. FPCs were constructed from genotype functions for each gene and contained linkage disequilibrium information. Each individual has a number of FPC scores. The FPC scores can represent the original genotype functions. FPCs can efficiently compress the data. For example, in the TwinsUK data set, 2,633,479 common SNPs and 2,249,090 rare SNPs and 33,746 genes were included in the FPCA analysis. The FPCA analysis was performed for each gene. On average, 2.5 FPCs each gene can account for 90% of common variant variation and 2.7 FPCs each gene can account for 90% of rare variant variation.

### Matrix factorization

Consider a data matrix *A* ∈ *R*^*n*×*q*^ consisting of *n* samples with *q* features (variables). The data matrix *A* represents the genotype data, the FPC scores or the phenotype data. The phenotype data include both continuous and discrete values. The *i*^*th*^ row of *A* is a vector of *q* features for the *i*^*th*^ sample, and the *j*^*th*^ column of *A* is a vector of the *j*^*th*^ feature across the set of *n* samples. Matrix factorization is used as a general framework to embed the genetic and phenotype data into the low dimensional vector space to reduce the data dimension and remove anomalous or noise data points [55]. To accomplish this, we first seek the best rank-*l* approximation to the matrix *A* by factorizing it into a product of two low rank matrices.

Let *G* ∈ *R*^*n*×*l*^ and *H* ∈ *R*^*l*×*q*^. Assume that the rank of *A* is *r*. Therefore, *r* ≤ min(*n*,*q*). Matrix factorization attempts to minimize the approximation error:

$$\underset{G,H}{\min}\;\|A-GH{\|}_{F}^{2},$$ (4)

where ‖.‖_*F*_ denotes the Frobenius norm of a matrix.

A solution to problem (4) can be found by truncating the singular value decomposition (SVD) of *A*_*ij*_ [55]. Let the SVD of *A* be given by

$$A=U\Lambda V^{T},$$ (5)

where *U* = [*u*_1_,…,*u*_*r*_] ∈ *R*^*n*×*r*^, *V* = [*v*_1_,…,*v*_*r*_] ∈ *R*^*q*×*r*^, *U*^*T*^*U* = *I*_*r*×*r*_, *V*^*T*^*V* = *I*_*r*×*r*_, and Λ = *diag*(*λ*_1_,…,*λ*_*r*_) ∈ *R*^*r*×*r*^ with *λ*_1_ ≥ *λ*_2_ ≥ … ≥ *λ*_*r*_ > 0. The columns of *U* and *V* are referred to as the left and right singular vectors of *A*, respectively, and *λ*_1_,…, *λ*_*r*_ are referred to as the singular values of *A*.

Let Λ_*l*_ = *diag*(*λ*_1_,…,*λ*_*l*_), *U*_*l*_ = [*u*_1_,…,*u*_*l*_] and *V*_*l*_ = [*v*_1_,…,*v*_*l*_]. Define $G=U_{l}\Lambda_{l}^{1/2}$ and $H=\Lambda_{l}^{1/2}V_{l}$. The best rank-*l* approximation to the matrix *A* or the *l*-rank matrix factorization of *A* is then given by [55]

A≈GH. (6)

The matrix factorization compresses the *q* features (variables) in the original data set to *l* < *q* new features and hence reduces the data dimension.

### Canonical correlation analysis

An alternative to multivariate linear regression analysis, the CCA is a popular analytic platform for genetic pleiotropic analysis. The goal of CCA is to seek optimal correlation between linear combinations of two sets of variables: the set of traits and the set of SNPs. The CCA measures the strength of association between the multiple SNPs and the traits. The pairs of linear combinations are called canonical variates and their correlations are called canonical correlations [56].

Consider a phenotype matrix *Y* = [*Y*_1_,…,*Y*_*k*_] with *k* traits and genotype matrix *X* = [*X*_1_,…,*X*_*p*_] with *p* SNPs or FPC scores. We assume that *p* + *k* variables *Z* = [*X*^*T*^,*Y*^*T*^]^*T*^ jointly have the covariance matrix

$$\Sigma_{zz}=\left[\begin{array}{cc} \Sigma_{xx} & \Sigma_{xy}\\ \Sigma_{yx} & \Sigma_{yy} \end{array}\right].$$

Let

$$R^{2}=\Sigma_{yy}^{-1/2}\Sigma_{yx}\Sigma_{xx}^{-1}\Sigma_{xy}\Sigma_{yy}^{-1/2}$$ (7)

and

$$K=\Sigma_{xx}^{-1/2}\Sigma_{xy}\Sigma_{yy}^{-1/2}.$$

The SVD of *K* is

$$K=U\Lambda V^{T},$$ (8)

where Λ = *diag*(*λ*_1_,…,*λ*_*q*_) and *q* = min(*p*,*k*) is the smaller number of variables in the two genotype-phenotype datasets.

It is well known that the canonical vectors are

$$\begin{array}{l} A=\Sigma_{xx}^{-1/2}U,\\ B=\Sigma_{yy}^{-1/2}V, \end{array}$$ (9)

and the vector of canonical correlations are

$$CC={\left[\lambda_{1},\ldots ,\lambda_{q}\right]}^{T}.$$ (10)

A squared canonical correlation measures the proportion of variance linearly shared by the two sets of canonical variates derived from the input genotype-phenotype data sets.

Canonical correlations between the genotype and phenotypes measure the strength of their association. The CCA produces multiple canonical correlations. But we wish to use a single number to measure the association of the genetic variation with the multiple traits. We propose to use the summation of the square of the singular values as a measure to quantify the association of the genetic variation within a gene or genomic region with the multiple traits:

$$r=\sum_{i=1}^{q}\lambda_{i}^{2}=\mathrm{Tr}(\Lambda^{2})=\mathrm{Tr}(R^{2}).$$ (11)

To test the association of the genetic variation in a gene or genomic region is equivalent to test independence between the two genotype-phenotype datasets *X* and *Y* or to test the hypothesis that each variable in the set *X* is uncorrelated with each variable in the set *Y*. The null hypothesis of no association of the genotype data *X* with the phenotype dataset *Y* can be formulated as

$$H_{0}:\Sigma_{xy}=0.$$

The likelihood ratio for testing *H*_0_: ∑_*xy*_ = 0 is

$$\Lambda_{r}=\frac{\left|\Sigma_{zz}\right|}{\left|\Sigma_{xx}||\Sigma_{yy}\right|}=\prod_{i=1}^{q}(1-\lambda_{i}^{2}),$$ (12)

which is equal to the Wilks’ lambda Λ defined in the multivariate linear regression model.

This demonstrates that testing for association using multivariate linear regression can be treated as special case of CCA [57].

We usually define the likelihood ratio test statistic for testing the association as:

$$T_{CCA}=-N\sum_{i=1}^{q}\log(1-\lambda_{i}^{2}).$$ (13)

For small $\lambda_{i}^{2}$, *T*_*CCA*_ can be approximated by $N\sum_{i=1}^{q}\lambda_{i}^{2}=Nr$, where *r* is the measure of association of the genetic variation in the gene or genomic region with the multiple traits. The stronger the association, the higher the power that the test statistic can test the association.

Under the null hypothesis *H*_0_: ∑_*xy*_ = 0, *T*_*CCA*_ is asymptotically distributed as a central $\chi_{pk}^{2}$. When sample size is large, Bartlett (1939) suggests using the following statistic for hypothesis testing:

$$T_{CCA}=-[N-\frac{\left(q+3\right)}{2}]\sum_{i=1}^{q}\log(1-\lambda_{i}^{2}).$$ (14)

### Quadratically regularized matrix factorization and canonical correlation analysis

The power of the test statistics in CCA depends on the squared canonical correlations or eigenvalues of the matrix *R*^2^. We wish to increase the power via changing distribution of the canonical correlations and data reduction. In matrix factorization, for fixed rank *l*, we want to approximate the matrix *A* by the product of two factor matrices *G* and *H* as accurately as possible. However, the Frobenius norm of the matrices *G* and *H* may be large. We need to balance the approximation accuracy and the Frobenius norm of the factor matrices. Specifically, we add the Frobenius norm of the factor matrices to the objective in Eq (4). The optimization problem (4) is now transformed to the quadratically regularized matrix factorization problem:

$$\underset{G,H}{\min}\;F=\|A-GH{\|}_{F}^{2}+\mu \|G{\|}_{F}^{2}+\mu \|H{\|}_{F}^{2}.$$ (15)

From Eq (5), the matrices *G* and *H* have the forms:

$$G=U_{l}\Lambda_{l}^{1/2}\;\mathrm{and}\;H=\Lambda_{l}^{1/2}V_{l},$$ (16)

where Λ_*l*_ = *diag*(*τ*_1_,…,*τ*_*l*_,0,…,0). The matrices *G* and *H* are determined by *τ*_*j*_. Seeking the matrices *G* and *H* to optimize the objective function in Eq (15) is equivalent to finding solutions *τ*_*j*_ to minimize the objective function in Eq (15).

Using techniques in (55), we can show that the solution to optimization problem (15) is

$$\tau_{j}={\left(\lambda_{j}-\mu \right)}_{+},$$ (17)

where (*a*)_+_ = max(*a*,0) and *λ*_*j*_ is defined in Eq (8).

In practice, a singular value is selected as a penalty parameter *μ* such that the sum from the selected singular value to the smallest singular value accounted for 20% of total singular values.

Define the matrix Λ_*l*_ as

$$\Lambda_{l}=diag({\left(\lambda_{1}-\mu \right)}_{+},\ldots ,{\left(\lambda_{l}-\mu \right)}_{+}).$$

Then, factor matrices $G=U_{l}\Lambda_{l}^{1/2}$ and $H=\Lambda_{l}^{1/2}V_{l}$ are the solution to the minimization problem (15). We use truncation of the SVD to keep only the top *l* singular values and soft-thresholding on the singular values to change distribution of the singular values. When *μ* increases beyond some singular values *λ*_*m*_, *l* − *m* + 1 singular values of *GH* will disappear. Analytically, we can easily show that

$$\frac{\lambda_{1}-\mu }{\lambda_{1}-\mu +\lambda_{2}-\mu +\ldots +\lambda_{l}-\mu }>\frac{\lambda_{1}}{\lambda_{1}+\lambda_{2}+\ldots +\lambda_{l}}.$$

In other words, increasing *μ* will move up the proportion of the first singular value in the total of singular values.

The phenotype data consist of 756 samples with 46 traits. The initial largest singular value and total singular values of the phenotype data are 73.97 and 1030.14 respectively. S1 Fig shows that the proportion of the first singular value in the total of singular values is an increasing function of threshold *μ*. This clearly demonstrates that adding quadratic regularization results in changing the distribution of the singular values of the factor matrices. Therefore, we can expect that regularized matrix factorization for data reduction will increase the power to detect association of the genetic variation with the traits.

Quadratically regularized matrix factorization and CCA have broad applications. There are a number of ways to use the quadratically regularized matrix factorization for data reduction in the association analysis which are briefly summarized as follows.

1. Continuous phenotypes and NGS genotype data (dimension reduced genotype data using FPCA-gene-based association study).
  We can first apply the quadratically regularized matric factorization to both multiple phenotype data and FPC score data in a gene or genomic region and then use CCA for association analysis of multiple traits. This analysis will be referred to as quadratically regularized FCCA (QRFCCA) for multiple trait association analysis. If only a single trait is considered, the quadratically regularized matrix factorization is only applied to the FPC score in the gene.
2. Continuous phenotypes and multiple SNPs.
  Quadratically regularized matrix factorization is first applied to both multiple phenotypes and SNPs for data reduction and then CCA is used for multiple trait association analysis. This procedure is referred to as QRMCCA.
3. Continuous phenotypes and a single SNP.
  Quadratically regularized matrix factorization is first applied to multiple phenotypes and then CCA is used for multiple trait association analysis. This procedure is referred to as QRSCCA.
4. Both functional phenotype such as RNA-seq data and functional genotype data such as NGS data. FPCA is first applied to both functional phenotype and genotype data to obtain FPC scores for both phenotypes and genotypes. Quadratically regularized matrix factorization is applied to FPC scores of both functional phenotype and genotype data. Finally, CCA is used for multiple trait association analysis. This procedure is referred to as QRBFCCA.

Quadratically recognized matrix factorization can also be applied to the *K* or *R*^2^ matrix in the CCA. The test statistics then use the singular values of the reduced *K* or *R*^2^ matrix to test association of genetic variation with a trait.

To adjust for covariates, we first regress phenotypes on the covariates. If the covariates are the same for all traits, then the multivariate regression will be used to simultaneously regress all the phenotypes to the covariates. Otherwise, we regress each trait to the covariates individually. The residuals are then taken as one set of variables (similar to phenotypes) for CCA.

### Connection between the strength of association and heritability

Next we study the relationships between the association of the genetic variation within a gene or genomic region with the multiple traits and the heritability of quantitative traits. For simplicity, we assume that the genetic variation is the only major contribution to the phenotypic variation and we will not consider the covariates. In Supplemental note A, we show that the narrow heritability is equal to the measure of association *r* of the genetic variation within a gene or genomic region with the multiple traits defined in Eq (11).

### Relationship among CCA, kernel CCA, functional CCA, cross-covariance operator, dependence measure and other association tests

In supplemental note B, we use reproducing kernel Hilbert spaces (RKHS) as a general framework and the covariance operator as a general tool for unifying CCA, kernel CCA, functional CCA and other association analyses including GAMuT. Many multivariate and functional statistical methods such as regression, CCA, kernel regression, kernel CCA, functional regression and functional CCA can be used to test the association of genetic variants with the phenotypes. In supplemental note B, we develop a unified framework for association tests to reveal the relationships among various multivariate and functional association tests.

In Supplemental Note B, we define two kernels *K*_*x*_ = (*K*(*X*_*i*_,*X*_*j*_))_*m*×*m*_, *K*_*y*_ = (*K*(*Y*_*i*_,*Y*_*j*_))_*m*×*m*_, $G=I_{m}-\frac{1}{m}1_{m}$, and centered kernels: ${\tilde{K}}_{x}=GK_{x}G$ and ${\tilde{K}}_{y}=GK_{y}G$. Using the centered kernels we can define the dependence measure as (N39):

$$\frac{1}{m^{2}}\mathrm{Trace}\;({\tilde{K}}_{x}{\tilde{K}}_{y}),$$ (18)

which is the basis for the GAMuT test [25]. In Supplemental note B, we show that the KCCA is quite similar to the kernel independent test and that the association measure in the KCCA is exactly equal to the dependence measure.

Finally, we consider the FCCA. In Supplemental note B, we unify multivariate association tests and functional association tests. Suppose that the FPC scores form a feature space. In supplemental note B, we define the feature maps from the original functional data to the FPC score feature space. We show that the dependence measure in the FPC score-based kernel analysis is asymptotically equal to the association measure of the FCCA. This implies that the FCCA is a specific kernel analysis that uses the FPC score to define the kernels instead of directly using the genotype data to define the kernels.

## Results

### Null distribution of test statistics

To examine the null distribution of test statistics for association analysis of multiple traits, we performed a series of simulation studies to compare their empirical levels with the nominal ones. We calculated the type I error rates for rare alleles, and both rare and common alleles. We first assumed the model for multiple traits:

$$Y_{i}=\mu +\varepsilon_{i},i=1,\ldots ,n,$$

where *Y*_*i*_ = [*y*_*i*1_,…,*y*_*ik*_], *k* is the number of traits, *μ* is a vector of overall means, and *ε*_*i*_ is distributed as *N*(0,Σ), where ∑ is a *k* × *k* residual correlation matrix. We similarly model the correlation matrix as in Broadaway et al [25]. We also consider three scenarios of low residual correlation among phenotypes with pair-wise correlation selected from a uniform (0.1, 0.2) distribution, moderate residual correlation with pair-wise correlation selected from a uniform (0.2, 0.4) distribution, and high residual correlation with pair-wise correlation selected from a uniform (0.4, 0.7) distribution.

We randomly generated 1,000,000 haplotypes with gene *C16orf62* from 659 samples of European origin in The 1000 Genome Project. 1,000 SNPs with 600 rare variants (frequencies ranging from 0.0005 to 0.01) and 400 common variants (frequencies larger than 0.01) were randomly selected from C16orf62 gene. The number of sampled individuals for type 1 error simulations from populations of 500,000 individuals ranged from 500 to 2,000. A total of 10,000 simulations were repeated. The type 1 error rates were estimated as the proportion of the datasets under the null distribution in which the P-values were less than or equal to the significance level.

Tables 1 and 2 summarized the type 1 error rates of the eleven statistics: QRFCCA, Sparse CCA (SCCA) [49], GAMuT [25], MSKAT [50], FCCA, Kernel CCA (KCCA), CCA, A Unified Score-Based Association Test (USAT)[53], PCA (applying to both phenotypes and genotypes), MANOVA (multivariate ANOVA applied to multiple phenotypes and multiple SNS) and minP (minimum of P-values for testing the association of single SNP with multiple phenotypes) for testing the association of rare variants, and both rare and common variants, within a genomic region with 15 high correlated traits, respectively, at the nominal levels α = 0.05, α = 0.01, α = 0.001, *α* = 0.0001, and *α* = 0.00001. Tables S1-S16 showed type 1 error rates of the eleven statistics for testing the association of rare variants, and both rare and common variants with 5, 10 and 15 traits under three scenarios: low, moderate and high correlations. These tables showed that the estimated type 1 error rates of the QRFCCA across a range of assumptions were not appreciably different from the nominal levels *α* = 0.05, *α* = 0.01, *α* = 0.001, *α* = 0.0001, and *α* = 0.00001. We also observed that the type 1 error rates of other ten statistics, in most scenarios, were appropriate.

**Table 1:** Type 1 error rates of 11 statistics for testing the association of rare variants in a gene with 15 high correlated traits.

Sample Size	Nominal Level	QRFCCA	FCCA	GAMuT	SCCA	USAT	MANOVA	CCA	PCA	KCCA	MSKAT	minP
	0.05	0.0492	0.0477	0.0524	0.0513	0.0399	0.0494	0.0473	0.0495	0.0442	0.0484	0.0284
	0.01	0.0099	0.0097	0.0104	0.0101	0.0074	0.0102	0.0097	0.0096	0.0095	0.0095	0.0058
500	0.001	0.0011	0.0011	0.001	0.001	0.0008	0.0009	0.001	0.001	0.001	0.001	0.0007
	0.0001	0.0001	0.0001	0.00009	0.0001	0.00009	0.00009	0.00009	0.00009	0.0001	0.0001	0.0001
	0.00001	0.00001	0.00001	0.00001	0.00001	0.00001	0.00001	0.00001	0.00001	0.00001	0.00001	0.00001
	0.05	0.0497	0.0514	0.0548	0.0477	0.0595	0.0512	0.0464	0.0501	0.0485	0.0484	0.0253
	0.01	0.0099	0.0106	0.0106	0.0099	0.0116	0.0105	0.0107	0.0095	0.0099	0.0107	0.0053
1000	0.001	0.001	0.001	0.0011	0.0011	0.0011	0.001	0.001	0.001	0.0009	0.001	0.0005
	0.0001	0.00009	0.00009	0.00009	0.00008	0.00009	0.00009	0.00009	0.0001	0.00009	0.0001	0.00009
	0.00001	0.00001	0.00001	0.00001	0.00001	0.00001	0.00001	0.00001	0.00001	0.00001	0.00001	0.00001
	0.05	0.0498	0.0461	0.0526	0.0486	0.0598	0.0497	0.0496	0.0533	0.0417	0.0469	0.0271
	0.01	0.0096	0.0104	0.0113	0.0098	0.0123	0.0106	0.0106	0.0099	0.0099	0.01	0.005
2000	0.001	0.0009	0.001	0.0011	0.001	0.0014	0.001	0.0009	0.001	0.0009	0.001	0.0005
	0.0001	0.00011	0.0001	0.0001	0.0001	0.00011	0.0001	0.0001	0.0001	0.00011	0.0001	0.0001
	0.00001	0.00001	0.00001	0.00001	0.00001	0.00001	0.00001	0.00001	0.00001	0.00001	0.00001	0.00001

**Table 2:** Type 1 error rates of 11 statistics for testing the association of common and rare variants in a gene with 15 highly correlated traits.

Sample Size	Nominal Level	QRFCCA	FCCA	GAMuT	SCCA	USAT	MANOVA	CCA	PCA	KCCA	MSKAT	minP
	0.05	0.0518	0.0519	0.0492	0.0516	0.0404	0.0497	0.0473	0.0482	0.049	0.0488	0.0276
	0.01	0.0095	0.0105	0.0103	0.0103	0.0076	0.0094	0.01	0.0097	0.0104	0.0103	0.0057
500	0.001	0.001	0.0009	0.001	0.0009	0.0008	0.001	0.001	0.001	0.0009	0.001	0.0006
	0.0001	0.00011	0.00011	0.00012	0.00012	0.00011	0.0001	0.00011	0.00011	0.0001	0.0001	0.0001
	0.00001	0.00001	0.00001	0.00001	0.00001	0.00001	0.00001	0.00001	0.00001	0.00001	0.00001	0.00001
	0.05	0.0503	0.0467	0.055	0.0522	0.0579	0.0484	0.0513	0.0495	0.0442	0.0491	0.028
	0.01	0.0097	0.0099	0.0105	0.0099	0.0109	0.0095	0.0097	0.0099	0.0096	0.0096	0.0055
1000	0.001	0.001	0.001	0.0011	0.001	0.0012	0.001	0.0011	0.001	0.0009	0.0011	0.0005
	0.0001	0.00013	0.00012	0.00012	0.00013	0.00012	0.0001	0.00012	0.00011	0.00014	0.0001	0.00011
	0.00001	0.00001	0.00001	0.00001	0.00001	0.00001	0.00001	0.00001	0.00001	0.00001	0.00001	0.00001
	0.05	0.05	0.0512	0.0556	0.053	0.0694	0.0517	0.0534	0.0479	0.0481	0.0513	0.0266
	0.01	0.0102	0.0096	0.0113	0.0098	0.0125	0.0099	0.0106	0.0096	0.0096	0.0097	0.0052
2000	0.001	0.001	0.001	0.0011	0.001	0.0012	0.001	0.001	0.001	0.001	0.001	0.0005
	0.0001	0.00013	0.00011	0.00011	0.00012	0.0001	0.0001	0.0001	0.0001	0.00012	0.0001	0.00011
	0.00001	0.00001	0.00001	0.00001	0.00001	0.00001	0.00001	0.00001	0.00001	0.00001	0.00001	0.00001

### Power evaluation

To evaluate the performance of the QRFCCA in association analysis, we used simulated data to estimate power of eleven statistics for testing the association of a gene or a genomic region with the traits. We simulated 5, 10 and 15 traits with low, moderate and high correlations. An additive genetic model was used to summarize all genetic effects of causal variants in the gene or genomic region.

For each individual, 5, 10, 15 quantitative traits were simulated by the summation of genetic effects and the residual correlation between the traits. Let $h_{k}^{2}$ be the narrow heritability of the *k*^*th*^ trait. Assume that each SNP had a 2% chance to be associated with a trait and its genetic effect on the *k*^*th*^ trait was equal to the $\sqrt{\frac{h_{k}^{2}}{MAF}}$ multiplied by the number of minor alleles where *MAF* denoted the frequency of the minor allele. This indicates that the genetic effect of causal variants was inversely proportional to its minor allele frequency.

We did not assume that the gene of interest was associated with all traits. For each of five traits, ten traits and fifteen traits, we consider three scenarios: (1) the gene of interest was truly associated with three of five assessing traits, six of ten assessing traits and eight of fifteen assessing traits (the gene was associated with 53.3% of traits); (2) the gene of interest was truly associated with two of five assessing traits, four of ten assessing traits and six of fifteen assessing traits (the gene was associated with 40% of traits); and (3) the gene of interest was truly associated with one of five assessing traits, two of ten assessing traits and three of fifteen assessing traits (the gene was associated with 20% of traits). We consider two significant levels: *α* = 0.05 and *α* = 0.00001.

The residual correlation was simulated from a multivariate distribution with mean zero and covariance matrix

$$\left[\begin{array}{cccc} 1-h_{1}^{2} & r_{12}\sqrt{\left(1-h_{1}^{2})(1-h_{2}^{2}\right)} & \cdots & r_{1K}\sqrt{\left(1-h_{1}^{2})(1-h_{K}^{2}\right)}\\ r_{12}\sqrt{\left(1-h_{1}^{2})(1-h_{2}^{2}\right)} & 1-h_{2}^{2} & \cdots & r_{2K}\sqrt{\left(1-h_{2}^{2})(1-h_{K}^{2}\right)}\\ \vdots & \vdots & \vdots & \vdots \\ r_{K1}\sqrt{\left(1-h_{1}^{2})(1-h_{K}^{2}\right)} & r_{K2}\sqrt{\left(1-h_{2}^{2})(1-h_{K}^{2}\right)} & \cdots & 1-h_{K}^{2} \end{array}\right],$$

where the correlation between traits *r*_*ij*_ was randomly generated with uniform distribution: low correlation [0.1–0.2], moderate correlation [0.2–0.4] and high correlation [0.4–0.7]. In summary, the genetic model for power evaluation is given by

$$\left[\begin{array}{ccc} y_{1} & \cdots & y_{K} \end{array}\right]=\left[\begin{array}{ccc} x_{1} & \cdots & x_{q} \end{array}\right]\times \left[\left(\begin{array}{ccc} \alpha_{11} & \cdots & \alpha_{K1}\\ \vdots & \ddots & \vdots \\ \alpha_{1q} & \cdots & \alpha_{Kq} \end{array}\right)\circ \left(\begin{array}{ccc} b_{11} & \cdots & b_{K1}\\ \vdots & \ddots & \vdots \\ b_{1q} & \cdots & b_{Kq} \end{array}\right)\right]\times \left(\begin{array}{ccc} t_{1} & \cdots & 0\\ \vdots & \ddots & \vdots \\ 0 & \cdots & t_{K} \end{array}\right)\times \left(\begin{array}{ccc} h_{1}^{2} & \cdots & 0\\ \vdots & \ddots & \vdots \\ 0 & \cdots & h_{K}^{2} \end{array}\right)+\left[\begin{array}{ccc} \varepsilon_{1} & \cdots & \varepsilon_{K} \end{array}\right],$$

where *y*_*i*_ represented phenotypes, *x*_*j*_ was an indicator variable for coding the genotype of the *j*^*th*^ SNP in the gene, taking values 0, 1, 2 to represent the number of minor alleles at the SNP. *α*_*ij*_ denoted the genetic effect that followed a normal distribution with $N(0,\sqrt{\frac{h_{i}^{2}}{MAF_{j}}})$, where $h_{i}^{2}$ denoted the narrow heritability of the *i*^*th*^ trait, *MAF*_*j*_ denoted the frequency of the minor allele at the *j*^*th*^ SNP, *b*_*ij*_ in the matrix represented the probability of the *j*^*th*^ SNP being the causal variant for the *i*^*th*^ trait and followed a binomial distribution *B*(1,0.02). Notation ∘ denoted an element-wise matrices multiplication, *t*_*i*_ ∼ *B*(1,0.6),*t*_*i*_ ∼ *B*(1,0.4),*t*_*i*_ ∼ *B*(1,0.2) represented the probability of the gene being tested contributing the genetic effect to the *i*^*th*^ trait for scenarios 1, 2 and 3, respectively, and $h_{i}^{2}$ denoted the heritability of the *i*^*th*^ trait and followed a uniform distribution *U*(0.005,0.015), *ε*_*i*_, *i* = 1,…,*K* denoted residuals and followed a multivariate normal distribution as defined above.

The genotype data in type 1 error calculations were also used for power evaluation. A total of 10,000 simulations were repeated for the power calculations.

**Fig 1:**
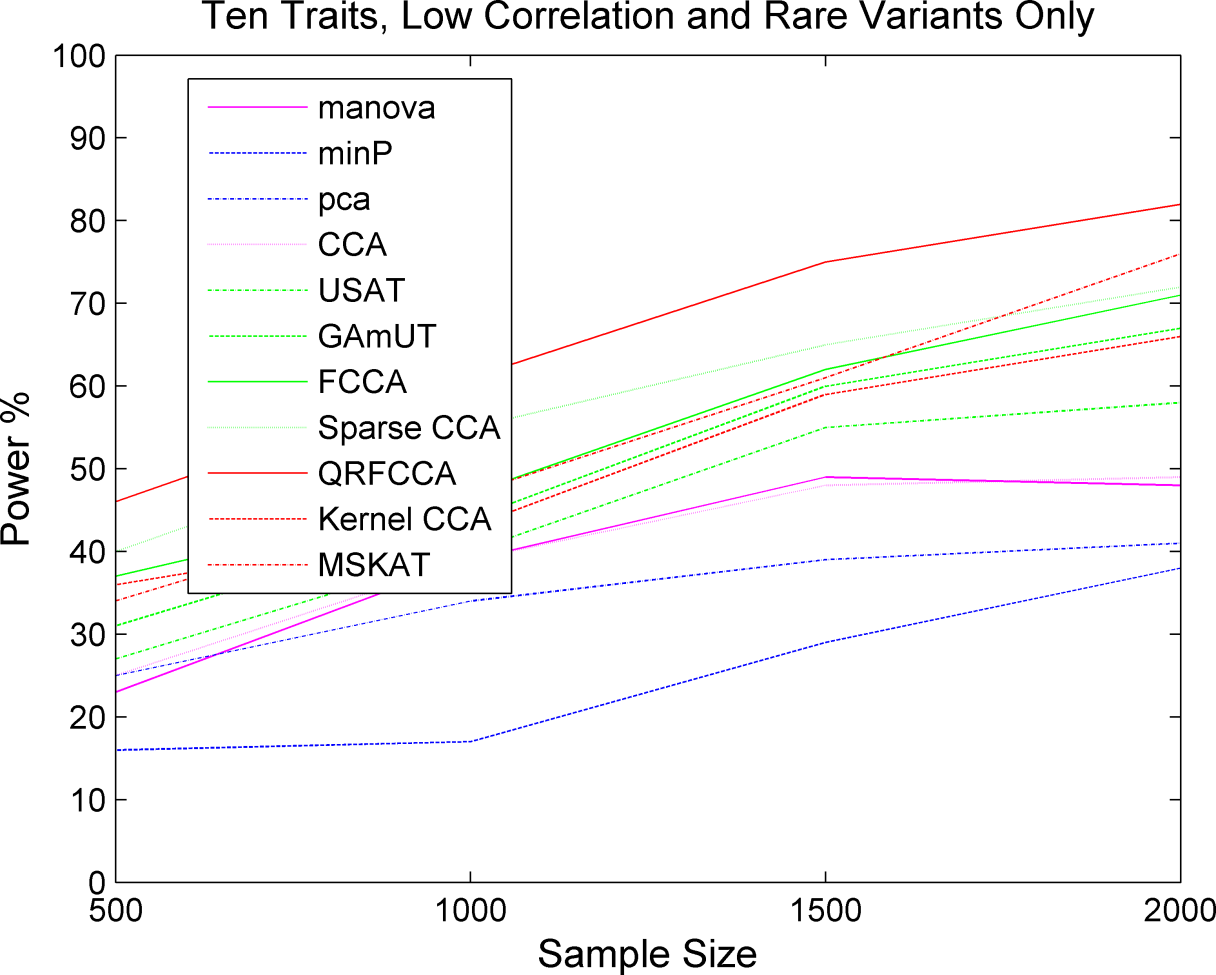
The power of curves as a function of sample sizes of 11 statistics for collectively testing the association of all rare variants in the gene with 10 traits with low correlations for the scenario 1 at the significance level *α* = 0.05.

**Fig 2:**
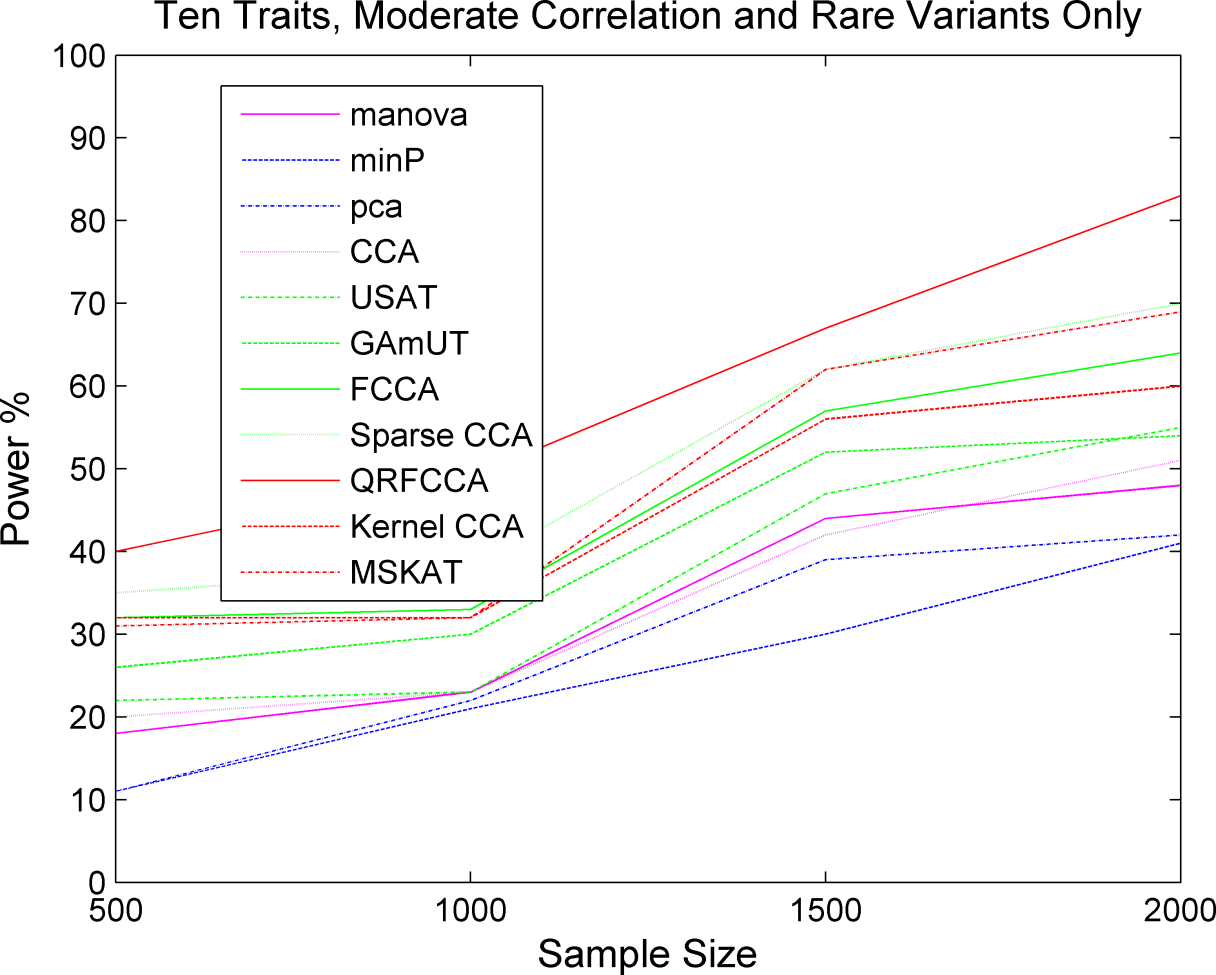
The power of curves as a function of sample sizes of 11 statistics for collectively testing the association of all rare variants in the gene with 10 traits with moderate correlations for the scenario 1 at the significance level *α* = 0.05.

**Fig 3:**
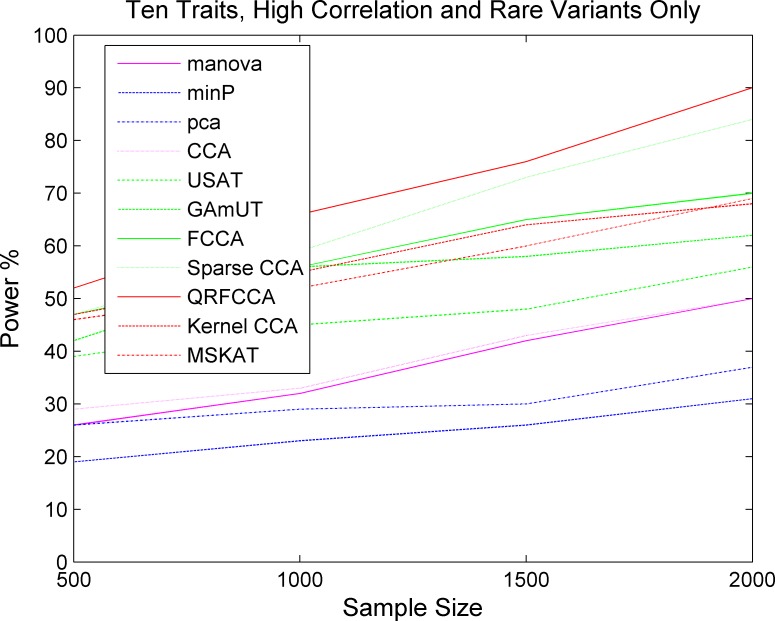
The power of curves as a function of sample sizes of 11 statistics for collectively testing the association of all rare variants in the gene with 10 traits with high correlations for the scenario 1 at the significance level *α* = 0.05.

We first compared the power of QRFCCA with ten other competing statistics that are described in type 1 error rate calculations for testing the association of rare variants with multiple continuous traits. Power was estimated as a function of sample sizes. Figs 1–3 plotted the power of the curves as a function of sample sizes of the eleven statistics for collectively testing the association of all rare variants in the gene with 10 low, moderately and highly correlated traits for scenario 1, respectively, at the significance level α = 0.05. The power curves of eleven statistics for testing the association of the gene including only rare variants with 5 and 15 low, moderately and highly correlated traits for scenario 1, respectively, were plotted in Fig S2–S4, Fig S5–S7. The power of the curves as a function of sample sizes of the eleven statistics for collectively testing the association of all rare variants in the gene with 10 low, moderately and highly correlated traits for scenarios 2 and 3, respectively, were plotted in Fig S8–S13. We observed several remarkable features. First, we clearly observed that the QRFCCA had highest power among all eleven statistics, followed by SCCA for most scenarios considered. Second, in general, the power of the FCCA was higher than that of the KCCA and the GAMuT, but their differences were very small. Third, we often observed that the sparse CCA had higher power than the classical CCA. Fourth, the power of the MSKAT was often higher than that of GAMuT.

**Fig 4:**
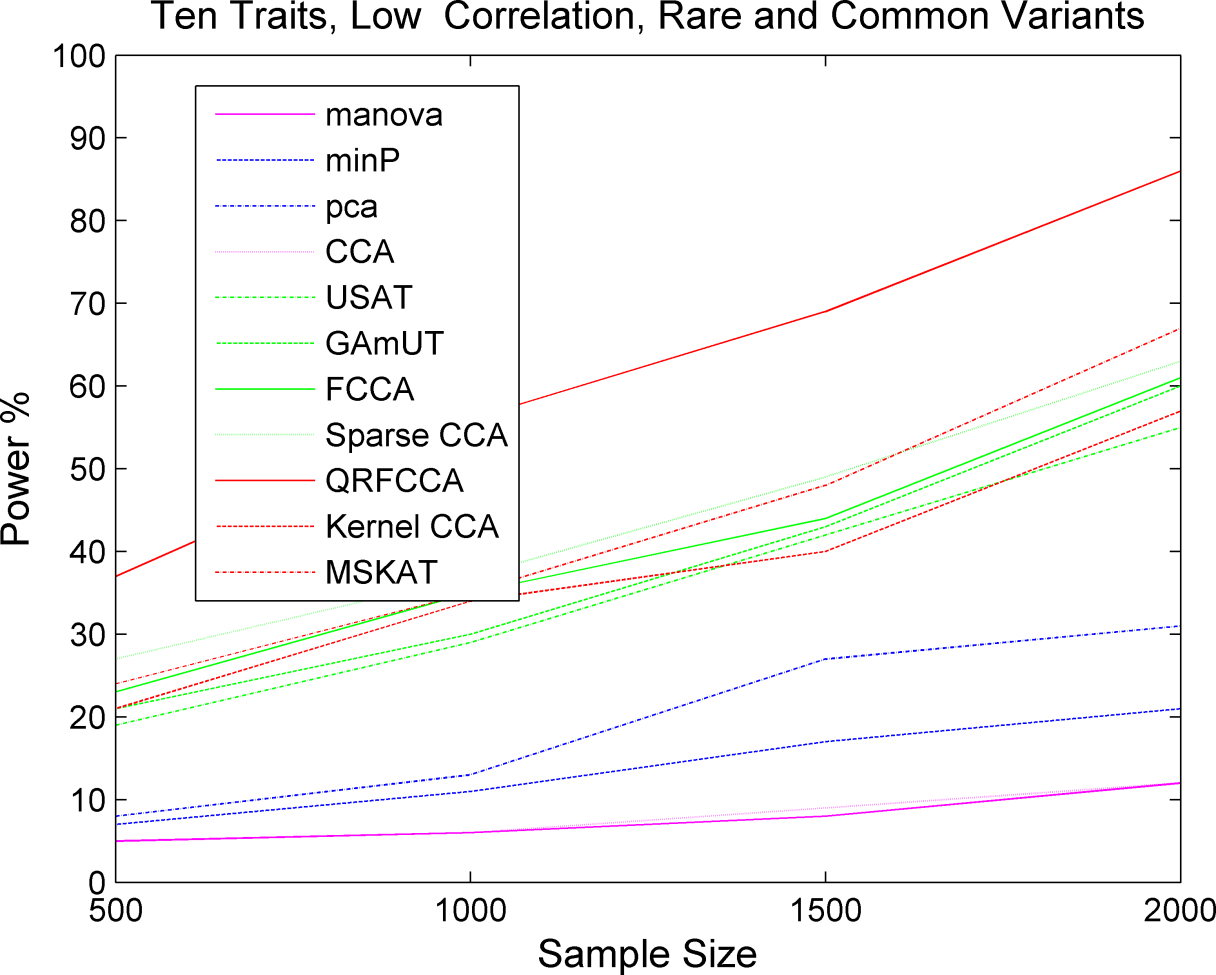
The power of curves as a function of sample sizes of 11 statistics for collectively testing the association of the gene including both rare and common variants with 10 traits with low correlations for the scenario 1 at the significance level *α* = 0.05.

**Fig 5:**
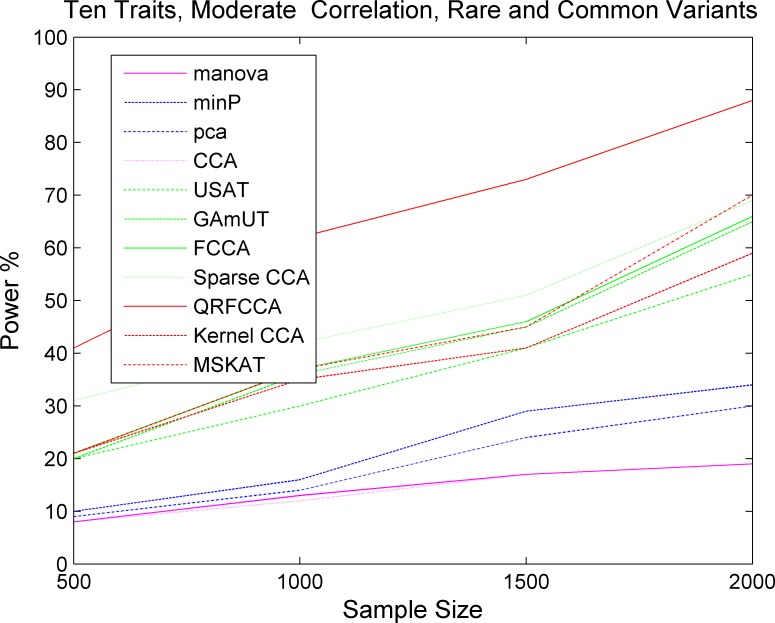
The power of curves as a function of sample sizes of 11 statistics for collectively testing the association of the gene including both rare and common variants with 10 traits with moderate correlations for the scenario 1at the significance level α = 0.05.

**Fig 6:**
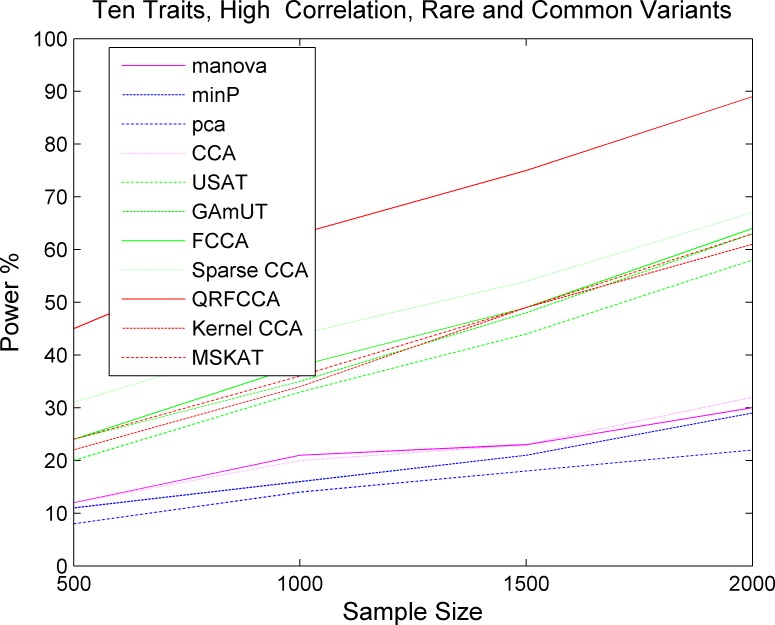
The power of curves as a function of sample sizes of 11 statistics for collectively testing the association of the gene including both rare and common variants with 10 traits with high correlations for the scenario 1 at the significance level *α* = 0.05.

Next we investigated whether the power pattern of the eleven statistics for testing the association of the gene with only rare variants would still hold when testing the association of the gene with both rare and common variants. Figs 4–6 presented the power curves of eleven statistics for testing the association of the gene including both rare and common variants with 10 low, moderately and highly correlated traits for scenario 1 at the significance level α = 0.05, respectively. Fig S14-S16 and Fig S17-S19 showed the power curves of the eleven statistics for testing the association of the gene including both rare and common variants with 5 low, moderately, and highly correlated traits, and 15 low, moderately, and highly correlated traits for scenario 1, respectively. We also presented the power curves of the eleven statistics for testing the association of the gene including both rare and common variants with 10 low, moderately, and highly correlated traits for the scenarios 2 and 3, respectively, in Figure S20-S25. We first observed that the power of the QRFCCA in any cases was much higher than that of all other ten statistics. Then, we observed that differences in power between the QRFCCA and other ten statistics for the common variants were much higher than for the rare variants and their differences increased as the correlation between traits increased or the number of the traits which the gene was associated with increased. We also observed that the power of all statistics for testing the association of common variants was higher than that of all statistics for testing the association of rare variants. Finally we observed the power of the classic CCA, manova, PCA and min P was very low.

To show that the dimension reduction of the QRFCCA for the phenotype will also improve the power of the test, we presented Fig S26 that showed the power of eleven statistics for testing the power of a single common variant with 15 low correlated traits as a function of sample sizes. We observed that the QRFCCA still had higher power than all other ten statistics for testing the association of the single common variant with multiple traits due to its efficient dimension reduction, followed by kernel CCA. Since the MSKAT and GAMuT did not provide tools for efficiently reducing the dimension of the phenotypes, the power of the MSKAT and GAMuT were much smaller than that of the QRFCCA, and even smaller than that of kernel CCA.

Finally, we presented Fig S27-S32 showing the power curves of the eleven statistics for testing the association of the gene including rare variants only, and both rare and common variants with 15 highly correlated traits in scenarios 1, 2 and 3, at the significance level *α* = 0.00001, respectively. Again, we still observed that the power of the QRFCCA was the highest among eleven statistics. However, when the significance level was reduced from *α* = 0.05 to *α* = 0.00001 the power of all statistics was reduced. We also observed that when the significance level was reduced, the simulations were unstable. In this case, we need to increase the number of simulations.

These figures demonstrated that the QRFCCA substantially outperformed the ten other statistics and the difference in power between the QRFCCA and other statistics for the both rare and common variants was much larger than that for the rare variants only. This demonstrated that the regularization in singular vectors plays a more important role in association analysis of both rare and common variants than that in association analysis of only rare variants.

### Application to real data examples

Investigation of the contribution of the entire allelic spectrum of genetic variation to multiple traits are still at its infancy. The systematic searching for both common and rare variants associated with large number of traits is essential for unraveling the genetic architecture of complex diseases. To further evaluate the performance, the QRFCCA and ten other statistics were applied to the UK-10K dataset. The UK-10K Cohorts project used a low read depth whole-genome sequencing (WGS) to assess the contribution of the genetic variants to the sixty-four different traits [58]. However, missing phenotypes were found in many individuals. To ensure no missing phenotypes in individuals, we included 765 individuals with 2,240,049 SNPs in 33,746 genes, and shared 46 traits in 13 major phenotypic groups which covered a wide range of traits (Table S17) in the analysis. We took the rank-based inverse normal transformation of the phenotypes [59] as trait values. Principal components (PCs) can be used for covariates to adjust for the impact of population structure.

### Association analysis of rare variants

**Fig 7:**
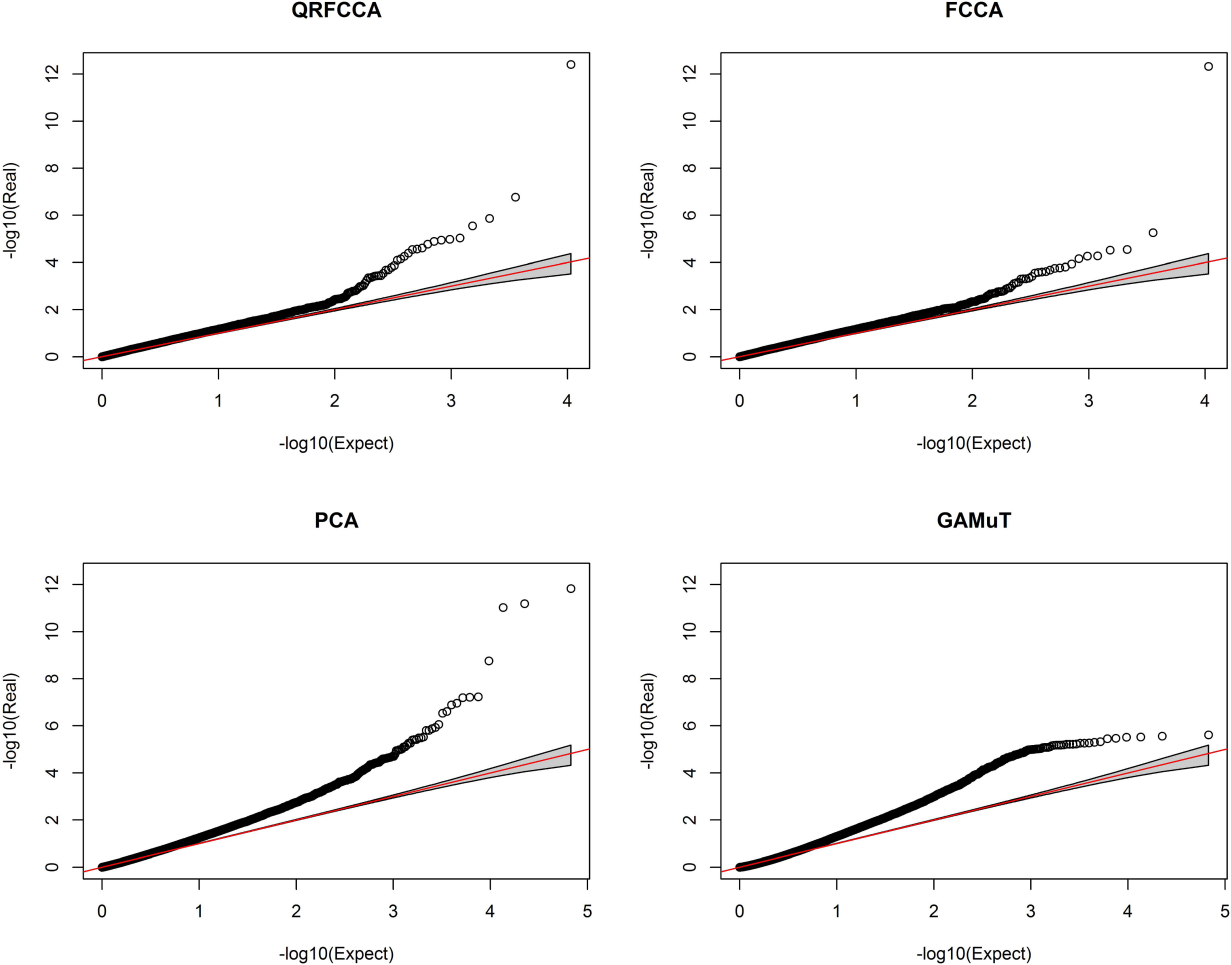
QQ plot of QRFCCA, FCCA, PCA and GAMuT with 95% confidence interval for rare variants. The negative logarithm of the observed (*y* axis) and the expected (*x* axis) P value is plotted for each gene (dot), and the red line indicates the null hypothesis of no true association.

We first studied the association of genes with only rare variants (MAF ≤ 0.01). The total number of genes with only rare variants tested for association was 33,746. A p-value for declaring significant association after applying the Bonferroni correction for multiple tests was 1.48 × 10^−6^. To examine the behavior of the test statistics, we plotted the QQ plot of the QRFCCA with one FPC, FCCA with one FPC, PCA and GAMuT using a linear kernel in Fig 7 and the QQ plot of the MSKAT, KCCA, SCCA, CCA, MANOVA and USAT in Fig S33, assuming no PC adjustment. The QQ plots showed that the false positive rate of the QRFCCA and FCCA for testing the association of the gene with 46 traits in some degree was controlled. However, the behavior of the QQ plot of KCCA and SCCA was weird.

**Fig 8:**
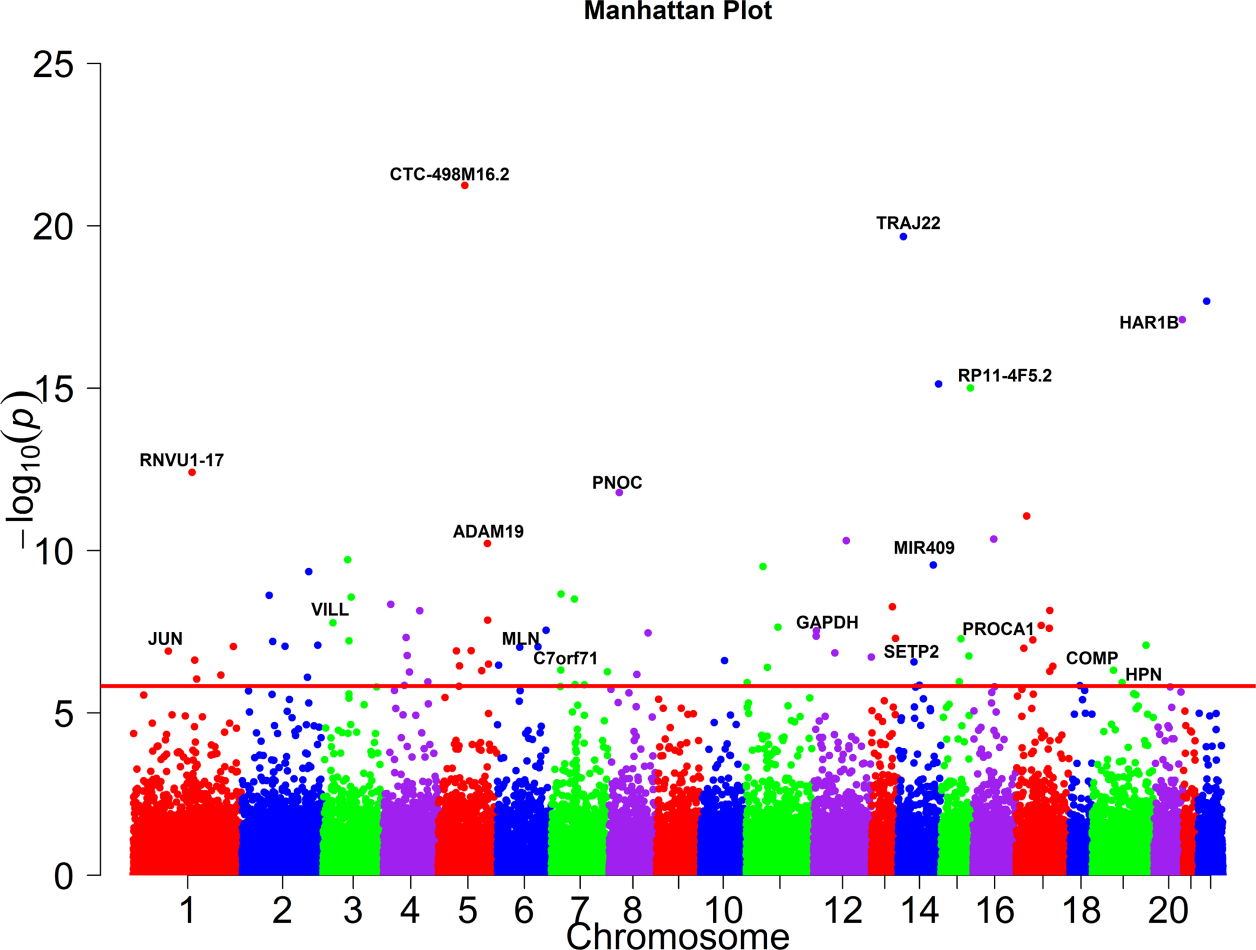
Manhattan plot showing the genome-wide P values of association of the genes consisting of only rare variants with the 46 traits calculated using QRFCCA. The axis *x* represented the chromosomal positions of 33,746 genes and axis *y* showed their −log_10_*P* values. The horizontal red line denotes the thresholds of *P* = 1.48 × 10^−6^ for genome-wide significance after Bonferroni correction.

The total number of genes consisting of only rare variants significantly associated with the 46 traits with and without PC adjustment using QFCCA, FCCA, PCA, SCCA, KCCA, MSKAT, GAMuT, CCA, USAT and MANOVA, were shown in Table 3. A list of P-values of top 25 genes with rare variants only significantly associated with 46 traits using QRFCCA was summarized in Table 4. A list of P-values of 54 remaining genes significantly associated with 46 traits using QRFCCA were summarized in Table S18. We observed that the list of 79 significant genes identified by QFCCA included all 59 significant genes using FCCA, all 8 significant genes using SCCA, all 3 significant genes using KCCA, 19 significant genes using MSKAT, and 8 significant genes using PCA. The Manhattan plot showing genome-wide p-values of association with 46 traits calculated using QRFCCA is presented in Fig 8.

**Table 3:** Number of genes with only rare variants significantly associated with 46 traits.

	QRFCCA	FCCA	GAMuT	SCCA	USAT	MANOVA	CCA	PCA	KCCA	MSKAT
No adjusted	79	59	0	8	0	0	0	14	6	48
Adjusted	77	54	0	0	0	0	0	8	6	41
Overlapped	66	53	0	0	0	0	0	6	6	39
Proportion	83.50%	90%						43%	100%	81%

**Table 4:** A list of P-values of top 25 genes with rare variants only significantly associated with 46 traits using QRFCCA.

	Statistical Method									
Gene	QRFCCA	FCCA	GAMuT	SCCA	USAT	MANOVA	CCA	PCA	KCCA	MSKAT
CTC-498M16.2	5.7E-22	2.9E-19	1.9E-02	1.3E-03	9.3E-01	1.5E-02	4.7E-02	2.6E-01	1.2E-06	2.5E-04
TRAJ22	2.2E-20	7.2E-18	1.0E-05	1.0E-06	8.6E-01	2.6E-01	3.6E-01	1.5E-12	8.6E-07	2.3E-10
AP000351.10	2.1E-18	3.9E-16	2.5E-02	1.9E-03	6.8E-01	1.7E-01	2.8E-01	7.4E-01	1.3E-06	4.0E-03
HAR1B	7.8E-18	2.5E-15	2.3E-01	1.0E-06	8.4E-01	9.9E-01	9.5E-01	8.3E-02	3.1E-01	1.6E-01
IGHVII-20-1	7.5E-16	1.9E-15	1.1E-01	1.2E-03	9.5E-01	1.1E-01	3.5E-01	2.2E-01	4.5E-03	2.5E-04
RP11-4F5.2	9.9E-16	6.1E-13	1.2E-04	2.1E-03	9.7E-01	4.7E-01	7.2E-01	9.9E-01	3.6E-01	5.5E-05
RNVU1-17	3.9E-13	4.8E-13	6.0E-06	1.2E-03	8.2E-01	7.4E-01	9.9E-01	5.5E-02	3.0E-01	1.0E-07
PNOC	1.6E-12	2.9E-12	5.4E-05	1.0E-06	8.8E-01	3.9E-03	9.0E-02	7.5E-01	3.1E-01	2.5E-08
COTL1P1	8.7E-12	1.4E-11	5.4E-03	7.5E-04	8.2E-01	2.0E-03	1.5E-02	6.2E-01	3.2E-06	4.3E-06
LINC00273	4.4E-11	1.8E-09	1.7E-05	9.0E-04	8.2E-01	9.1E-06	7.1E-05	6.5E-12	2.9E-03	4.9E-13
snoU13	5.0E-11	1.3E-10	9.0E-03	6.9E-03	8.7E-01	2.2E-01	5.8E-01	9.8E-01	1.1E-02	8.2E-04
ADAM19	6.1E-11	8.6E-09	8.2E-01	1.0E-06	8.8E-01	9.7E-01	9.5E-01	1.2E-01	6.9E-01	1.7E-01
CTD-2026G6.2	1.9E-10	9.3E-10	3.2E-02	2.3E-03	9.8E-01	9.3E-01	9.3E-01	6.4E-02	4.1E-01	4.4E-02
MIR409	2.8E-10	5.4E-10	1.3E-01	4.1E-04	1.0E+00	8.9E-04	2.0E-02	2.1E-03	5.1E-03	3.2E-08
RP1-276E15.1	3.1E-10	4.6E-10	6.1E-03	5.2E-03	8.7E-01	9.5E-01	9.7E-01	4.3E-01	8.5E-01	3.3E-03
HMGN1P6	4.5E-10	3.8E-08	8.3E-06	1.0E-02	8.2E-01	9.8E-01	9.3E-01	1.2E-02	4.7E-01	1.1E-05
HOXA7	2.2E-09	3.2E-09	8.8E-02	1.0E-06	9.4E-01	4.2E-01	8.0E-01	7.2E-01	9.9E-03	1.6E-02
RNA5SP99	2.4E-09	1.4E-09	6.3E-04	2.1E-04	9.4E-01	6.9E-01	9.8E-01	8.3E-01	6.5E-01	1.2E-09
AC021660.1	2.7E-09	2.5E-09	7.8E-06	1.8E-04	7.7E-01	6.6E-02	4.4E-01	1.3E-01	9.7E-01	3.4E-04
RP11-561N12.1	3.1E-09	9.1E-08	5.5E-06	1.1E-04	8.9E-01	2.4E-01	8.4E-01	2.1E-02	3.0E-01	3.0E-08
FBXL5	4.5E-09	9.9E-08	6.6E-06	6.8E-04	9.9E-01	9.2E-01	9.9E-01	9.2E-03	3.5E-01	6.4E-04
PPIAP23	5.4E-09	5.5E-09	1.7E-05	4.3E-04	1.0E+00	5.3E-02	4.2E-01	2.9E-05	4.0E-01	2.2E-11
HOXB2	7.0E-09	2.2E-09	9.5E-06	1.0E-06	1.0E+00	8.4E-01	9.1E-01	2.4E-02	6.3E-01	6.1E-09
RP11-170N16.1	7.1E-09	3.6E-07	6.0E-04	6.2E-03	1.0E+00	9.2E-01	9.4E-01	5.5E-05	9.4E-01	1.5E-03
AC008694.3	1.4E-08	5.0E-07	2.4E-06	1.4E-03	9.6E-01	9.3E-01	9.9E-01	1.1E-05	8.8E-01	6.6E-05

To further assess the performance of the QFCCA and GAMuT, we presented Tables S19 and S20. Table S19 summarized the top ten genes ranked using GAMuT where p-values calculated by both GAMuT and QRFCCA were also listed. None of ten genes reached genome-wide significance levels by the GAMuT. However, we noticed that 7 of top ten genes ranked by GAMuT were significantly associated with the 46 traits identified by QFCCA. Although we observed that the p-value of *RNU6-1229P* calculated by GAMuT was smaller than that calculated by QRFCCA, we did not find any significant SNPs within *RNU6-1229P* (Fig S34). This may imply that association was spurious. In Table S20, we listed p-values of all SNPs within gene *ADAM19*. We observed that QRFCCA identified significance *of ADAM19* with a p-value less than 6.07 × 10^−11^ and at least 4 SNPs in *ADAM19* had very small p-values and two additional SNPs had p-values that were close to the threshold p-value of genome-wide significance (Fig S34). However, the GAMuT missed to identify significance of *ADAM19*.

**Fig 9:**
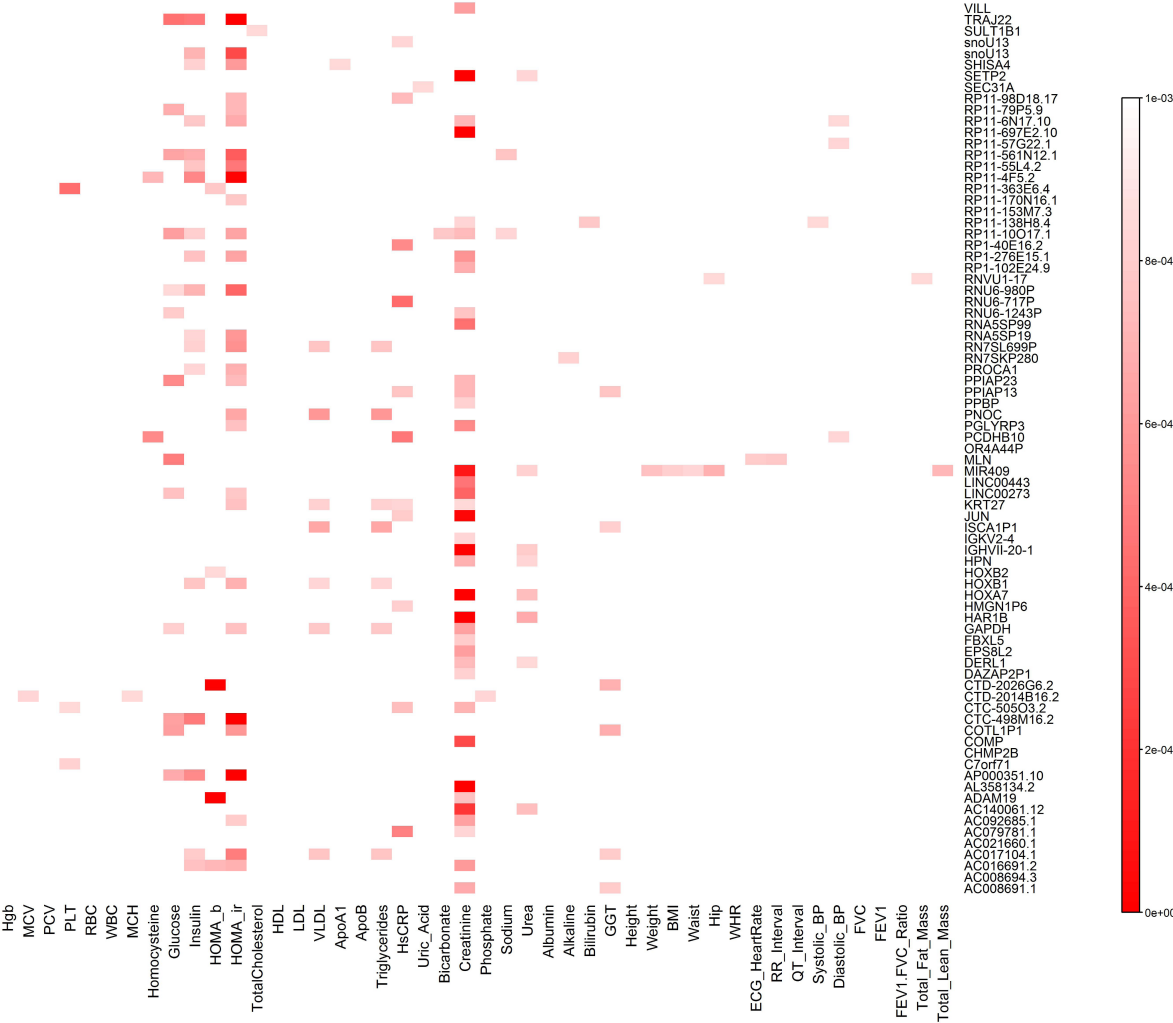
Cross phenotype association heat map (46 traits/79 genes with rare variants only). The horizontal axis denotes the traits and the vertical axis denotes the genes. The color represented P-values. The smaller the P-value the deeper the red color.

To characterize the pleiotropic pattern, we presented the heat map showing the pattern of cross phenotype association of genes with rare variants only and the most important pleiotropic effects of the genes (Fig 9). Table S21 summarizes the number of traits which a single gene was associated with (p-value ≤ 0.05). In Table S21, we also listed the p-values for testing the association of the gene with all 46 traits. All p-values in Fig 9 and Table S21 were calculated using QRFCCA. We observed two remarkable features. First, we observed that 5 genes were significantly associated with 3 traits, 10 genes were significantly associated with 2 traits, and 39 genes were significantly associated with one trait at the genome-wide significance level after Bonferroni correction. The remaining 25 genes did not reach the genome-wide significance with any trait. However, we observed that these genes still showed mild association with multiple traits. Second, we observed that multiple genes were significantly associated with single phenotype (Table S22). For example, 345 genes were significantly associated with creatinine, 108 genes with HOMA-IR, 20 genes with HOMA-B, 72 genes with HsCRP, 21 genes with glucose, 15 genes with insulin, 14 genes with GGT, and 11 genes with VLDL.

Some results can be confirmed in the literature. Throughout this section, all p-values were calculated using QRFCCA. We found that 30 out of 79 genes (38.0%) with rare variants significantly associated with 46 traits were reported association with some of them in the literatures. For example, *PNOC* which was associated with the 46 traits (p-value ≤ 1.63 × 10^−12^), HOMA-IR (p-value ≤ 1.2 × 10^−7^) and triglycerides (p-value ≤ 9.15 × 10^−9^) was reported to be associated with insulin resistance [60] and triglycerides [61]. *MIR409* which was associated with the 46 traits (p-value ≤ 2.77 × 10^−10^), weight (p-value ≤ 1.65 × 10^−6^), Hip (p-value ≤ 7.19 × 10^−7^) and total lean mass (p-value ≤ 2.15 × 10^−6^) was used as a weight loss biomarker [62]. *GAPDH* which showed an association with the 46 traits (p-value ≤ 2.96 × 10^−8^) and specifically with creatinine (p-value ≤ 3.45 × 10^−8^) was reported to be associated with creatinine [63]. *MLN* that demonstrated association with the 46 traits (p-value ≤ 9.47 × 10^−8^) and showed a strong association with glucose (p-value ≤ 6.24 × 10^−11^) played an important role in controlling the rise rate of glucose level [64]. *JUN*, which was associated with the 46 traits (p-value ≤ 1.25 × 10^−7^) and showed a strong association with creatinine (p-value ≤ 3.43 × 10^−20^), was reported to be correlated with the serum creatinine level [65]. *COMP* which showed significance with the 46 traits p-value ≤ 4.83 × 10^−7^) and a strong association with specific trait creatinine (p-value ≤ 9.58 × 10^−15^) was also reported an association with creatinine [66].

### Association analysis of common variants

**Fig 10:**
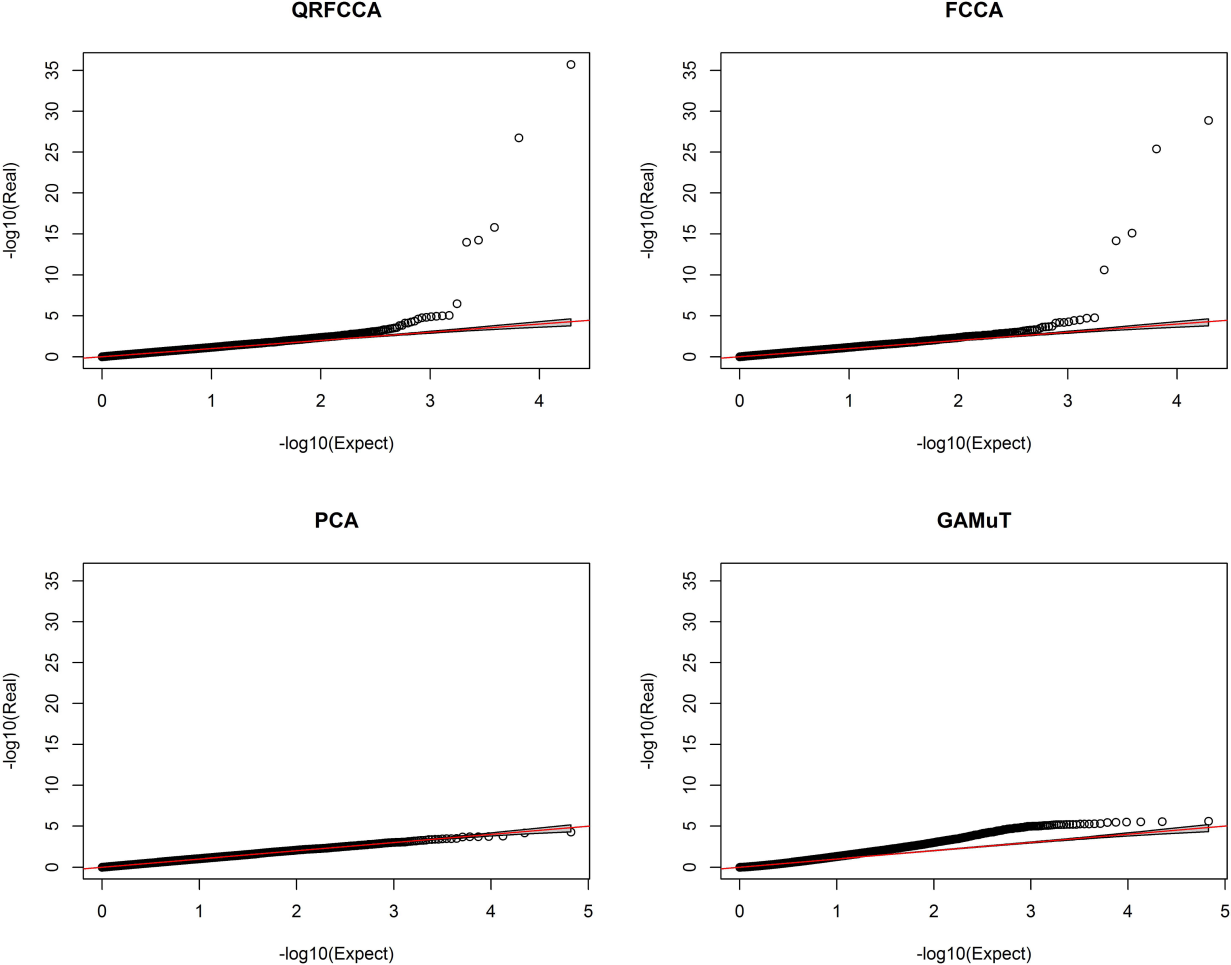
QQ plot of QRFCCA, FCCA, PCA and GAMuT with 95% confidence interval for common variants. The negative logarithm of the observed (*y* axis) and the expected (*x* axis) P value is plotted for each gene (dot), and the red line indicates the null hypothesis of no true association.

Next we studied the association of genes with only common variants (MAF ≥ 0.05). The total number of genes with only common variants tested for association was 33,166. The p-value to declare the significant association after applying Bonferroni correction for multiple tests was 1.51 × 10^−6^. To examine the behavior of the test statistics, we plotted the QQ plot of the QRFCCA, FCCA, PCA and GAMuT using a linear kernel in Fig 10 and the QQ plot of the KCCA, MSKAT, SCCA, CCA, MANOVA and USAT in Fig S35. Similar to rare variants, the QQ plots for common variants showed that the false positive rate of the QRFCCA and FCCA for testing the association of the gene with 46 traits in some degree was controlled. However, the behavior of the QQ plot of KCCA and SCCA was not satisfied.

The total number of genes significantly associated with the 46 trait that were identified using ten statistics with and without PC adjustment were listed in Table 5. Table 6 listed P-value of top 25 genes with common variants only significantly associated with 46 traits that were discovered using QRFCCA. Table S23 summarized remaining 42 genes with common variants only significantly associated with 46 traits that were identified using QRFCCA. Similar to rare variants, we observed that a list of 67 significant genes identified by QRFCCA included all the 55 significant genes identified using FCCA. However, unlike rare variants, only one significant gene was shared between the QRFCCA and KCCA. We observed from Table 5 that the QRFCCA, FCCA substantially outperformed other seven statistics and that the impact of population structure on the QRFCCA, FCCA and KCCA was small.

**Table 5:** Number of genes with only common variants significantly associated with 46 traits.

	QRFCCA	FCCA	GAMuT	SCCA	USAT	MANOVA	CCA	PCA	KCCA	MSKAT
No adjusted	67	55	0	9	0	0	0	0	31	0
Adjusted	62	53	0	0	0	0	0	0	31	0
Overlapped	60	51	0	0	0	0	0	0	31	0
Proportion	90%	93%							100%	

**Table 6:** A list of P-value of 25 genes with common variants only significantly associated with 46 traits using QRFCCA.

	Statistical Method									
Gene	QRFCCA	FCCA	GAMuT	SCCA	USAT	MANOVA	CCA	PCA	KCCA	MSKAT
REG1B	1.6E-116	7.4E-113	4.3E-01	1.3E-03	5.2E-01	1.0E+00	9.2E-01	3.0E-02	9.6E-01	4.0E-01
RP11-665C14.1	1.4E-93	8.2E-75	3.0E-01	4.6E-04	2.6E-01	9.8E-01	1.0E+00	3.9E-01	9.5E-01	2.7E-03
ZNF160	2.0E-91	6.7E-84	4.8E-02	5.6E-05	3.1E-01	9.0E-01	9.4E-01	1.3E-01	9.6E-01	1.1E-01
LEF1	7.4E-83	1.8E-82	2.8E-03	1.0E-06	7.0E-01	9.5E-01	9.7E-01	2.3E-03	9.9E-01	5.9E-01
DYNC1H1	3.5E-58	4.2E-48	5.4E-02	4.1E-03	6.6E-01	9.2E-01	9.4E-01	5.4E-01	9.4E-01	4.0E-03
DOCK7	4.4E-51	6.9E-50	1.7E-01	8.0E-06	9.4E-01	9.1E-01	9.7E-01	1.2E-01	9.5E-01	1.6E-01
SHC3	7.6E-42	2.2E-41	1.4E-02	4.2E-05	8.7E-02	9.7E-01	9.4E-01	1.3E-01	9.9E-01	1.1E-04
Y_RNA	1.9E-36	1.3E-29	4.0E-03	3.5E-05	4.5E-01	1.0E+00	1.0E+00	7.9E-01	9.7E-01	2.5E-03
CTD-2122P11.1	1.6E-33	2.9E-31	7.4E-01	1.5E-05	2.3E-01	9.7E-01	9.8E-01	5.3E-01	9.9E-01	7.6E-03
GBF1	6.3E-28	2.3E-28	6.5E-02	4.5E-04	9.7E-01	9.7E-01	9.7E-01	8.4E-02	9.0E-01	2.4E-02
RP1-8B22.1	1.7E-27	4.1E-26	3.0E-02	7.3E-04	1.8E-01	1.0E+00	1.0E+00	5.0E-02	9.2E-01	2.3E-01
VPS13D	2.6E-26	1.5E-27	7.9E-01	1.9E-03	8.6E-01	9.8E-01	1.0E+00	8.9E-01	9.4E-01	7.6E-01
RP11-68I3.2	3.2E-24	4.9E-24	2.2E-01	1.5E-05	1.0E+00	9.0E-01	9.6E-01	1.6E-02	9.9E-01	3.1E-01
SLC13A3	8.5E-24	2.0E-24	5.3E-01	9.0E-06	4.2E-01	9.9E-01	9.3E-01	7.9E-02	9.4E-01	2.4E-01
RP11-167N24.3	4.3E-23	1.0E-22	7.8E-01	1.1E-03	2.1E-01	9.2E-01	9.2E-01	6.3E-01	9.8E-01	1.0E-01
UBA6	5.3E-22	4.8E-22	2.0E-01	1.2E-03	4.9E-01	9.0E-01	1.0E+00	2.8E-01	9.2E-01	7.9E-01
GAN	1.5E-21	1.0E-20	7.3E-02	3.0E-05	6.6E-01	9.7E-01	9.3E-01	2.9E-01	9.6E-01	1.2E-01
RP4-794H19.2	4.1E-21	1.3E-20	7.5E-01	1.6E-04	8.2E-02	9.3E-01	1.0E+00	9.1E-01	9.5E-01	5.5E-01
RP11-142I20.1	5.3E-21	8.5E-21	1.5E-01	4.3E-05	8.9E-01	9.3E-01	9.9E-01	3.4E-01	9.5E-01	3.1E-02
METAP2	2.6E-20	1.6E-20	6.8E-01	2.5E-05	5.2E-01	9.6E-01	9.4E-01	4.4E-01	9.4E-01	2.2E-04
SLCO1C1	5.9E-20	1.7E-19	7.5E-01	4.0E-06	3.8E-01	9.9E-01	9.1E-01	5.5E-02	9.1E-01	3.4E-03
AC105443.2	2.5E-17	3.4E-17	5.6E-02	1.2E-04	2.5E-01	9.6E-01	9.7E-01	2.6E-02	9.8E-01	1.8E-04
GRN	1.6E-16	7.8E-16	5.0E-01	1.0E-03	1.2E-01	1.0E+00	1.0E+00	3.5E-02	9.6E-01	5.2E-01
INTS12	1.7E-16	1.2E-16	1.8E-02	1.0E-06	4.8E-01	9.7E-01	9.6E-01	3.3E-01	9.5E-01	7.0E-01
RP11-323I15.5	9.3E-16	2.0E-16	1.6E-01	5.0E-06	6.7E-01	9.7E-01	9.5E-01	3.5E-01	9.1E-01	6.5E-01

**Fig 11:**
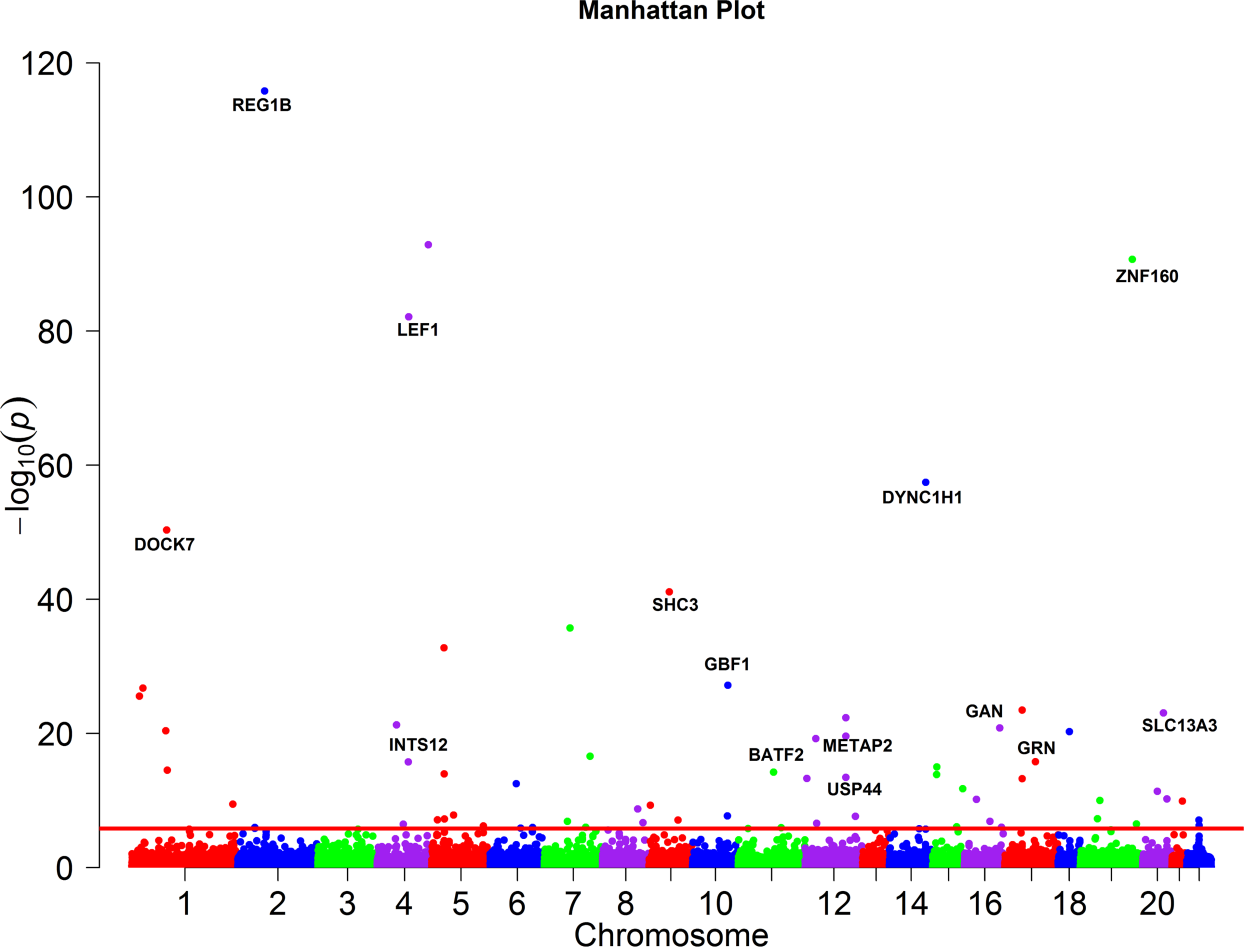
Manhattan plot showing the genome-wide P values of association of the genes consisting of only common variants with the 46 traits calculated using QRFCCA. The axis *x* represented the chromosomal positions of 33,746 genes and axis *y* showed their −log_10_*P* values. The horizontal red line denotes the thresholds of *P* = 1.51 × 10^−6^ for genome-wide significance after Bonferroni correction.

To assess whether the QRFCCA for testing the association of genes including common variants only with multiple traits was appropriate or not, we presented Fig S36 which shows the p-values of all SNPs within gene *REG1B*. We observed in Fig S36 that more than 11 SNPs in *REG1B* with p-values ≤ 0.0001 jointly made contributions to the strong association of *REG1B* with the 46 traits with p-value ≤ 1.65 × 10^−116^. The QRFCCA can catch the features of genetic variation. In Fig S36 we listed the p-values of all SNPs within the gene *XXbac-BPG154L12*.*4*. From Fig S36, we also observed that although the GAMuT treated *XXbac-BPG154L12*.*4* as associated with the 46 traits (p-value ≤ 2.55 × 10^−6^), none of SNPs were even weakly associated with the 46 traits. We should point out that the QRFCCA did not find any, even mild association (p-value ≤ 0.3175). The Manhattan plot showing genome-wide p-values of association of genes consisting of only common variants with the 46 traits calculated using QRFCCA was presented in Fig 11.

**Fig 12:**
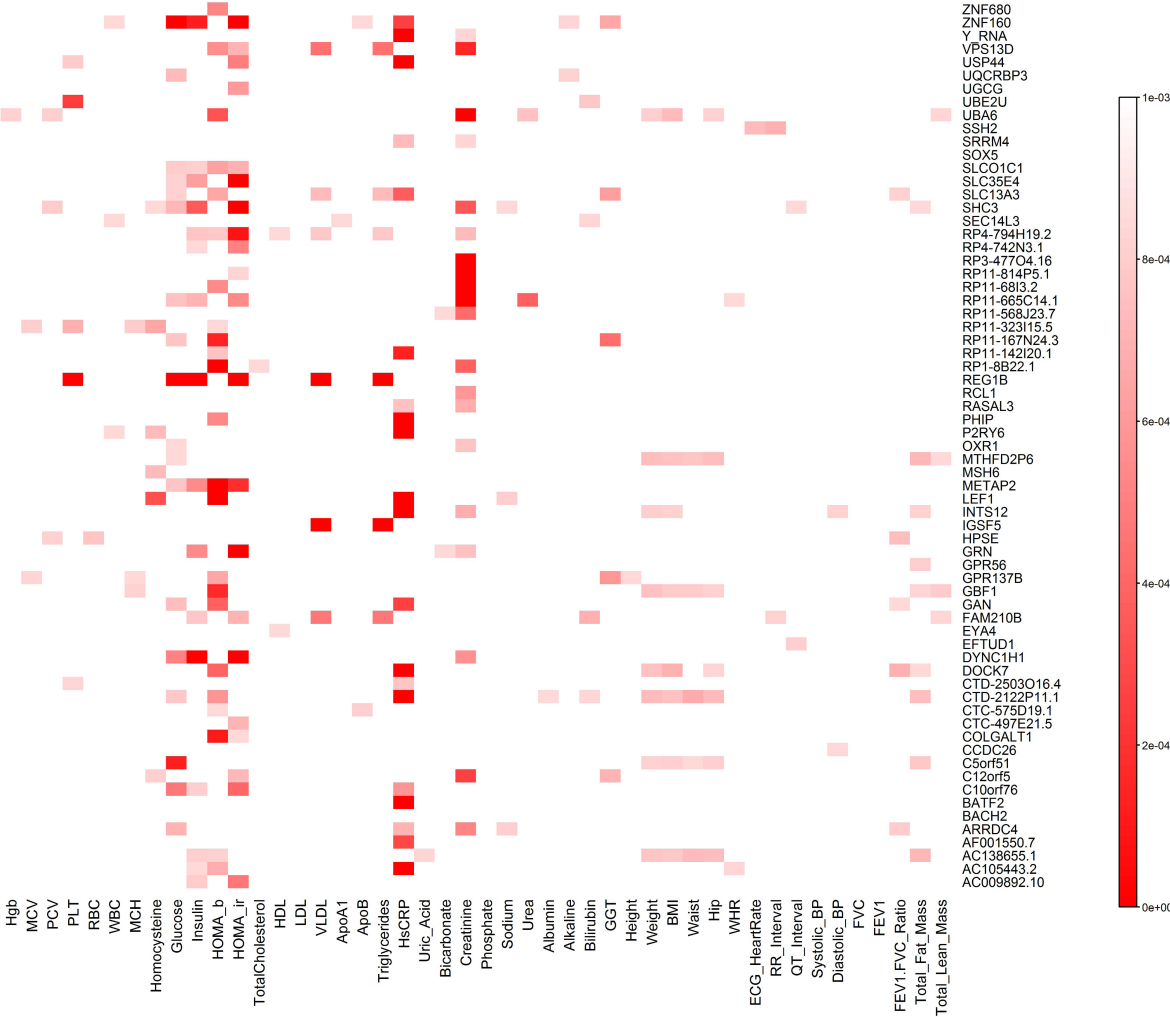
Cross phenotype association heat map (46 traits/67 genes with common variants only). The horizontal axis denotes the traits and the vertical axis denotes the genes. The color represented P-values. The smaller the P-value the deeper the red color.

To unravel the genetic pleiotropic structure of common variants, we presented the gene/phenotype association heat map that demonstrated the most important pleiotropic relations between a single gene and multiple traits (Fig 12) and summarized the number of traits a single gene affected in Table S24. All p-values in Fig 12 and Table S24 were calculated using QRFCCA. We observed that one gene significantly influenced 6 traits; 2 genes, 5 traits; 4 genes, 4 traits; 6 genes, 3 traits; 17 genes, 2 traits and 20 genes, one trait. The remaining 17 genes did not reach the genome-wide significance with any trait. However, we observed that these genes still made genetic contributions to multiple traits. The significant association of the remaining 17 genes with the 46 traits was due to summation of the mild genetic effects on multiple traits of a single gene.

We also analyze the association of all common SNPs in one gene with one trait for all the 46 traits. The results were summarized in Table S25. We observed that 34 genes were significantly associated with creatinine, 29 genes with HsCRP, 23 genes with HOMA-IR, 23 genes with HOMA-B, 9 genes with glucose, 8 genes with insulin, 7 genes with GGT, and 6 genes with VLDL. The distributions of the number of genes consisting of only common variants associated with traits were similar to that of rare variants although the number of genes associated with common variants was smaller than the number of genes associated with rare variants.

Finally, we reported computation times of whole genome association testing for 10 test statistics as in Table 7 where Intel(R) Xeon(R) CPU E7- 4870 @ 2.40GHz was used for calculations. We observed from Table 7 that less than 2 and half hours to complete whole exome association analysis of 46 traits were needed for QRFCCA. We also observed that the computational times of QRFCCA were much less than that of SCCA, USAT, MANOVA, GAMuT and CCA, but larger than that of PCA,KCCA and MSKAT.

**Table 7:** Computational time (seconds)of 10 statistics for gene-based whole genome association analysis of 46 traits.

	QRFCCA	FCCA	GAMuT	SCCA	USAT	MANOVA	CCA	PCA	KCCA	MSKAT
Rare	8629	8471	85176	11036838	691793	39766	39863	1550	2248	2014
Common	9037	8895	87245	11246642	724529	40021	39964	1498	2358	2105

Throughout this section, all genes included common variants only in the analysis. The literature confirmed many gene-trait associations which were identified in this study. We found that 58.2% of identified genes (39 out of 67 genes) with common variants only were reported association with some of 46 traits in the literatures. For example, *REG1B*, which showed the most significant association with the 46 traits (p-value ≤ 1.65 × 10^−116^), has been reported to be associated with glucose [67] (our analysis identified an association with p-value ≤ 1.31 × 10^−21^), the production of insulin [68] (our analysis identified an association with insulin with p-value ≤ 1.81 × 10^−24^ and HOMA-IR with p-value ≤ 5.02 × 10^−94^), and triglyceride whose increment has deleterious effects on the function of islet beta cells [69] (our analysis showed an association with triglyceride with p-value ≤ 1.81 × 10^−24^). *LEF1*, which was associated with the 46 traits (p-value ≤ 7.44 × 10^−83^), has been related to insulin resistance [70] (the analysis demonstrated an association with HOMA-B with p-value ≤ 7.17 × 10^−52^) and heart failure [71–73] (the analysis showed an association with HsCRP with p-value ≤ 1.22 × 10^−122^ and Homocysteine with p-value ≤ 2.24 × 10^−14^). *DYNC1H1*, which was associated with the 46 traits (p-value ≤ 3.46 × 10^−58^), HOMA-IR (p-value ≤ 4.76 × 10^−108^), insulin (p-value ≤ 2.83 × 10^−28^), glucose (p-value ≤ 1.07 × 10^−10^) and creatinine (p-value ≤ 2.57 × 10^−9^), has been associated with hyperinsulinemia, hyperglycemia, the progress of glucose intolerance [74], and the blood creatinine level [75]. *DOCK7*, which presented associations with the 46 traits (p-value ≤ 4.42 × 10^−57^), HsCRP (p-value ≤ 3.66 × 10^−53^) and BMI (p-value ≤ 9.36 × 10^−7^), has been associated with heart disease and ischemic stroke [76] and overweight and obesity[77]. Gene *GBF1*, which was associated with the 46 traits (p-value ≤ 6.30 × 10^−28^), HOMA-B (p-value ≤ 2.91 × 10^−17^), has been reported to be involved in insulin resistance and type 2 diabetes [78]. *METAP2*, which showed strong association with the 46 traits (p-value ≤ 2.62 × 10^−20^), HOMA-B (p-value ≤ 3.14 × 10^−32^), HOMA-IR (p-value ≤ 3.17 × 10^−17^) and insulin (p-value ≤ 8.61 × 10^−10^), has demonstrated associations with insulin resistance and insulin levels [79]. Gene *GRN*, which presented associations with the 46 traits (p-value ≤ 1.63 × 10^−16^), HOMA-IR (p-value ≤ 2.28 × 10^−29^) and insulin (p-value ≤ 7.89 × 10^−10^), has been reported to associate with insulin resistance in type 2 diabetes patients [80] and the blood insulin levels [81]. Finally, gene *USP44*, which showed associations with the 46 traits (p-value ≤ 3.49 × 10^−14^), HsCRP (p-value ≤ 1.81 × 10^−31^) and HOMA-IR (p-value ≤ 7.75 × 10^−11^), has been reported to associate with congenital heart disease [82], the increment of the HsCRP in congenital heart disease patient and insulin resistance [83].

## Discussion

Investigating the pleiotropic effects of the genetic variants can provide important information to allow a deeper understanding of the complex genetic structures of health and disease. However, the identification of complete pleiotropic structures of high dimensional genotype-phenotypes poses great statistical and computational challenges. To meet these challenges, we have addressed several issues to overcome the critical barriers in advancing the development of novel statistical methods and computational algorithms for genetic pleiotropic analysis.

The first issue is to explore deep architectures of genotype-phenotype data in cross-phenotype association analysis. The traditional single trait and multiple trait analysis usually use genotype data in their raw form. These methods do not transform the raw data into a suitable internal representation in which association analysis can be used for distinguishing disease patterns from health patterns. DNA sequences and genetic variants are highly correlated and hierarchically organized in the genome. Exploring multiple levels of representation of the genetic variants and efficiently using correlation information in the data can increase the power to detect the association of the genetic variants with phenotypes. Multiple levels of representation of genetic variants consist of several steps: (1) FPCA, (2) matrix factorization, (3) quadratic regularization, and (4) CCA. The FPCA changes the raw genetic variants to the functional principal component representation that captures the linkage disequilibrium features. The matrix factorization is to embed the functional principal component scores into the low dimensional vector space. It compresses the functional principal component score data to a few new features that are another level of representation of genetic variants. Quadratic regularization further compresses the data and changes the representation of functional principal component scores. Finally, CCA is used as an effective tool for two-view dimension reduction. QRFCCA combines dimension reduction in different levels of data representations. Multiple levels of representation of genetic variants are effective at leveraging data structure. This can be evidenced from the analysis of less derived traits. Our results showed that the significance decreased when the derived traits were removed from the analysis. Utilizing the data structure is a key component of the proposed QRFCCA method. Using less derived traits will decrease the dependence among traits and hence the QRFCCA analysis will use less correlation information among the traits and hence slightly reduce the significance. Multiple levels of representation of genetic variants are a philosophy. We borrow this concept from deep learning methods that are representation methods with multiple levels of representations, each transferring the representation at one level into a representation at another level.

Specifically, to fully utilize the linkage disequilibrium information of genetic variants across the genomic region and efficiently reduce the dimension of the data, we proposed a new paradigm of association analysis that consists of three steps to combine multilevel data reduction and CCA. The first step is to apply FPCA to the original data for dimension reduction. The FPCA decomposes the genetic data into several functional components. Each functional component contains functional information of genetic variants across the genomic region, preserves the orders of genetic variants along the genomic and returns all possible pair-wise and high order linkage disequilibrium. If the phenotypes are function-valued physiological traits or RNA-seq data, the FPCA can also be applied to the phenotype data. The second step is to use quadratically regularized matrix factorization for further compressing the FPC scores into low rank representation and removing noisy data points. As a result, the FPCA and matrix factorization extracted useful genetic and phenotype information and deeply learned the internal genetic and phenotype representation. The third step is to apply CCA to the extracted FPC scores. Large scale simulations and real data analysis demonstrated that QRFCCA substantially outperformed all ten other statistics and FCCA outperformed some of multivariate statistics.

The second issue is to develop a general framework for unifying association analysis, which provides a theoretic basis to evaluate various statistical methods for association analysis and design the guidance for developing novel statistics for testing the association of genetic variants with phenotypes. We used reproducing kernel Hilbert spaces (RKHS) as a general framework and the covariance operator as a general tool for unifying CCA, kernel CCA, functional CCA, dependence measure-based independence tests and other association analyses including GAMuT. We showed that multivariate linear regression are equivalent to the classical CCA. Covariance is a key measure to assess linear association. Its extension to covariance operator provides a tool for quantifying the nonlinear association and derives kernel-based dependence measures and independence tests which form the basis for the GAMuT test. We also show that the KCCA is quite similar to the kernel independent test. Finally, we considered the FCCA. To unify multivariate association tests and functional association tests, we used RKHS as a general framework for the formulation of the functional CCA. We showed that the dependence measured in the FPC score-based kernel analysis is asymptotically equal to the association measure of the FCCA. The FCCA also use the kernel-based dependence measure to develop association tests. Unlike the KCCA and GAMuT test where the kernels are selected by users, the FCCA uses the FPC scores as the feature space and derives the kernels from the data. This was why large-scale simulations and real data showed that in general, the FCCA outperformed the GAMuT, MSKAT and KCCA. Our FPC scores were generated via two steps. The first step used Fourier or wavelet expansions to derive eigenfunctions. The second step was to generate the FPC scores from the eigenfunctions. In the literature, some authors used one step FPCA to directly derive FPC scores from the Fourier or wavelet expansions. Our experiences showed that the number of functional principal components using two step FPCA was, in general, smaller than that directly derived from the Fourier or wavelet expansions. Therefore, two step FPCA had higher power than the one step FPCA.

The third issue is how to reveal pleiotropic structure of the genetic variants and quantify the degree of pleiotropy. Pleiotropy is a widely used word to indicate that a gene affects multiple traits. However, the nature and extent of pleiotropy is less precisely defined. Recently, Schaid et al, 2016 [84] gave the formal testing for pleiotropy. They proposed that formal test of pleiotropy should assume a null hypothesis that one or fewer traits are associated with a genetic variant. Unlike the definition of Schaid et al. our null model of the pleiotropy of a gene is the absence of any traits which the gene was associated with. If Schaid et al (2016) definition of a formal test of pleiotropy is used the proposed test is not a pleiotropy tool.

The fourth issue is the cross-phenotype association analysis with next-generation sequencing. The popular methods for cross-phenotype association analysis is to assess the influence of a single variant on multiple distinct phenotypes. These methods work very well for cross-phenotype association analysis of common variants, but are not suitable for testing the association of rare variants with multiple phenotypes. To illustrate the urgent need to develop gene-based statistical methods for cross-phenotype association analysis of rare variants, we searched the variants across the genome for significant associations with the multiple phenotypes. We found that 21,272 rare variants were significantly associated with the 46 traits at the genome-wide significance level after Bonferroni correction. It is highly unlikely that so many rare variants affected the 46 traits. To overcome this limitation, we developed the QRFCCA for gene-based cross-phenotype association analysis. The QRFCCA can be applied to both multivariate phenotypes, function-valued phenotypes and NGS genotype data. Since the genotype profiles of the common variants and rare variants have different patterns, to increase the power of the tests, we take the association tests of common variants and rare variants separately. We found that the significant genes with common variants only were not overlapped with the significant genes with rare variants only. In pleiotropic analysis, we should conduct cross-phenotype analysis for both common and rare variants and separately. The QRFCCA provides a powerful tool to accomplish this task.

To provide a guidance for cross phenotype association studies, we comprehensively evaluated the current existing statistics for cross-phenotype association by using large-scale simulations and real data analysis. In all simulated cases, when the sample size reached 2,000, the power of the QRFCCA varies between 80% and 90%. We found that the proposed QRFCCA not only substantially outperformed all other widely used competing statistics, but also was very flexible. The QRFCCA can be used for association analysis of both common variants and rare variants, and any phenotypes including quantitative or qualitative, multivariate or function-valued phenotypes.

We performed cross-phenotype association analysis of a largest number of traits with NGS data up to the present time. We identified 79 genes including rare variants only which were significantly associated with the 46 traits and 67 genes including common variants only which were significantly associated with the 46 traits. These two sets of genes were not overlapped. Some of gene-phenotype association can be confirmed in the literature. We found that the largest number of the traits which a gene significantly affected at the genome-wide significance level was six and three in the cross-phenotype association analysis of common and rare variants, respectively. We also discovered that the largest number of traits which a gene affected with the P-value < 0.05 was 18 and 16 in the cross-phenotype association analysis of common and rare variants, respectively. In the single trait association analysis, we found that a large number of genes significantly affected creatinine (genes with rare variants: 345, genes with common variants: 34), HsCRP (genes with rare variants: 72, gene with common variants: 29) and HOMA-IR (genes with rare variants: 108, genes with common variants: 24).

The results presented in this paper are preliminary. The greatest lengths of the genes that were significantly associated with the 46 traits for rare and common variants in the real data analysis were 131Kb and 42Kb, respectively. The proposed methods may not have power to detect the association of the genes with lengths longer than these numbers. The number of basis functions for genotype profile expansion is an important factor for the power of the FPCA-based tests. We have not performed theoretical analysis to determine the appropriate number of basic functions for genotype profile expansions. We resort to ad hoc approaches to select the number of basis function in the expansions. The current pleiotropic analysis cannot identify the global causal structure of pleiotropy, which will decrease our power to unravel mechanisms underlying complex traits. To overcome this limitation, causal inference tools should be explored for cross-phenotype association analysis. The purpose of this paper is to stimulate further discussions regarding the great challenges we are facing in the pleiotropy analysis of high dimensional phenotypic and genomic data produced by modern sensors and next-generation sequencing.

## Supporting information

S1 Text Supplemental note. (PDF)

S1 Fig Fig S1–Fig S36.
(PDF)

S1 Table Table S1–Table S25.
(PDF)
