# Supplementary material for: A quadratically regularized functional canonical correlation analysis for identifying the global structure of pleiotropy with NGS data
Source: PLoS Comput Biol. 2017 Oct 17;13(10):e1005788. doi: 10.1371/journal.pcbi.1005788 (PMC5659802; doi:10.1371/journal.pcbi.1005788)
Supplement: S1 Text — (PDF) [file pcbi.1005788.s001.pdf]

Supplemental Note

Supplemental Note A

Quantitative Genetics for Multiple Traits

Single Trait

We first review quantitative genetics for a single trait. For simplicity, we only consider genetic additive effects. It is straightforward to extend analysis to include the genetic dominance effects. Consider the genetic model for a trait and a single locus:

$$Y = X\alpha + \varepsilon , \quad \text{(N1)}$$

where Y denotes a trait, X an additive genotype score, α the genetic additive effect and ε error with mean zero and variance σ_e^2.

The genetic additive effect is estimated by

$$\hat{\alpha} = \frac{\operatorname{cov}(x, y)}{\operatorname{var}(x)} . \quad \text{(N2)}$$

The genetic additive variance σ_A^2 and narrow-sense heritability are

$$\sigma_A^2 = \frac{[\operatorname{cov}(x, y)]^2}{\operatorname{var}(x)} , \quad \text{(N3)}$$

and

$$h^2 = corr^2(Y, X) , \quad \text{(N4)}$$

respectively.

Equation (N4) shows that the narrow heritability can also be expressed as the proportion of the phenotype variation explained by the genetic variation or the squared correlation between the genotype and phenotype.

Multiple Traits

Quantitative genetics for a single trait can be easily extended to multiple traits. Again consider a genetic model for multiple traits and multiple loci:

$$Y = XB + E, \qquad \text{(N5)}$$

where $Y = [Y_1,...,Y_k]$ represent a vector of k phenotype variables, X is a vector of p genotype variables, $B = [b_1,...,b_k], b_j \in R^p$ the matrix of genetic additive effects and $\varepsilon = [\varepsilon_1,..., \varepsilon_k]$ is a vector of k error variables.

The genetic effect matrix B can be estimated by

$$\hat{B} = \Sigma_{xx}^{-1}\Sigma_{xy}. \qquad \text{(N6)}$$

The covariance of the genetic additive effects is defined as

$$\Sigma_A = \Sigma_{yx}\Sigma_{xx}^{-1}\Sigma_{xy}. \qquad \text{(N7)}$$

The concept of heritability for a single trait is well developed. Recently, there has been an increasing attempt to extend heritability from a single trait to multiple traits. However, their focus is to consider the heritability of a linear combination of multiple traits. Here, we consider a heritability matrix that is defined as

$$h_m^2 = \Sigma_{yy}^{-1/2}\Sigma_{yx}\Sigma_{xx}^{-1}\Sigma_{xy}\Sigma_{yy}^{-1/2}. \qquad \text{(N8)}$$

It is clear that the heritability matrix h_m^2 is equal to the matrix R^2 in equation (7).

Linear Combination of Multiple Traits

We consider a linear combination of multiple phenotypes Yb and a linear combination of genotypes at multiple loci Xa to transform the association analysis of multiple traits to the association analysis of single trait. Define the linear genetic model for Yb and Xa:

$$Yb = (Xa)\alpha + \varepsilon . \qquad \text{(N9)}$$

The total genetic effect of the multiple genotypes on the multiple traits can be estimated by

$$\hat{\alpha} = \frac{\operatorname{cov}(Xa, Yb)}{\operatorname{var}(xa)} = \frac{a^T \Sigma_{xy} b}{a^T \Sigma_{xx} a} . \qquad \text{(N10)}$$

Using equations (N3) and (N4), we obtain the genetic additive variance of Xa and heritability of yb:

$$\sigma_{Al}^2 = \frac{(a^T \Sigma_{xy} b)^2}{a^T \Sigma_{xx} a} , \qquad \text{(N11)}$$

and

$$h_l^2 = \frac{(a^T \Sigma_{xy} b)^2}{b^T \Sigma_{yy} b a^T \Sigma_{xx} a} , \qquad \text{(N12)}$$

respectively.

It is clear that the squared multiple correlation coefficient is given by

$$R^2 = \frac{(a^T \Sigma_{xy} b)^2}{b^T \Sigma_{yy} b a^T \Sigma_{xx} a} = h_l^2 .$$

Next we seek the optimal combinations of the genotypes at multiple loci and the multiple traits to maximize the genetic additive effect, genetic additive variance and heritability. We first find the maximum genetic additive effect.

Using equation (N10) and the Lagrangian multiplier method, we can solve the following optimization problem to obtain the maximum genetic additive effect:

$$L(a,b,\lambda) = a^T \Sigma_{xy} b + \frac{\lambda}{2}(1 - a^T \Sigma_{xx} a)\,, \qquad \text{(N13)}$$

where λ is a multiplier.

Setting $\frac{\partial L(a,b,\lambda)}{\partial a} = \Sigma_{xy} b - \lambda \Sigma_{xx} a = 0$ gives

$\Sigma_{xy} b = \lambda \Sigma_{xx} a$ or

$$\Sigma_{xx}^{-1} \Sigma_{xy} b = \lambda a\,. \qquad \text{(N14)}$$

Suppose that the SVD of the matrix $\Sigma_{xx}^{-1}\Sigma_{xy}$ is given by

$$\Sigma_{xx}^{-1}\Sigma_{xy} = U_e \Lambda_e V_e^T\,. \qquad \text{(N15)}$$

Then, it follows from equation (N15) that a , b and the optimal genetic effect are the left, right singular vectors and singular value of $\Sigma_{xx}^{-1}\Sigma_{xy}$, respectively. Similarly, we can show that the genetic additive variance σ_{Al}^2 is the square of the singular value of $\Sigma_{xx}^{-1}\Sigma_{xy}$. The maximum heritability can be obtained by setting the Lagrange function:

$L(a,b,\lambda,\mu) = (a^T \Sigma_{xy} b)^2 + \lambda(1 - a^T \Sigma_{xx} a) + \mu(1 - b^T \Sigma_{yy} b)$ and

$$\frac{\partial L}{\partial a} = 2(a^T \Sigma_{xy} b)\Sigma_{xy} b - 2\lambda \Sigma_{xx} a = 0$$
$$\frac{\partial L}{\partial b} = 2(a^T \Sigma_{xy} b)\Sigma_{yx} a - 2\mu \Sigma_{yy} b = 0 . \qquad \text{(N16)}$$

Let

$K = \Sigma_{xx}^{-1/2} \Sigma_{xy} \Sigma_{yy}^{-1/2}$ and SVD of K be

$$K = U_K \Lambda_K V_K^T , \qquad \text{(N17)}$$

where $U_K = [u_1,...,u_q]$, $\Lambda_K = diag(\tau_1,...,\tau_q)$ and $V_K = [v_1,...,v_q]$.

Solving equation (N16) we obtain

$$a_i = \Sigma_{xx}^{-1/2} u_i , \; i = 1,...,q,$$
$$b_j = \Sigma_{yy}^{-1/2} v_j , \; j = 1,...,q, \qquad \text{(N18)}$$
$$h_{lk}^2 = \tau_k^2 ,$$

where u_i, v_j and τ_k are the left, right singular vectors and singular value of the matrix K, respectively. Substituting equation (N18) into equations (N10) and (N11) gives

$$\hat{\alpha}_i = u_i^T K v_i$$
$$\sigma_{AI_i}^2 = \hat{\alpha}_i^2 . \qquad \text{(N19)}$$

Note that τ_k^2 is the eigenvalue of the matrix

$$K^T K = \Sigma_{yy}^{-1/2} \Sigma_{yx} \Sigma_{xx}^{-1} \Sigma_{xy} \Sigma_{yy}^{-1/2} . \qquad \text{(N20)}$$

It follows from equation (N20) and (18) that

$$K^T K = R^2 .$$

This shows that the maximum heritability analysis is equivalent to CCA.

Supplemental Note B

RKHS framework for Functional CCA, Cross-covariance operator, Dependence Measure and Independence Test

Introduction of RKHS

Many multivariate and functional statistical methods such as regression, CCA, kernel regression, kernel CCA, functional regression and functional CCA, dependence measure can be used to test the association of genetic variants with the phenotypes. In the past decades, reproducing kernel Hilbert spaces (RKHS) have emerged as a general framework for various statistical and machine learning methods. Here, we propose to use RKHS as a unified framework for association tests to reveal the relationships among various multivariate and functional association tests.

We begin with briefly introducing RKHS [1,2]. Let $\mathcal{H}$ be a Hilbert space of functions on a non-empty set χ and denote the inner product in $\mathcal{H}$ by $< .,. >_{\mathcal{H}}$. A bivariate function K on $\chi \times \chi$ is called a reproducing kernel for $\mathcal{H}$ if K satisfies

(1) For every $t \in \chi$, $K(.,t) \in \mathcal{H}$ and (N21)

(2) For every $t \in \chi$ and $f \in \mathcal{H}$, we have $f(t) =< f, k(.,t) >_{\mathcal{H}}$. (N22)

We call $\mathcal{H}$ a RKHS with reproducing kernel K.

Define

$$K(s,t) = E[X(s)X(t)] \quad \text{(N23)}$$

be a covariance function that generates a RKHS.

A powerful analytical tool for CCA and independence tests is the cross-covariance operator that is an extension of the covariance matrix to infinite dimensional space [3]. Recall that the covariance matrix is defined as

$$\tilde{\Sigma}_{XY} = E[XY^T], \qquad \text{(N24)}$$

where X and Y are vectors of random variables with $E[X]=0$ and $E[Y]=0$. Equation (N24) can be extended to feature space. Let $\phi(X)$ and $\psi(Y)$ be feature maps. In the feature space, equation (N24) can be written as

$$\tilde{\Sigma}_{XY} = E[\phi(X)\psi^T(Y)]. \qquad \text{(N25)}$$

Let f and g be vectors in the feature space. Recall that by the reproducing property, we have

$$f(X) = < f(.), K(., X) > \text{ and } g(Y) = < g(.), K(., Y) > . \qquad \text{(N26)}$$

Define kernels $K(., X) = \phi(X)$ and $K(., Y) = \psi(Y)$. Viewing the covariance matrix $\tilde{\Sigma}_{XY}$ as an operator and applying it to the vector g, we obtain

$$\begin{aligned} \tilde{\Sigma}_{XY} g &= E[\phi(X)\psi^T(Y)g(.)] = E[\phi(X) < K(., Y), g(.) >] \\ &= E[\phi(X)g(Y)]. \end{aligned} \qquad \text{(N27)}$$

Equation (N27) indicates that $\tilde{\Sigma}_{XY} g$ maps g to a vector in the feature space spanned by $\phi(X)$. Let f be a vector in the feature space. Then, its inner product with $\tilde{\Sigma}_{XY} g$ is given by

$$\begin{aligned} f^T \tilde{\Sigma}_{XY} g &= E[f^T \phi(X) g(Y)] \\ &= E[< f(.), K(., X) > g(y)] \\ &= E[f(X)g(Y)]. \end{aligned} \qquad \text{(N28)}$$

In terms of kernels, equation (N28) can be written as

$$f^T \tilde{\Sigma}_{XY} g = E[< f(.), k(., X) >< K(Y,.), g(.) >]. \quad \text{(N29)}$$

We assume that

$$f = \sum_{j=1}^{m} \alpha_j \phi(X_j) \text{ and } g = \sum_{l=1}^{m} \beta_l \psi(Y_l). \quad \text{(N30)}$$

From equations (N27) and (N28) we can obtain the sampling formula for $f^T \tilde{\Sigma}_{XY} g$:

$$f^T \tilde{\Sigma}_{XY} g = \frac{1}{m} \sum_{i=1}^{m} < f(.), K(., X_i) >< K(Y_i,.), g(.) >. \quad \text{(N31)}$$

Substituting equation (N30) into equation (N31) gives

$$\begin{aligned} f^T \tilde{\Sigma}_{XY} g &= \frac{1}{m} \sum_{i=1}^{m} < \sum_{j=1}^{m} \alpha_j K(X_j,.), K(., X_i) >< K(Y_i,.), \sum_{l=1}^{m} \beta_l K(Y_l,.) > \\ &= \frac{1}{m} \sum_{j=1}^{m} \sum_{l=1}^{m} \alpha_j \beta_l \sum_{i=1}^{m} K(X_j, X_i) K(Y_i, Y_l) \\ &= \frac{1}{m} \alpha^T K_x K_y \beta, \end{aligned} \quad \text{(N32)}$$

where

$$K_x = \begin{bmatrix} K(X_1, X_1) & \cdots & K(X_1, X_m) \\ \vdots & \vdots & \vdots \\ K(X_m, X_1) & \cdots & K(X_m, X_m) \end{bmatrix} \text{ and } K_y = \begin{bmatrix} K(Y_1, Y_1) & \cdots & K(Y_1, Y_m) \\ \vdots & \vdots & \vdots \\ K(Y_m, Y_1) & \cdots & K(Y_m, Y_m) \end{bmatrix}.$$

Let $G = I_m - \frac{1}{m}\mathbf{1}_m$, where $\mathbf{1}_m$ is a $m \times m$ matrix of ones. The centered covariance is

$$\Sigma_{XY} = \tilde{\Sigma}_{XY} - \mu_X \mu_Y^T,$$

where $\mu_X = E[\phi(X)]$ and $\mu_Y = E[\psi(Y)]$.

Using the similar arguments, we can show

$$f^T \Sigma_{XY} g = \frac{1}{m} \alpha^T \tilde{K}_x \tilde{K}_y \beta , \qquad \text{(N33)}$$

where $\tilde{K}_x = GK_xG$ and $\tilde{K}_y = GK_yG$.

Dependence Measure, Covariance Operator and CCA

The covariance operator is a useful tool for assessing dependence between variables and hence form a foundation for association analysis. A dependence measure can be derived from solving the following optimization problem:

$$\begin{aligned} \max_{\alpha,\beta} \quad & \frac{1}{m} \alpha^T \tilde{K}_x \tilde{K}_y \beta \\ s.t. \quad & \alpha^T \tilde{K}_x \alpha = 1 \\ & \beta^T \tilde{K}_y \beta = 1. \end{aligned} \qquad \text{(N34)}$$

Let $u = \tilde{K}_x^{1/2} \alpha$ and $v = \tilde{K}_y^{1/2} \beta$. Then, the optimization problem (N34) can be transformed to

$$\begin{aligned} \max_{u,v} \quad & \frac{1}{\text{m}} u^T \tilde{K}_x^{1/2} \tilde{K}_y^{1/2} v \\ \text{s.t.} \quad & \text{u}^{\text{T}} u = 1 \\ & v^T v = 1. \end{aligned} \qquad \text{(N35)}$$

Using the Lagrange multiplier approach to solve the optimization problem, we can obtain the eigenequation:

$$\frac{1}{m} \tilde{K}_x^{1/2} \tilde{K}_y^{1/2} v = \lambda u , \qquad \text{(N36)}$$

$$\frac{1}{m} \tilde{K}_y^{1/2} \tilde{K}_x^{1/2} u = \lambda v . \qquad \text{(N37)}$$

Substituting equation (N37) into equation (N36) gives eigenequation:

$$\frac{1}{m^2} \tilde{K}_x^{1/2} \hat{K}_y \tilde{K}_x^{1/2} u = \lambda^2 u . \qquad \text{(N37)}$$

Assume that the single value decomposition of $\tilde{K}_x^{1/2} \tilde{K}_y^{1/2}$ is

$$\tilde{K}_x^{1/2}\tilde{K}_y^{1/2} = U\Lambda V^T \,, \qquad \text{(N38)}$$

where $\Lambda = diag(\rho_1,...,\rho_m)$ with $\rho_1 \geq \rho_2 \geq ... \geq \rho_m$.

After some algebra, we obtain

$$\begin{aligned}\frac{1}{m^2}\sum_{i=1}^{n}\rho_i^2 &= \frac{1}{m^2}\text{Trace}(U^T\tilde{K}_x^{1/2}\tilde{K}_y\tilde{K}_x^{1/2}U) \\ &= \frac{1}{m^2}\text{Trace }(\tilde{K}_x\tilde{K}_y).\end{aligned} \qquad \text{(N39)}$$

It is clear that $\frac{1}{m^2}\text{Trace }(\tilde{K}_x\tilde{K}_y)$ can be used as a measure of dependence. The dependence measure $\frac{1}{m^2}\text{Trace }(\tilde{K}_x\tilde{K}_y)$ is used to test for dependence between two sets of random variables [4] and to test for the association of genetic variants with multiple phenotypes [5].

Kernel CCA

Consider Kernel CCA (KCCA) [6]. Let $\Phi(X) = [\phi(X_1),...,\phi(X_m)]^T$ and $\Psi(Y) = [\psi(Y_1),...,\psi(Y_m)]^T$. The representation of linear combinations of the sampled features in the feature space can be defined as

$U = \Phi^T(X)G\alpha$ and $V = \Psi^T(Y)G\beta$,

where center operator $G = I_m - \frac{1}{m}\mathbf{1}_m$ is defined as before.

The canonical variates are linear combinations of features and hence can be represented by

$a = G\Phi(X)U = G\Phi(X)\Phi^T(X)G\alpha = \tilde{K}_x\alpha$ and $b = G\Psi(Y)V = G\Psi(Y)\Psi^T(Y)G\beta = \tilde{K}_y\beta$,

where $\tilde{K}_x = G\Phi(X)\Phi^T(X)G$ and $\tilde{K}_Y = G\Psi(Y)\Psi^T(Y)G$, which are similar to $\tilde{K}_x$ and $\tilde{K}_y$ defined in equation (N33). The covariance between the canonical variates a and b is

$\text{cov}(a,b) = \alpha^T \tilde{K}_x \tilde{K}_Y \beta$. Similarly, we can obtain the variance of a and the variance of b, respectively, $\text{var}(a) = \alpha^T \tilde{K}_x \tilde{K}_x \alpha$ and $\text{var}(b) = \beta^T \tilde{K}_y \tilde{K}_y \beta$. The KCCA seeks canonical vectors in terms of α and β to optimize

$$\begin{aligned}
\max_{\alpha,\beta} \quad & \frac{1}{m} \alpha^T \tilde{K}_x \tilde{K}_y \beta \\
s.t. \quad & \alpha^T \tilde{K}_x \tilde{K}_x \alpha = 1 \\
& \beta^T \tilde{K}_y \tilde{K}_y \beta = 1.
\end{aligned} \tag{N40}$$

If the constraints in (N40) are replaced by $Var(U) = \alpha^T \tilde{K}_x \alpha = 1$ and $Var(V) = \beta^T \tilde{K}_y \beta = 1$, the optimization problem (N40) can be reduced to

$$\begin{aligned}
\max_{\alpha,\beta} \quad & \frac{1}{m} \alpha^T \tilde{K}_x \tilde{K}_y \beta \\
s.t. \quad & \alpha^T \tilde{K}_x \alpha = 1 \\
& \beta^T \tilde{K}_y \beta = 1,
\end{aligned} \tag{N41}$$

which is exactly the same as the formulation (N34). Similar to equation (22), the association measure in the KCCA is equal to

$$\frac{1}{m^2} \text{Tr}(\Lambda^2) = \frac{1}{m^2} \text{Trace}\,(\hat{K}_x \hat{K}_y)\,. \tag{N42}$$

Functional Association Tests

In this section, we show that the dependence measure in the FPC score-based kernel analysis is asymptotically equal to the association measure of the FCCA and FCCA can be implemented as CCA with FPC scores.

To unify multivariate association tests and functional association tests, we use RKHS as a general framework for formulation of the functional CCA [1]. Consider two index sets E_1 and E_2, and two stochastic processes: $\{X(t), t \in E_1\}$ and $\{Y(s), s \in E_2\}$ with mean zero $E[X(t)] = E(Y(s)] = 0$ for all t, s, auto covariance functions

$R_X(s_1, s_2) = \text{cov}(X(s_1), X(s_2)), \quad R_Y(t_1, t_2) = \text{cov}(Y(t_1), Y(t_2))$, and cross covariance functions

$R_{XY}(s,t) = \text{cov}(X(s), Y(t))$.

Let L_X^2 and L_Y^2 be the Hilbert spaces spanned by the X and Y processes defined as the completion of the set of all linear combinations of the random variables:

$\left\{a : a = \sum_{i=1}^{n} \alpha_i X(t_i), t_i \in E_1, \alpha_i \in R, n \in Z^+\right\}$ and

$\left\{b : b = \sum_{i=1}^{n} \beta_i X(s_i), s_i \in E_2, \beta_i \in R, n \in Z^+\right\}$ under the inner product $< a_1, a_2 >_{L_X^2} = E[a_1 a_2]$ and $< b_1, b_2 >_{L_Y^2} = E[b_1 b_2]$, respectively. The covariance function can be used to define an integral operator:

$$(R_X \beta)(t) = \int_T R_X(s,t)\beta(s)ds \ . \qquad \text{(N43)}$$

By Mercer's theorem [7], the covariance function can be expanded in terms of orthonormal functions:

$$R_X(s,t) = \sum_{i=1}^{\infty} \lambda_i \phi_i(s)\phi_i(t), \qquad \text{(N44)}$$

$$R_Y(s,t) = \sum_{j=1}^{\infty} \mu_j \theta_j(s)\theta_j(t), \qquad \text{(N45)}$$

where

$(R_X \phi_l)(t) = \lambda_l \phi_l(t)$ and $(R_Y \theta_j)(t) = \mu_j \theta_j(t)$.

Using the Karhunen-Loeve representation, the stochastic processes $X(t)$ and $Y(t)$ can be expressed as [1]

$$X(t) = \sum_{i=1}^{\infty} \xi_i \phi_i(t) \text{ and } Y(t) = \sum_{j=1}^{\infty} \eta_j \theta_j(t) , \tag{N46}$$

where ξ_i and η_j are uncorrelated variables with zero means, and variances and covariance

$$\operatorname{var}(\xi_i) = \lambda_i, \operatorname{var}(\eta_j) = \mu_j , \ \operatorname{cov}(\xi_i, \eta_j) = \gamma_{ij} , \tag{N47}$$

which implies

$$R_{XY}(s,t) = \sum_{i=1}^{\infty} \sum_{j=1}^{\infty} \gamma_{ij} \phi_i(s) \theta_j(t) . \tag{N48}$$

The RKHS $\mathcal{H}(R_X)$ generated by the covariance kernel R_X is

$$\mathcal{H}(R_X) = \left\{ \alpha(t) : \alpha(t) = \sum_{j=1}^{\infty} \lambda_j \alpha_j \phi_j(t), \sum_{j=1}^{\infty} \lambda_j \alpha_j^2 < \infty \right\}. \tag{N49}$$

Similarly, we can define

$$\mathcal{H}(R_Y) = \left\{ \beta(t) : \beta(t) = \sum_{j=1}^{\infty} \mu_j \beta_j \theta_j(t), \sum_{j=1}^{\infty} \mu_j \beta_j^2 < \infty \right\}. \tag{N50}$$

The congruence mapping from $\mathcal{H}(R_X)$ to L_X^2 is then given by [8]

$$\Psi_X(\alpha) = \sum_{i=1}^{\infty} \lambda_i \alpha_i < X, \phi_i >_{L^2(T)} . \tag{N51}$$

Similarly, we define

$$\Psi_Y(\alpha) = \sum_{j=1}^{\infty} \mu_j \beta_j < Y, \theta_j >_{L^2(T)} . \tag{N52}$$

Using equations (N51) and (N52), we obtain

$$\mathrm{cov}(\Psi_X(\alpha), \Psi_Y(\beta)) = \sum_{i=1}^{\infty}\sum_{j=1}^{\infty}\lambda_i\mu_j\alpha_i\beta_j \,\mathrm{cov}(< X, \phi_i >_{L^2(T)}, < Y, \theta_j >_{L^2(T)}) . \tag{N53}$$

By definition of the inner product in the $L^2(T)$ space, we have

$$< X, \phi_i >_{L^2(T)} = \int_T X(t)\phi_i(t)dt \text{ and } < Y, \theta_j >_{L^2(T)} = \int_T Y(t)\theta_j(t)dt . \tag{N54}$$

Then, using stochastic integral theory [9], we can connect the covariance between these two inner products with the covariance operator:

$$\mathrm{cov}(< X, \phi_i >_{L^2(T)}, < Y, \theta_j >_{L^2(T)}) = \int_T\int_T \phi_i(s)R_{XY}(s,t)\theta_j(t)dsdt. \tag{N55}$$

Substituting equation (N55) into equation (N53) gives

$$\begin{aligned}\mathrm{cov}(\Psi_X(\alpha), \Psi_Y(\beta)) &= \sum_{i=1}^{\infty}\sum_{j=1}^{\infty}\lambda_i\mu_j\alpha_i\beta_j\int_T\int_T \phi_i(s)R_{XY}(s,t)\theta_j(t)dsdt \\ &= \int_T\int_T (\sum_{i=1}^{\infty}\lambda_i\alpha_i\phi_i(s))R_{XY}(s,t)(\sum_{j=1}^{\infty}\mu_j\beta_j\theta_j)dsdt \\ &= \int_T\int_T \alpha(s)R_{XY}(s,t)\beta(t)dsdt.\end{aligned} \tag{N56}$$

Substituting equation (N46) into equation (N56) yields

$$\mathrm{cov}(\Psi_X(\alpha), \Psi_Y(\beta)) =< (R_{XY}\alpha)(t), \beta(t) >_{L^2(T)}, \tag{N57}$$

where R_{XY} with the property $(R_{XY}\alpha)(t) = \int_T R_{XY}(s,t)\alpha(s)ds =< R_{XY}(.,t), \alpha(.) >_{L^2(T)}$ is a cross covariance operator. Equation (N57) is an extension of equation (N28) to functional space.

Now we formally define CCA for the stochastic process. The canonical correlation can be defined in terms of both L_X^2 and L_Y^2, and $\mathcal{H}(R_X)$ and $\mathcal{H}(R_Y)$:

$$\begin{aligned}&\max_{\substack{\zeta\in L_X^2,\upsilon\in L_Y^2\\ \mathrm{var}(\zeta)=1,\mathrm{var}(\upsilon)=1}} \mathrm{cov}^2(\zeta,\upsilon)\\ &= \max_{\substack{\alpha\in\mathcal{H}(R_X),\beta\in\mathcal{H}(R_Y)\\ \|\alpha\|^2_{\mathcal{H}(R_X)}=1,\|\beta\|^2_{\mathcal{H}(R_Y)}=1}} \mathrm{cov}^2(\Psi_X(\alpha),\Psi_Y(\beta))\end{aligned}. \quad \text{(N58)}$$

Now we consider the direct extension of the CCA from multivariate to functional space. Suppose that expansions of the two functions $\alpha(s)$ and $\beta(t)$ in terms of orthonormal functions $\{\phi_i(s), i=1,2,...\}$ and $\{\theta_j(t), j=1,2,...\}$ are given by

$$\alpha(s)=\sum_{i=1}^{\infty}\alpha_i\phi_i(s) \text{ and } \beta(t)=\sum_{j=1}^{\infty}\beta_j\theta_j(t). \quad \text{(N59)}$$

Define the inner product of functions $\alpha(s)$ with stochastic process $X(s)$ as

$$<\alpha(s),X(s)>_{L^2(T)}=\int_T\alpha(s)X(s)ds. \quad \text{(N60)}$$

Substituting equation (N46) into equation (N60), we obtain

$$\begin{aligned}<\alpha(s),X(s)>_{L^2(T)}&=\sum_{i=1}^{\infty}\xi_i\int_T\alpha(s)\phi_i(s)ds\\ &=\sum_{i=1}^{\infty}\xi_i\alpha_i,\end{aligned} \quad \text{(N61)}$$

where $\alpha_i=\int_T\alpha(s)\phi_i(s)ds$.

Similarly, we have

$$<\beta(t),Y(t)>_{L^2(T)}=\sum_{j=1}^{\infty}\eta_j\beta_j,\ \beta_j=\int_T\beta(t)\theta_j(t)dt. \quad \text{(N62)}$$

Similar to multivariate CCA where we consider the correlation between linear combinations of variables in two sets, the functional CCA consider extensions of linear combinations of the random variables to the functional space. Using equations (N48), (N61) and (N62) we calculate the covariance:

$$\operatorname{cov}(\int_T \alpha(s)X(s)ds, \int_T \beta(t)Y(t)dt) = \int_T \alpha(s)R_{XY}(s,t)\beta(t)dt\gamma$$
$$= \sum_{i=1}^{\infty}\sum_{j=1}^{\infty}\alpha_i\beta_j\gamma_{ij} \quad \text{(N63)}$$

Similarly, we have

$$\operatorname{var}(\int_T \alpha(s)X(s)ds) = \sum_{i=1}^{\infty}\lambda_i\alpha_i^2$$
$$\operatorname{var}(\int_T \beta(t)Y(t)dt = \sum_{j=1}^{\infty}\mu_j\beta_j^2. \quad \text{(N64)}$$

Therefore, the FCCA can be defined as

$$\max_{\alpha,\beta} \quad \alpha^T\Lambda_{XY}\beta$$
$$\text{s.t.} \quad \alpha^T\Lambda_{XX}\alpha = 1, \beta^T\Lambda_{YY}\beta = 1, \quad \text{(N65)}$$

where

$$\Lambda_{XY} = \begin{bmatrix} \gamma_{11} & \cdots & \gamma_{1q} \\ \vdots & \vdots & \vdots \\ \gamma_{p1} & \cdots & \gamma_{pq} \end{bmatrix}, \Lambda_{XX} = \begin{bmatrix} \lambda_1 & \cdots & 0 \\ \vdots & \vdots & \vdots \\ 0 & \cdots & \lambda_p \end{bmatrix}, \Lambda_{YY} = \begin{bmatrix} \mu_1 & \cdots & 0 \\ \vdots & \vdots & \vdots \\ 0 & \cdots & \mu_q \end{bmatrix}, \alpha = \begin{bmatrix} \alpha_1 \\ \vdots \\ \alpha_p \end{bmatrix}, \beta = \begin{bmatrix} \beta_1 \\ \vdots \\ \beta_q \end{bmatrix}.$$

Comparing equation (N63) with equation (N56) gives

$$\operatorname{cov}(\int_T \alpha(s)X(s)ds, \int_T \beta(t)Y(t)dt) = \operatorname{cov}(\Psi_X(\alpha), \Psi_Y(\beta)).$$

This shows that the formulation of FCCA in equation (N65) is equivalent to the formulation of the FCCA in the L_X^2 , L_Y^2 and $\mathcal{H}(R_X), \mathcal{H}(R_Y)$ as expressed in equation (N58). Similar to CCA, R^2 in FCCA can be defined in terms of functional principal component scores:

$$R^2 = \Lambda_{YY}^{-1/2}\Lambda_{YX}\Lambda_{XX}^{-1}\Lambda_{XY}\Lambda_{YY}^{-1/2}.$$

After some algebra, we obtain the association measure r in equation (11) :

$$r = \mathrm{Tr}(R^2) = \sum_{i=1}^{p} \sum_{j=1}^{q} \frac{\gamma_{ij}^2}{\lambda_i \mu_j} . \qquad \text{(N66)}$$

If constraints $\alpha^T \Lambda_{XX} \alpha = 1, \beta^T \Lambda_{YY} \beta = 1$ in equation (N65) is replaced by $\alpha^T \alpha = 1$ and $\beta^T \beta = 1$, i.e., the optimization problem (N65) is reduced to

$$\begin{aligned} \max_{\alpha,\beta} \quad & \alpha^T \Lambda_{XY} \beta \\ \text{s.t.} \quad & \alpha^T \alpha = 1, \beta^T \beta = 1, \end{aligned} \qquad \text{(N67)}$$

then equation (N66) is reduced to

$$r = \mathrm{Tr}(R^2) = \sum_{i=1}^{p} \sum_{j=1}^{q} \gamma_{ij}^2 . \qquad \text{(N68)}$$

Assume that the expansion of $X(t)$ and $Y(t)$ in equation (N46) are truncated by p and q terms, respectively. Define the feature maps:

$\Phi(X_i) = [\xi_{i1},...,\xi_{ip}]^T = \xi_i$ and $\Phi(Y_i) = [\eta_{i1},...,\eta_{iq}]^T = \eta_i$, where ξ_{ik} and η_{il} are the FPC score of $X_i(t)$ and $Y_i(t)$ respectively.

Let

$$\Phi(X) = \begin{bmatrix} \Phi^T(X_1) \\ \vdots \\ \Phi^T(X_m) \end{bmatrix} = \xi = \begin{bmatrix} \xi_{11} & \cdots & \xi_{1p} \\ \vdots & \vdots & \vdots \\ \xi_{m1} & \cdots & \xi_{mp} \end{bmatrix} \text{ and}$$

$$\Phi(Y) = \begin{bmatrix} \Phi^T(Y_1) \\ \vdots \\ \Phi^T(Y_m) \end{bmatrix} = \eta = \begin{bmatrix} \eta_{11} & \cdots & \eta_{1q} \\ \vdots & \vdots & \vdots \\ \eta_{m1} & \cdots & \eta_{mq} \end{bmatrix}.$$

Define kernel Gram matrices $K_x = \Phi(X)\Phi^T(X)$ and $K_y = \Phi(Y)\Phi^T(Y)$. The dependence measure is

$$D_{YX} = \frac{1}{m^2}\mathrm{Tr}\,(\hat{K}_x\hat{K}_y) = \frac{1}{m^2}\mathrm{Tr}(\xi\xi^{\mathrm{T}}\eta\eta^{\mathrm{T}}) = \frac{1}{m^2}\mathrm{Tr}(\xi^{\mathrm{T}}\eta\eta^{\mathrm{T}}\xi). \qquad \text{(N69)}$$

Note that $\xi^T\eta$ asymptotically converges to $E[\xi^T\eta] = \Lambda_{XY}$. Therefore, the dependence measure D_{YX} based on FPCs asymptotically converges to

$D_{YX} \approx \frac{1}{m^2}\mathrm{Tr}(\Lambda_{XY}\Lambda_{XY}^T) = \frac{1}{m^2}\sum_{i=1}^{p}\sum_{j=1}^{q}\gamma_{ij}^2 = \frac{1}{m^2}r$. In other words, the dependence measure based on the FPCs is asymptotically equal to the association measure of the FCCA.

Supplemental Note C

Impact of Derive Traits on Association Tests

Association Tests of Rare Variants

To investigate whether derived traits BMI, WHR, FEV1.FVC_Ratio and Total_Lean_Mass can cause spurious association or not, we removed these derived traits from the original data reanalyze the association of rare variants. The results were summarized in Table SN1 and Fig SN1. We observed that the number of significant findings after removing derived traits was slightly reduced and the P-values were slightly increased.

Association Tests of Common Variants

The same conclusion can be made for common variants. The Manhattan plots showing genome-wide p-values of association of genes consisting of only common variants with the 46 traits calculated using QRFCCA before and after removing derived traits were presented in Fig 11 and Fig SN2, respectively.

Impact of Age on Association Tests

To investigate the impact of covariates on the association of the proposed test, we conducted real data analysis adjusted for age. The results were summarized in Table SN2. The P-values of QFCCA test for association of common and rare variants with 46 traits were listed in Table SN3. The results showed that age was a confounding factor. After age adjusted, the number of significantly associated genes was slightly reduced. We also observe that the impact of age on association of rare variants is larger than that on common variants.

Impact of Homogeneity on Association Tests

To show how the genes were associated with the homogeneous groups of traits, we presented Tables SN4 and SN5 that summarized the number of genes (rare variants only) significantly associated with the kidney group and glycemic group traits with and without PC adjustments, respectively. We observed that the impact of population structure on the QRFCCA was small, but on the MSKAT was large.

Confirmation of Literature

A large proportion of association tests can be confirmed by publication in the literature. Table SN6 summarized the proportions of identified genes in 5 kidney traits, 2 glycemic traits and 7 lipid traits which have been published in the literature.

Supplemental References

1. Eubank RL, Hsing T. Canonical correlation for stochastic processes. Stochastic Processes and their Applications. 2008;118(9):1634-1661.
2. Gretton A. Introduction to RKHS, and some simple kernel algorithms. 2015. Available: http://www.gatsby.ucl.ac.uk/~gretton/coursefiles/lecture4_introToRKHS.pdf.
3. Gretton A. Notes on mean embeddings and covariance operators. 2015. Available: http://www.gatsby.ucl.ac.uk/~gretton/coursefiles/lecture5_covarianceOperator.pdf.
4. Gretton A, Fukumizu K, Teo CH, Song L, Scholkopf B, Smola AJ. A Kernel Statistical Test of Independence. In: Platt JC, Koller D, Singer Y, Roweis ST, editors. Advances in Neural Information Processing Systems 20 (NIPS 2007); Vancouver, Canada2007. p. 1-8.
5. Broadaway KA, Cutler DJ, Duncan R, Moore JL, Ware EB, Jhun MA, et al. A Statistical Approach for Testing Cross-Phenotype Effects of Rare Variants. Am J Hum Genet. 2016;98(3):525-540.
6. Kuss M, Graepel T. The Geometry of Kernel Canonical Correlation Analysis. Max Planck Institute for Biological Cybernetics, Tübingen, Germany, Inference E; 2003.
7. Bosq D. Linear processes in function spaces : theory and applications. New York: Springer; 2000. xiii, 283 p. p.
8. Kupresanin A, Shin H, King D, Eubank RL. An RKHS framework for functional data analysis. Journal of Statistical Planning and Inference. 2010;140(12):3627-3637.
9. Henderson D, Plaschko P. Stochastic differential equations in science and engineering. New Jersey: World Scientific Pub.; 2006. 215 p. p.

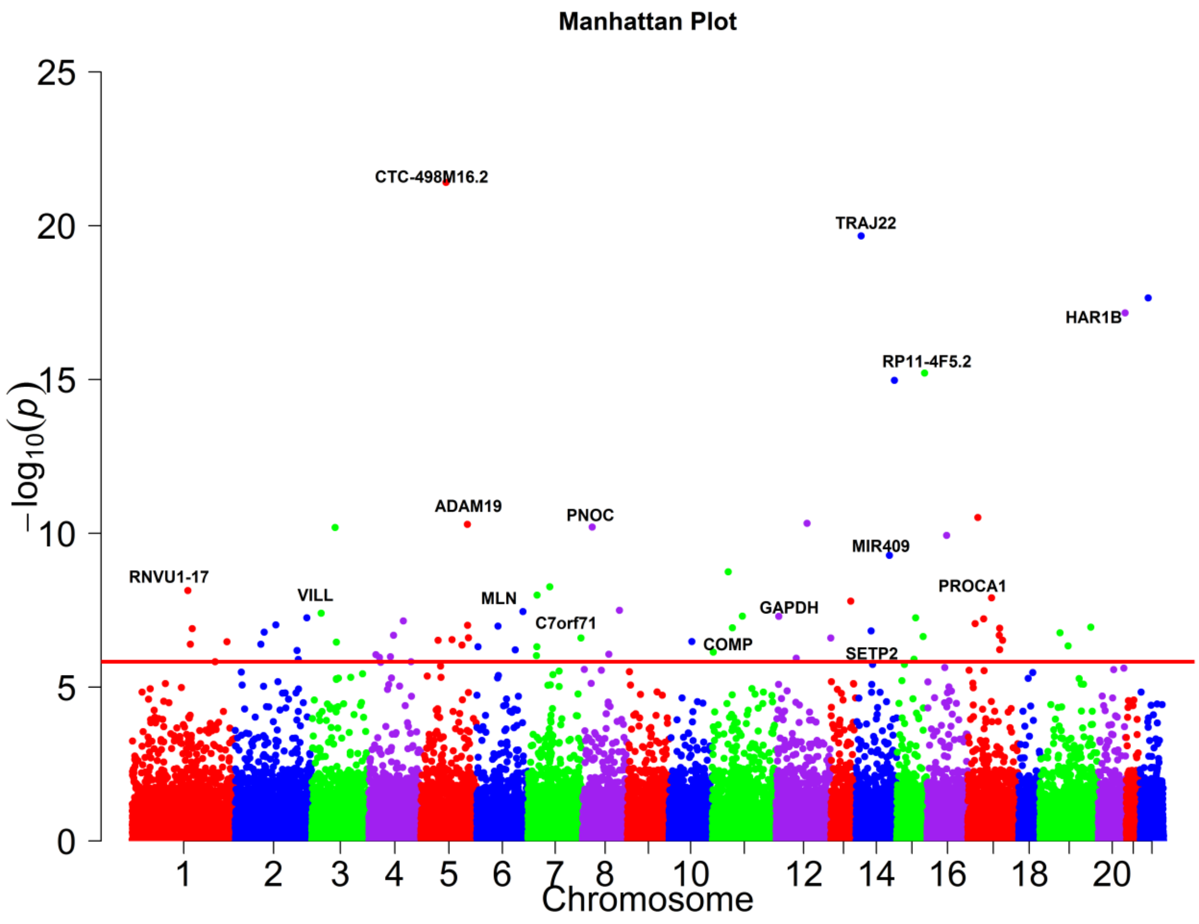

Fig SN1. Manhattan plot showing the genome-wide P values of association of the genes consisting of only rare variants with the 42 traits calculated using QRFCCA (removing BMI, WHR, FEV1.FVC_Ratio and Total_Lean_Mass from the original 46 trains). The axis x represented the chromosomal positions of 33,746 genes and axis y showed their $-\log_{10} P$-values. The horizontal red line denotes the thresholds of $P = 1.48 \times 10^{-6}$ for genome-wide significance after Bonferroni correction.

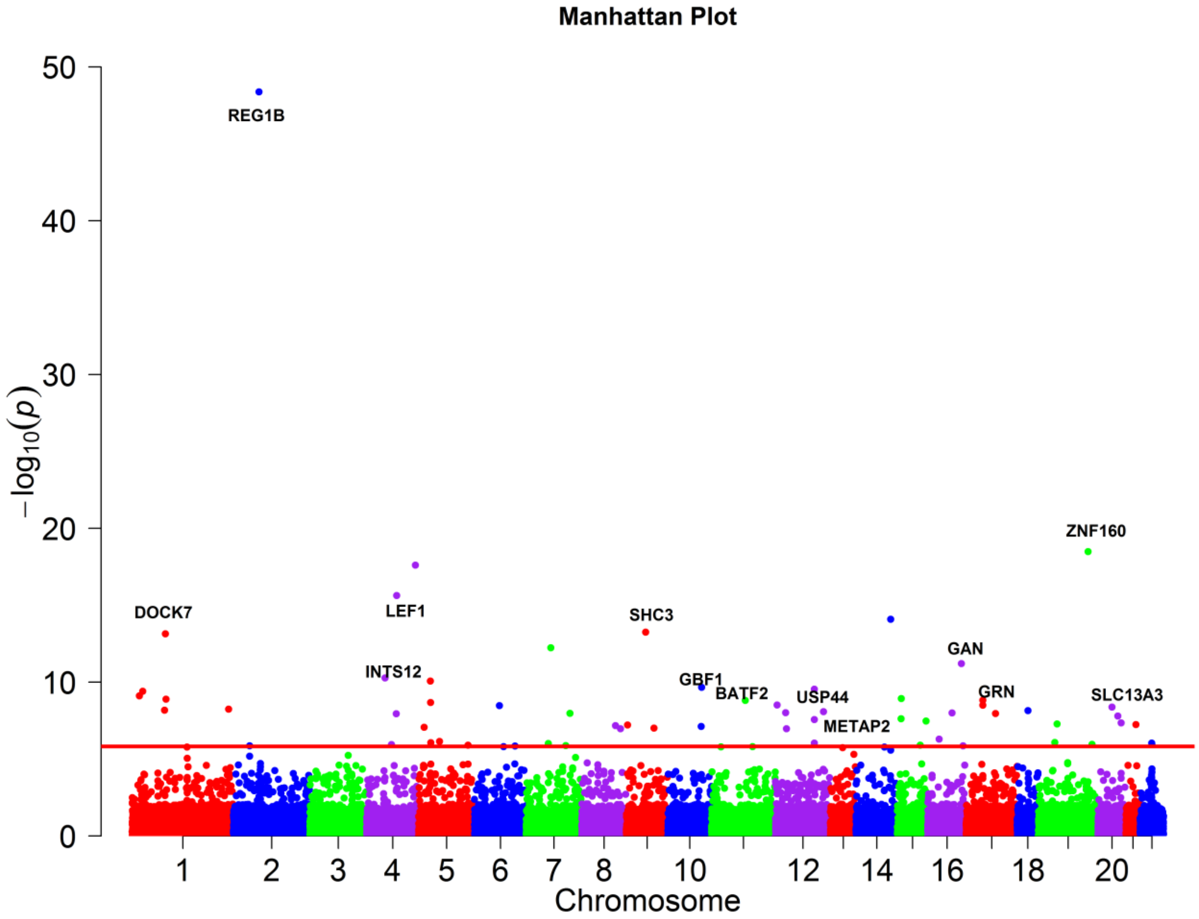

Fig SN2. Manhattan plot showing the genome-wide P values of association of the genes consisting of only common variants with the 46 traits calculated using QRFCCA (removing BMI, WHR, FEV1.FVC_Ratio and Total_Lean_Mass from the original 46 trains). The axis x represented the chromosomal positions of 33,166 genes and axis y showed their $-\log_{10} P$-values. The horizontal red line denotes the thresholds of $P = 1.51 \times 10^{-6}$ for genome-wide significance after Bonferroni correction.

Table SN1. The number of significant findings in the original and trait removed datasets.

		Body	Lung	Total
Common	Original	0	3	67
	Removed	0	3	64
Rare	Original	0	2	80
	Removed	0	3	71

Table SN2. The number of the genes significantly associated with 46 traits with age adjusted and no age adjusted.

	No adjusted	Age Adjusted
Common	67	63
Rare	79	68

Table SN3. The list of p-value for the significant genes before and after age adjustment for both common and rare variants							
Rare Variants				Common Variants			
Gene	Chromosome	QRFCCA	QRFCCA with A	Gene	Chromosome	QRFCCA	QRFCCA with Age A
CTC-498M16.2	5	5.74E-22	1.80E-21	REG1B	2	1.65E-116	4.86E-48
TRAJ22	14	2.16E-20	2.18E-20	RP11-665C14.1	4	1.38E-93	2.18E-18
AP000351.10	22	2.09E-18	1.88E-18	ZNF160	19	1.98E-91	2.74E-18
HAR1B	20	7.81E-18	5.58E-18	LEF1	4	7.44E-83	4.16E-16
IGHVII-20-1	14	7.49E-16	3.82E-16	DYNC1H1	14	3.46E-58	9.50E-15
RP11-4F5.2	15	9.94E-16	3.45E-15	DOCK7	1	4.42E-51	6.12E-13
RNVU1-17	1	3.90E-13	7.16E-09	SHC3	9	7.56E-42	1.05E-14
PNOC	8	1.63E-12	3.37E-10	Y_RNA	7	1.89E-36	9.18E-13
COTL1P1	17	8.71E-12	1.16E-10	CTD-2122P11.1	5	1.62E-33	1.37E-10
LINC00273	16	4.41E-11	1.11E-10	GBF1	10	6.30E-28	1.84E-09
snoU13	12	4.95E-11	2.86E-11	RP1-8B22.1	1	1.75E-27	2.75E-09
ADAM19	5	6.07E-11	4.60E-11	VPS13D	1	2.62E-26	1.26E-09
CTD-2026G6.2	3	1.91E-10	2.78E-11	RP11-68I3.2	17	3.20E-24	1.31E-09
MIR409	14	2.77E-10	6.17E-10	SLC13A3	20	8.51E-24	2.00E-08
RP1-276E15.1	11	3.13E-10	3.66E-09	RP11-167N24.3	12	4.33E-23	2.44E-10
HMGN1P6	2	4.46E-10	1.02E-06	UBA6	4	5.26E-22	2.85E-10
HOXA7	7	2.18E-09	1.41E-08	GAN	16	1.49E-21	2.91E-12
RNA5SP99	2	2.41E-09	2.88E-06	RP4-794H19.2	1	4.07E-21	6.64E-09
AC021660.1	3	2.69E-09	0.000670466	RP11-142I20.1	18	5.29E-21	6.84E-09
RP11-561N12.1	7	3.12E-09	1.32E-09	METAP2	12	2.62E-20	8.94E-07
FBXL5	4	4.53E-09	1.08E-06	SLCO1C1	12	5.89E-20	9.12E-09
PPIAP23	13	5.40E-09	2.90E-08	AC105443.2	7	2.47E-17	1.18E-08
HOXB2	17	7.00E-09	2.47111E-07	GRN	17	1.63E-16	1.29E-08
RP11-170N16.1	4	7.09E-09	5.25E-08	INTS12	4	1.72E-16	1.29E-08
AC008694.3	5	1.39E-08	9.89E-08	RP11-323I15.5	15	9.29E-16	1.48E-09
VILL	3	1.69E-08	9.42E-09	UBE2U	1	2.88E-15	1.66E-09
KRT27	17	2.04E-08	1.92E-08	BATF2	11	5.90E-15	2.19E-08
RNU6-1243P	11	2.28E-08	3.99E-08	C5orf51	5	1.02E-14	2.29E-09
RP11-6N17.10	17	2.49E-08	1.48E-05	RP11-814P5.1	15	1.28E-14	3.31E-08
AL358134.2	6	2.83E-08	7.07E-08	USP44	12	3.49E-14	4.34E-08

GAPDH	12	2.96E-08	9.33E-08	C12orf5	12	4.91E-14	2.44E-09
DERL1	8	3.45E-08	6.44E-08	SSH2	17	5.59E-14	4.44E-09
RP1-102E24.9	12	4.32E-08	4.18E-06	PHIP	6	2.96E-13	6.48E-09
PPBP	4	4.79E-08	9.68E-07	ARRDC4	15	1.73E-12	5.64E-08
LINC00443	13	5.08E-08	0.117002591	RP3-477O4.16	20	4.33E-12	7.07E-09
RP11-10O17.1	15	5.19E-08	1.11E-08	FAM210B	20	5.59E-11	6.09E-08
PROCA1	17	5.62E-08	3.68E-07	AF001550.7	16	6.53E-11	6.32E-07
CHMP2B	3	6.03E-08	3.74348E-07	COLGALT1	19	9.53E-11	3.35E-07
IGKV2-4	2	6.33E-08	1.20197E-07	IGSF5	21	1.24E-10	9.98E-08
AC017104.1	2	8.15E-08	1.13E-08	GPR137B	1	3.54E-10	6.42E-09
RNU6-980P	19	8.16E-08	3.80E-07	RCL1	9	4.88E-10	5.05E-07
AC016691.2	2	8.89E-08	3.26E-07	OXR1	8	1.77E-09	8.09E-08
RNA5SP19	1	9.03E-08	7.30E-05	CTD-2503O16.4	5	1.48E-08	5.22E-07
snoU13	6	9.16E-08	6.58117E-07	C10orf76	10	1.89E-08	6.16E-08
MLN	6	9.47E-08	4.21E-08	SRRM4	12	2.26E-08	1.40E-08
RP11-55L4.2	17	1.03E-07	9.94E-09	RASAL3	19	5.31E-08	6.93E-07
CTC-505O3.2	5	1.22E-07	1.38E-06	MTHFD2P6	5	5.65E-08	1.18E-06
ISCA1P1	5	1.23E-07	6.06E-07	UQCRBP3	5	7.58E-08	8.72E-08
JUN	1	1.25E-07	7.00E-06	SEC14L3	22	7.90E-08	9.10E-07
AC140061.12	12	1.41E-07	2.23E-06	UGCG	9	7.98E-08	9.34E-08
RP11-697E2.10	15	1.77E-07	8.94E-08	ZNF680	7	1.23E-07	9.47E-07
RNU6-717P	12	1.91E-07	1.15E-06	GPR56	16	1.26E-07	9.53E-08
RP11-98D18.17	1	2.36E-07	6.03E-07	CCDC26	8	1.97E-07	9.64E-06
PPIAP13	10	2.43E-07	3.20E-07	SOX5	12	2.54E-07	1.02E-07
SETP2	14	2.67E-07	7.98E-08	AC009892.10	19	3.17E-07	1.04E-06
AC008691.1	5	3.15E-07	7.52E-06	HPSE	4	3.21E-07	1.04E-06
RP1-40E16.2	6	3.38E-07	5.68E-07	SLC35E4	22	5.08E-07	1.06E-06
RP11-79P5.9	5	3.54E-07	3.42E-06	CTC-575D19.1	5	6.25E-07	1.38E-06
RN7SL699P	17	3.69E-07	4.83E-07	EFTUD1	15	7.87E-07	1.52E-06
OR4A44P	11	3.94E-07	1.34E-07	RP4-742N3.1	7	9.42E-07	1.43E-06
C7orf71	7	4.80E-07	3.05E-07	RP11-568J23.7	16	9.69E-07	1.44E-06
COMP	19	4.83E-07	2.66E-05	AC138655.1	2	1.01E-06	1.31E-06
PCDHB10	5	4.97E-07	8.83E-07	EYA4	6	1.05E-06	1.51E-06

HOXB1	17	5.27E-07	3.31E-05	P2RY6	11	1.11E-06	1.39E-06
RN7SKP280	7	5.39E-07	4.63E-07	BACH2	6	1.24E-06	1.40E-05
SEC31A	4	5.51E-07	1.29E-06	MSH6	2	1.42E-06	4.49E-06
RP11-363E6.4	8	6.56E-07	2.19E-07	CTC-497E21.5	11	1.49E-06	1.70E-06
SHISA4	1	6.84E-07	3.05E-06				
DAZAP2P1	2	7.97E-07	7.71E-07				
PGLYRP3	1	8.97E-07	7.28E-08				
RP11-138H8.4	15	1.09E-06	2.47E-06				
RP11-153M7.3	4	1.10E-06	4.36E-07				
EPS8L2	11	1.15E-06	2.59E-07				
HPN	19	1.15E-06	4.34E-05				
AC092685.1	7	1.34E-06	0.083234568				
AC079781.1	7	1.36E-06	5.88E-06				
CTD-2014B16.2	14	1.41E-06	9.87E-05				
SULT1B1	4	1.43E-06	1.10E-05				
RP11-57G22.1	18	1.44E-06	8.89E-06				

djustment

Table SN4. Number of genes with only rare variants significantly associated with 5 traits (Kidney group).

	QRFCCA	FCCA	GAMuT	SCCA	USAT	MANOVA	CCA	PCA	KCCA	MSKAT
No adjusted	78	73	6	0	0	0	0	5	0	17
Adjusted	55	21	1	0	0	0	0	2	0	11
Overlapped	43	21	0	0	0	0	0	2		7
Proportion	78.00%	100%	0					100%		64%

Table SN5. Number of genes with only rare variants significantly associated with traits (Glycaemia group).

	QRFCCA	FCCA	GAMuT	SCCA	USAT	MANOVA	CCA	PCA	KCCA	MSKAT
No adjusted	24	24	8	0	0	0	0	14	0	17
Adjusted	5	4	1	0	0	0	0	3	0	3
Overlapped	4	4	0	0	0	0	0	3	0	1
Proportion	80.00%	100%	0					100%		33%

Table SN6. Proportion of identified genes confirmed in the literature.

Trait		Kidney	Glycaemia	Lipid
Common	Identified	20	11	5
	Confirmed	8	7	3
	Proportion	40%	63.60%	60%
Rare	Identified	78	24	1
	Confirmed	22	7	0
	Proportion	28%	29%	0
