# Supplementary material for: A quadratically regularized functional canonical correlation analysis for identifying the global structure of pleiotropy with NGS data
Source: PLoS Comput Biol. 2017 Oct 17;13(10):e1005788. doi: 10.1371/journal.pcbi.1005788 (PMC5659802; doi:10.1371/journal.pcbi.1005788)
Supplement: S1 Fig — Fig S1–Fig S36. (PDF) [file pcbi.1005788.s002.pdf]

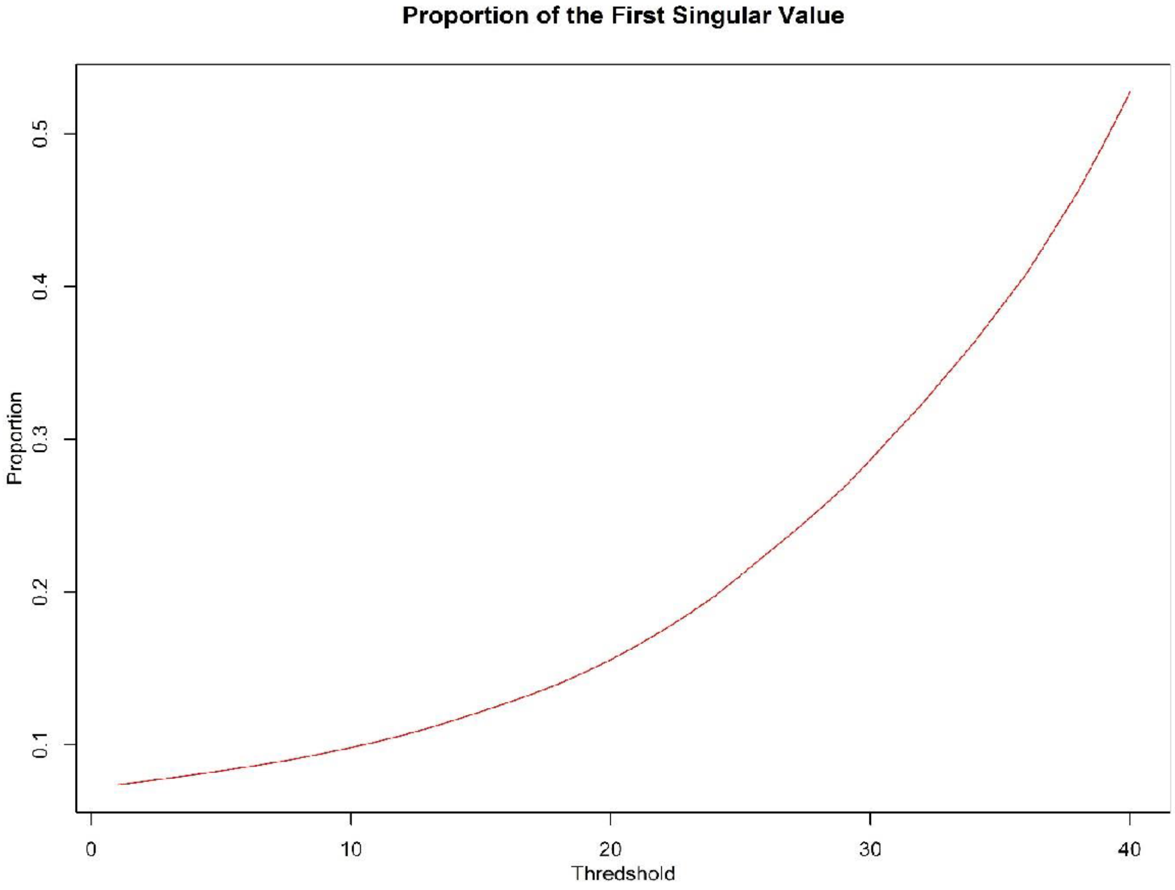


**Fig S1**. Proportion of the first singular value over the total singular values.

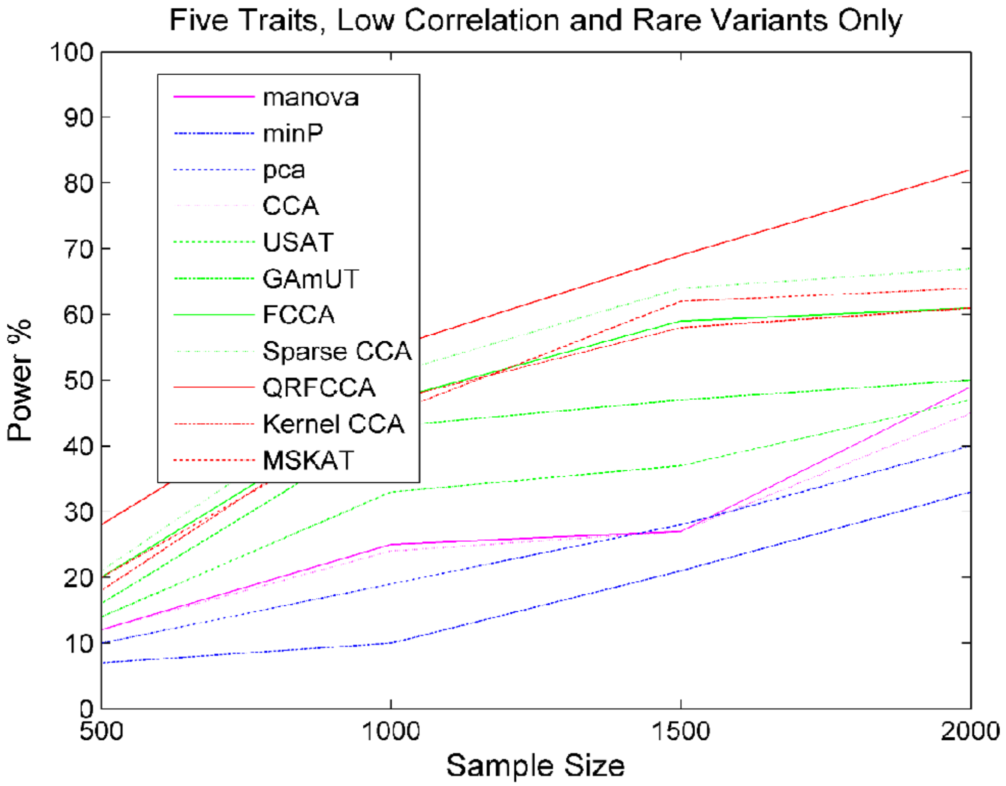


**Fig S2.** The power of curves as a function of sample sizes of 11 statistics for collectively testing the association of all rare variants in the gene with 5 traits with low correlations for scenario 1 at the significance level $\alpha = 0.05$ .

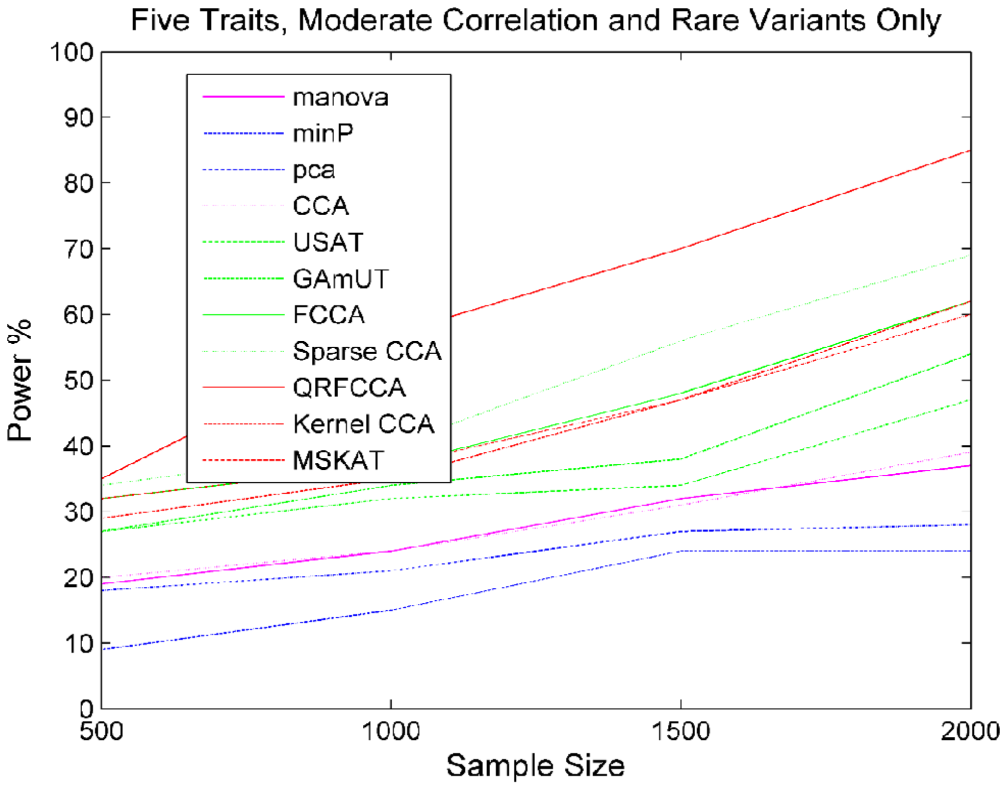


**Fig S3.** The power of curves as a function of sample sizes of 11 statistics for collectively testing the association of all rare variants in the gene with 5 traits with moderate correlations for scenario 1at the significance level $\alpha = 0.05$.

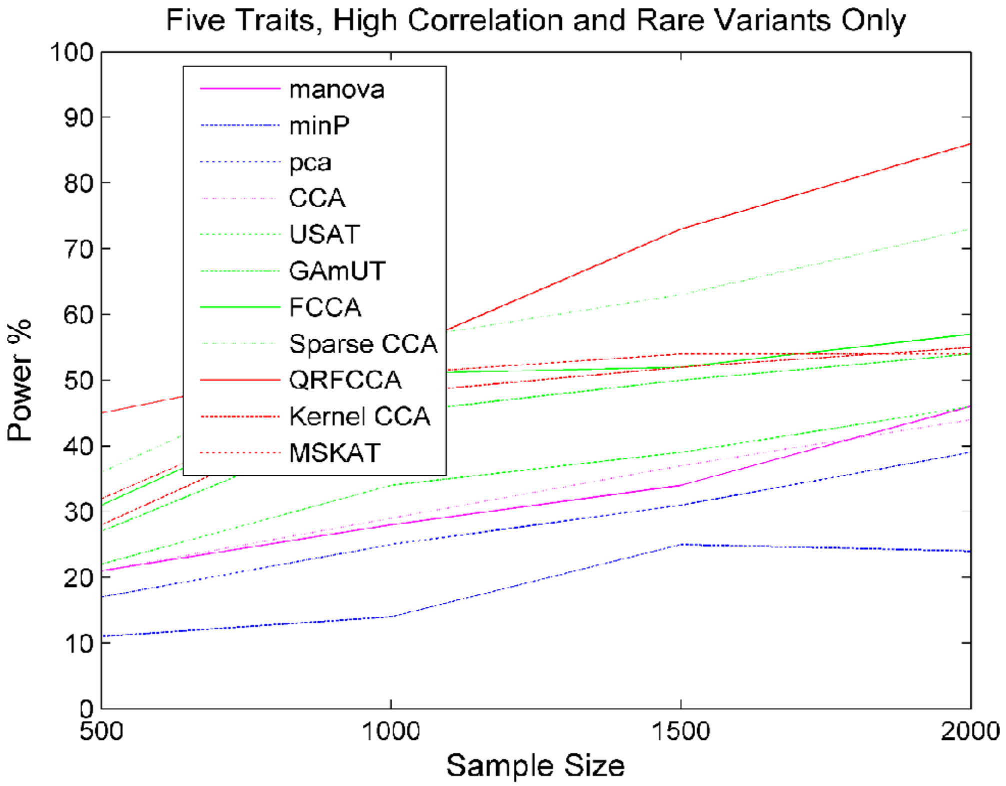


**Fig S4.** The power of curves as a function of sample sizes of 11 statistics for collectively testing the association of all rare variants in the gene with 5 traits with high correlations for scenario 1 at the significance level $\alpha = 0.05$ .

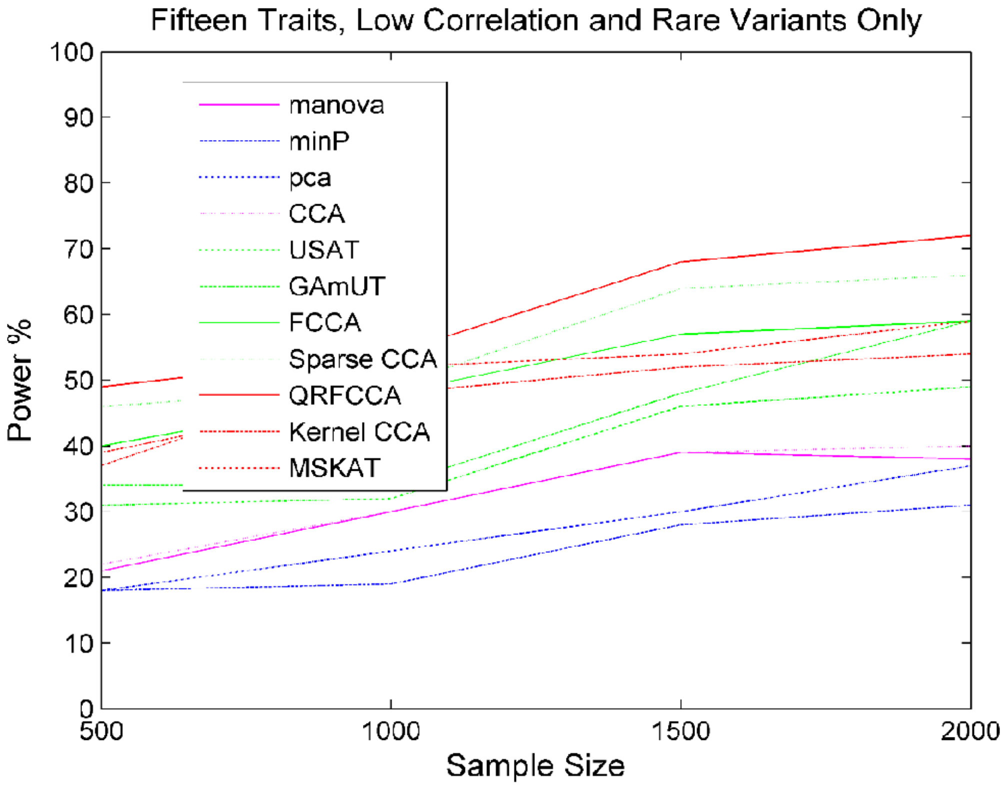


**Fig S5**. The power of curves as a function of sample sizes of 11 statistics for collectively testing the association of all rare variants in the gene with 15 traits with low correlations for scenario 1 at the significance level $\alpha = 0.05$ .

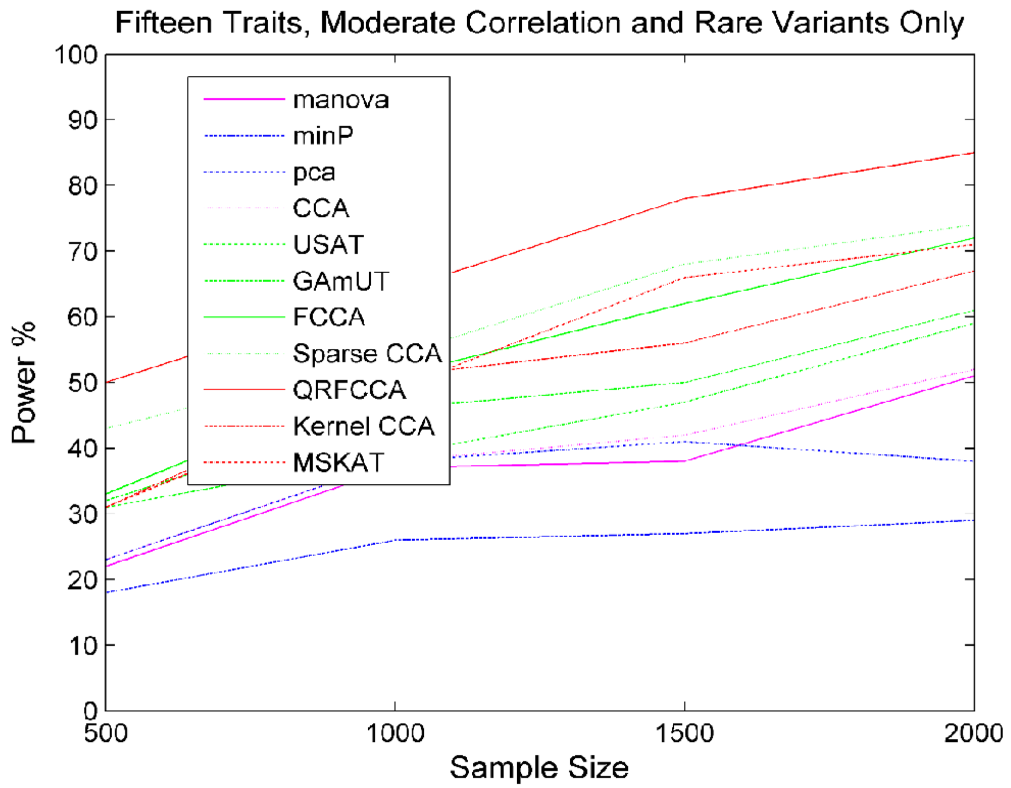


**Fig S6.** The power of curves as a function of sample sizes of 11 statistics for collectively testing the association of all rare variants in the gene with 15 traits with moderate correlations for scenario 1 at the significance level $\alpha = 0.05$ .

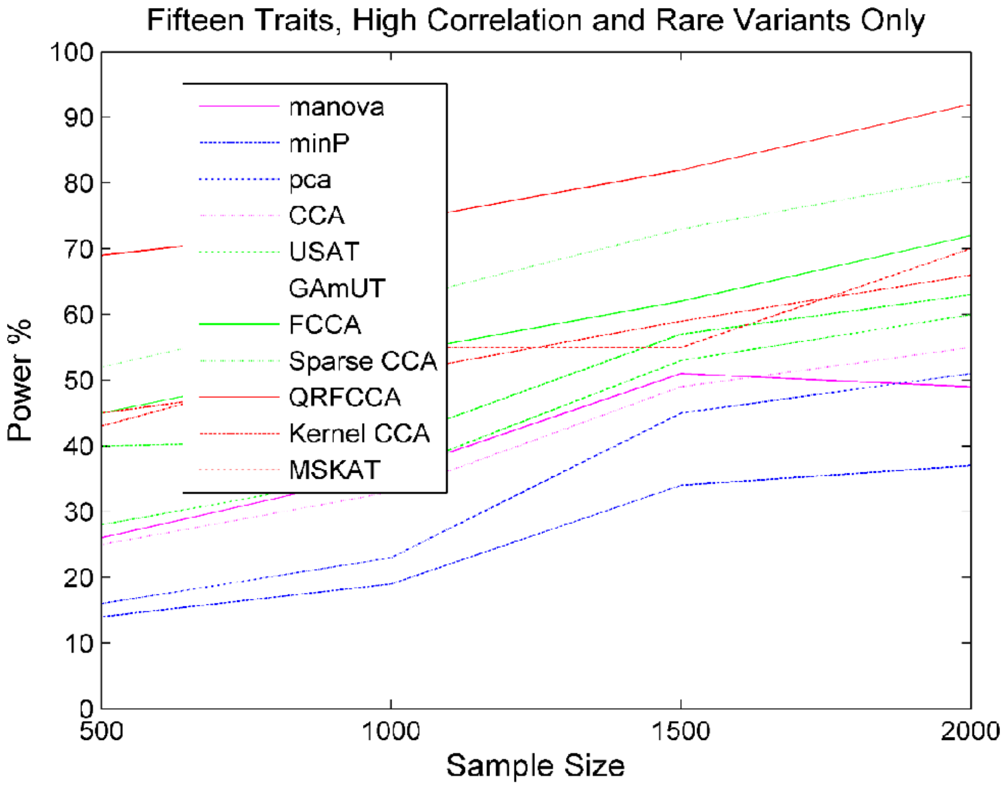


**Fig S7.** The power of curves as a function of sample sizes of 11 statistics for collectively testing the association of all rare variants in the gene with 15 traits with high correlations for the scenario 1 at the significance level $\alpha = 0.05$ .

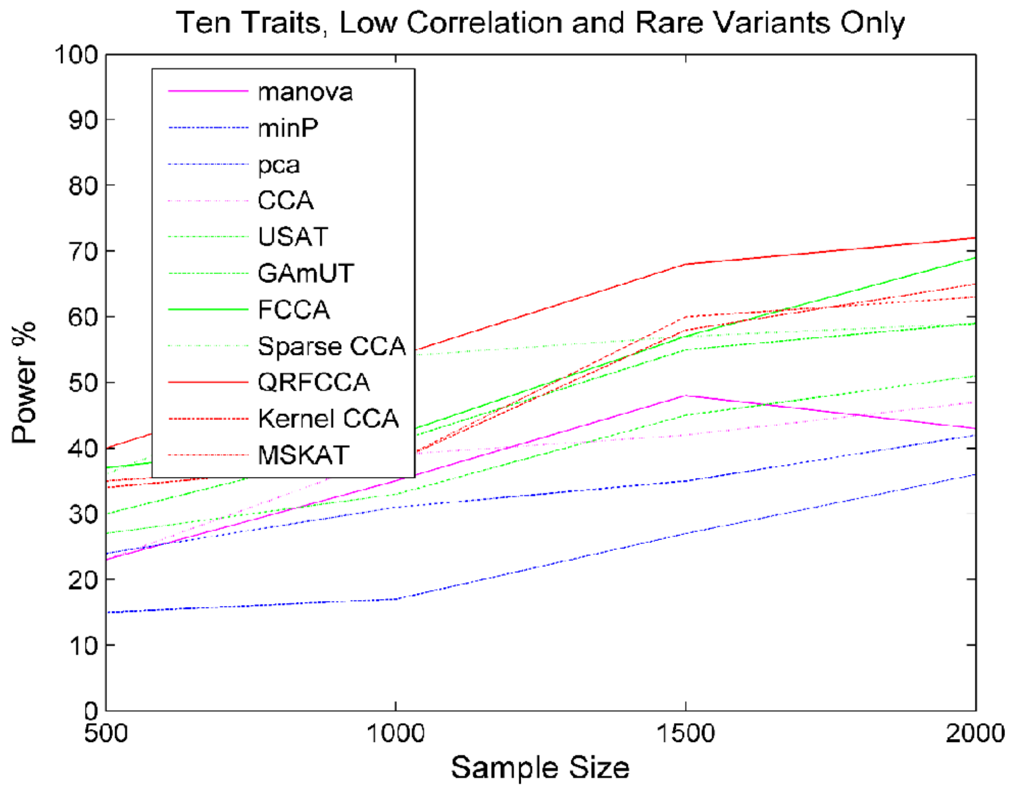


**Fig S8.** The power of curves as a function of sample sizes of 11 statistics for collectively testing the association of all rare variants in the gene with 10 traits with low correlations for the scenario 2 at the significance level $\alpha = 0.05$ .

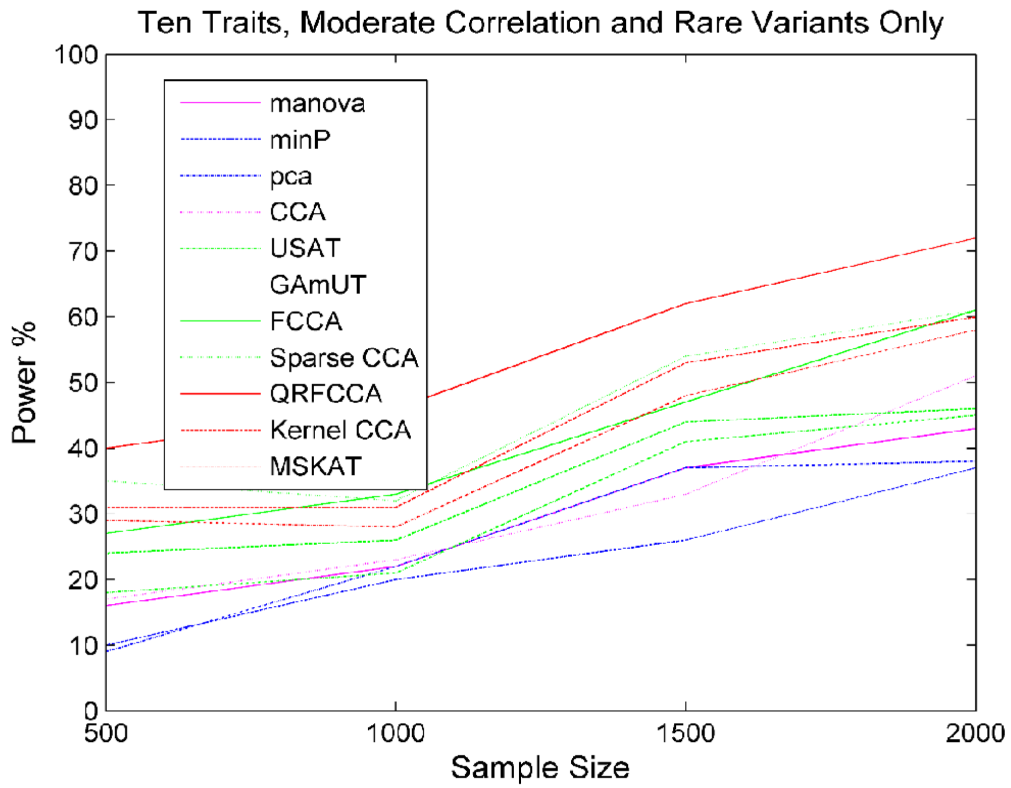


**Fig S9.** The power of curves as a function of sample sizes of 11 statistics for collectively testing the association of all rare variants in the gene with 15 traits with moderate correlations for the scenario 2 at the significance level $\alpha = 0.05$.

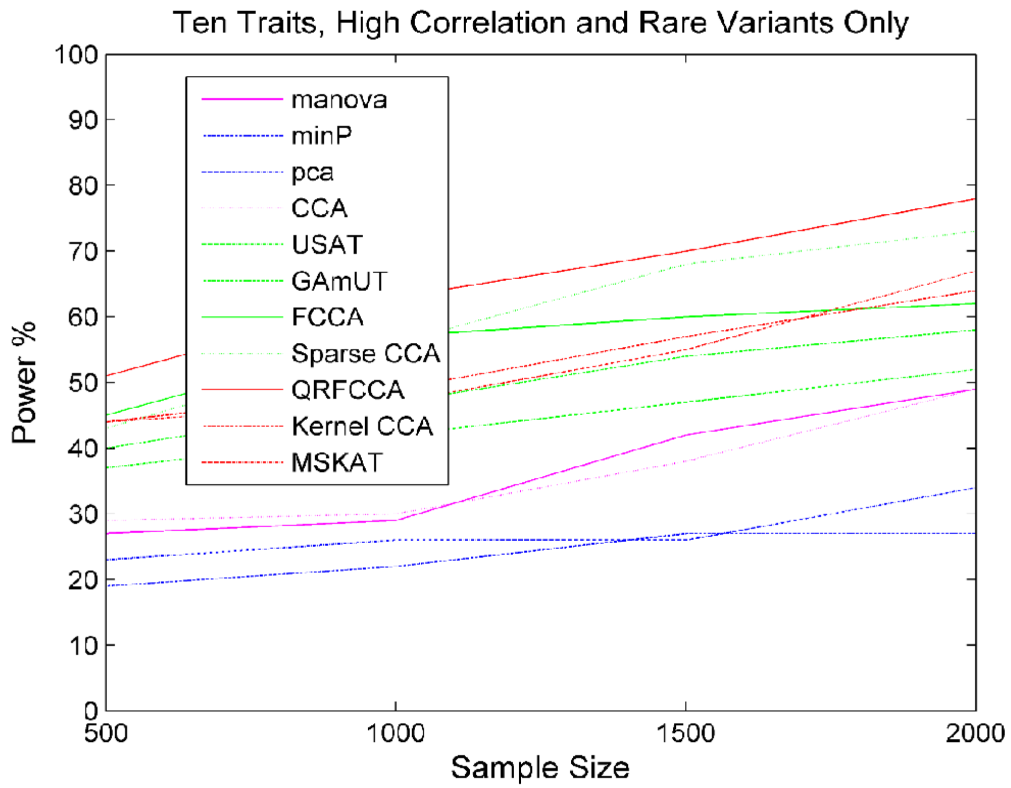


**Fig S10.** The power of curves as a function of sample sizes of 11 statistics for collectively testing the association of all rare variants in the gene with 15 traits with high correlations for the scenario 2 at the significance level $\alpha = 0.05$ .

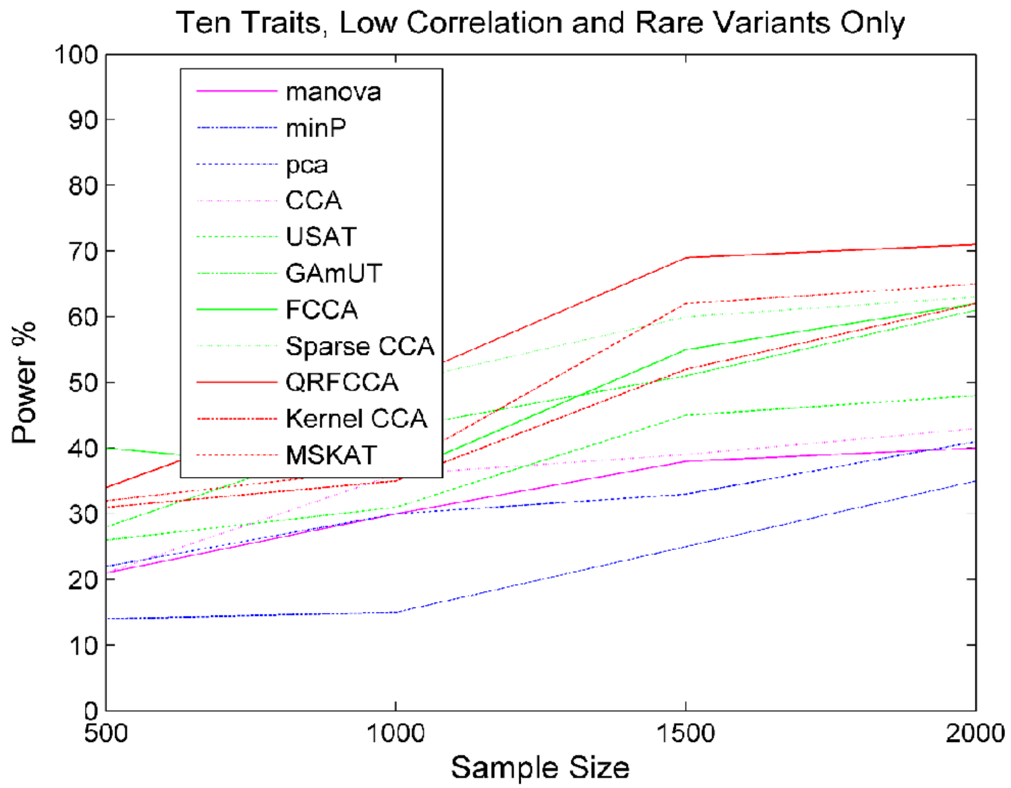


**Fig S11.** The power of curves as a function of sample sizes of 11 statistics for collectively testing the association of all rare variants in the gene with 10 traits with low correlations for the scenario 3 at the significance level $\alpha = 0.05$ .

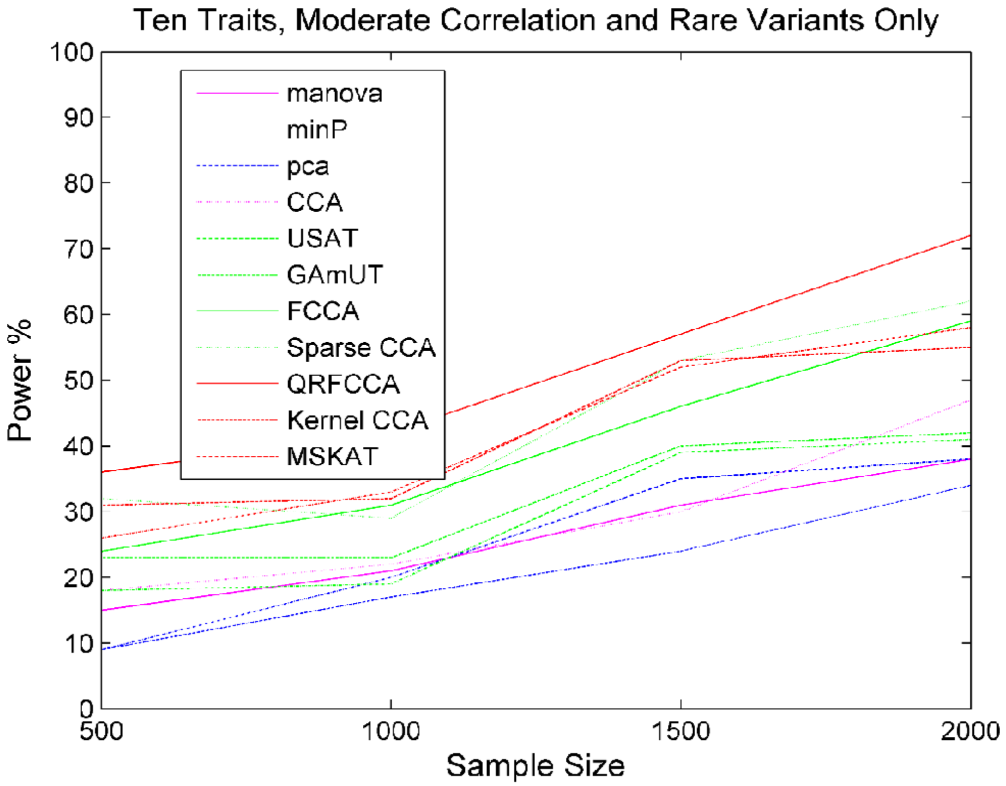


**Fig S12.** The power of curves as a function of sample sizes of 11 statistics for collectively testing the association of all rare variants in the gene with 10 traits with moderate correlations for the scenario 3 at the significance level $\alpha = 0.05$.

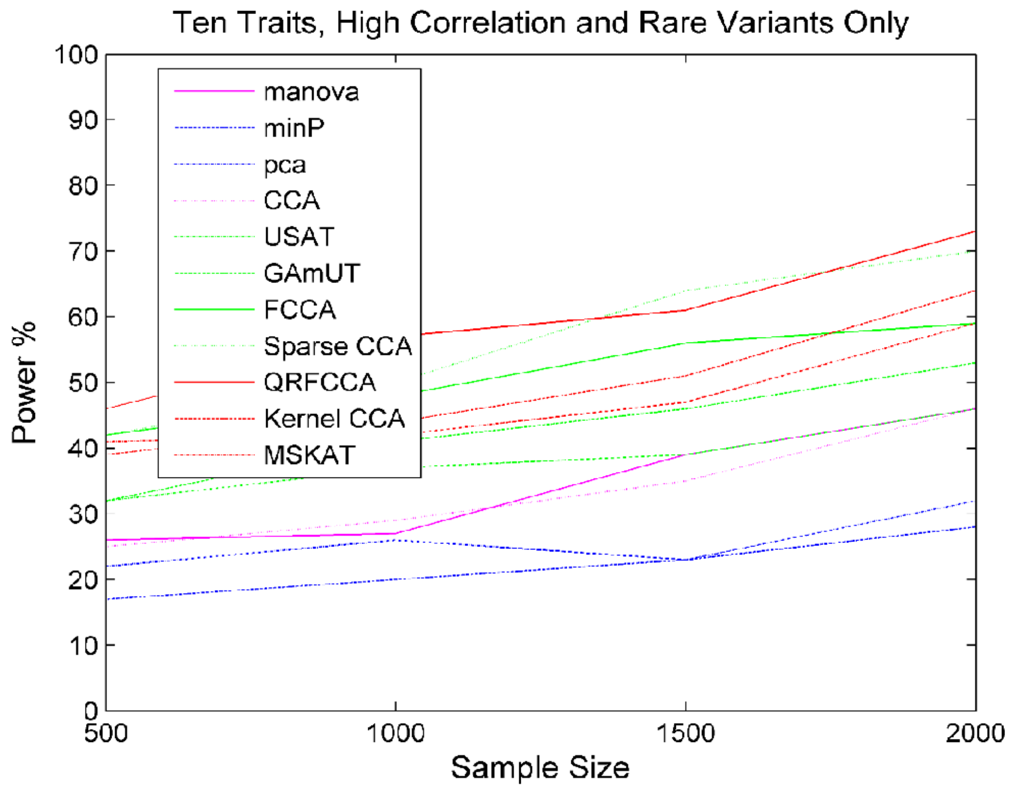


**Fig S13.** The power of curves as a function of sample sizes of 11 statistics for collectively testing the association of all rare variants in the gene with 10 traits with high correlations for the scenario 3 at the significance level $\alpha = 0.05$ .

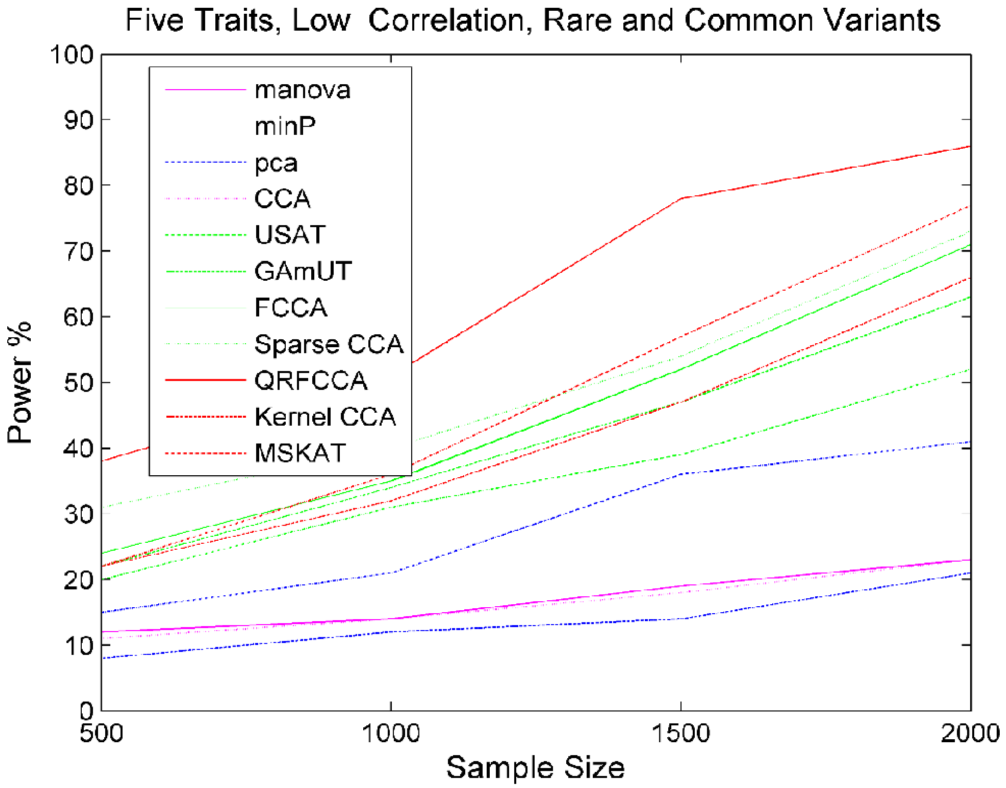


**Fig S14.** The power of curves as a function of sample sizes of 11 statistics for collectively testing the association of the gene including both rare and common variants with 5 traits with low correlations for the scenario 1at the significance level $\alpha = 0.05$.

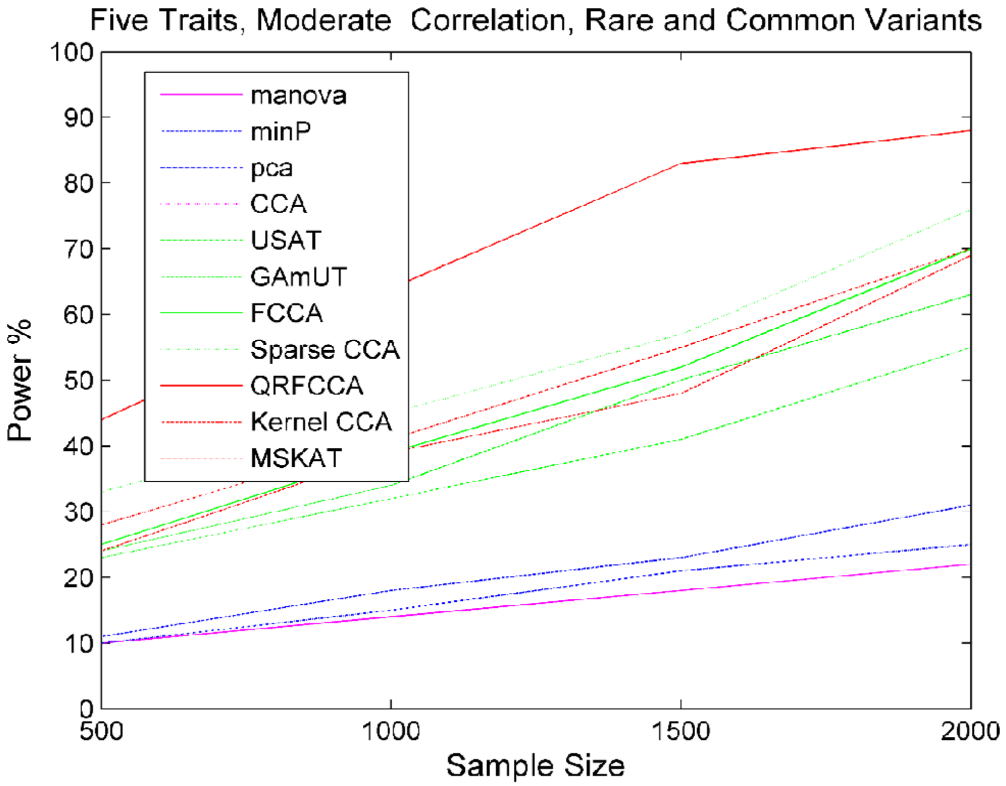


**Fig S15**. The power of curves as a function of sample sizes of 11 statistics for collectively testing the association of the gene including both rare and common variants with 5 traits with moderate correlations for the scenario 1 at the significance level $\alpha = 0.05$ .

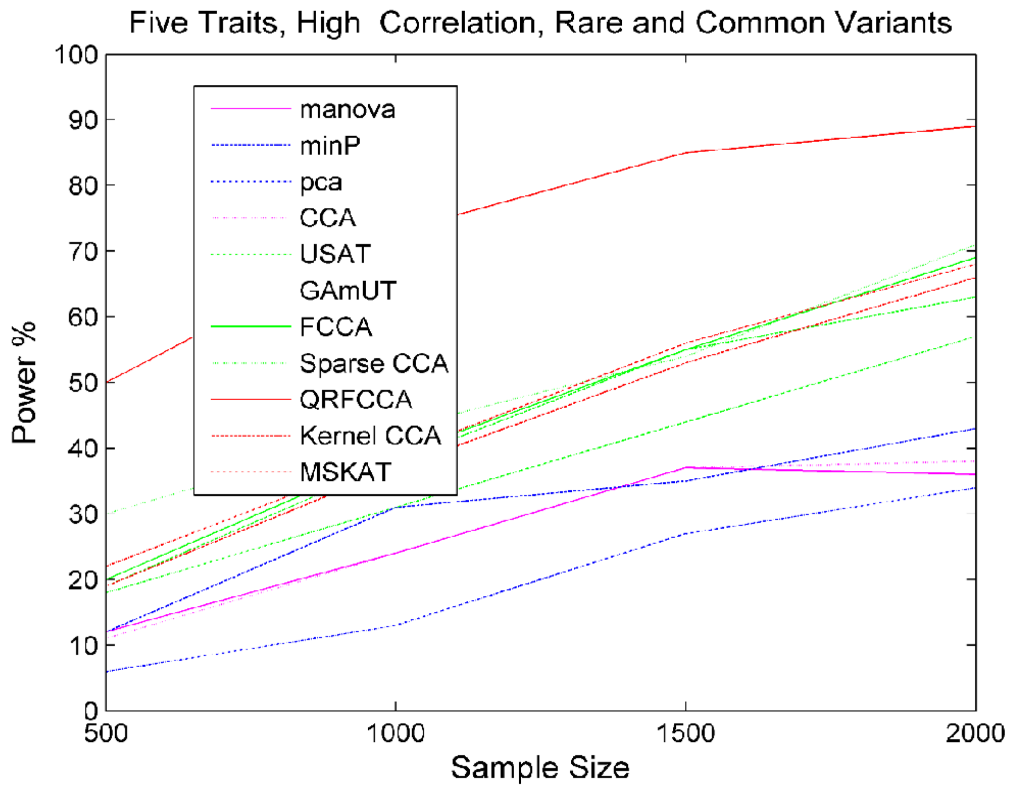


**Fig S16.** The power of curves as a function of sample sizes of 11 statistics for collectively testing the association of the gene including both rare and common variants with 5 traits with high correlations for the scenario 1 at the significance level $\alpha = 0.05$ .

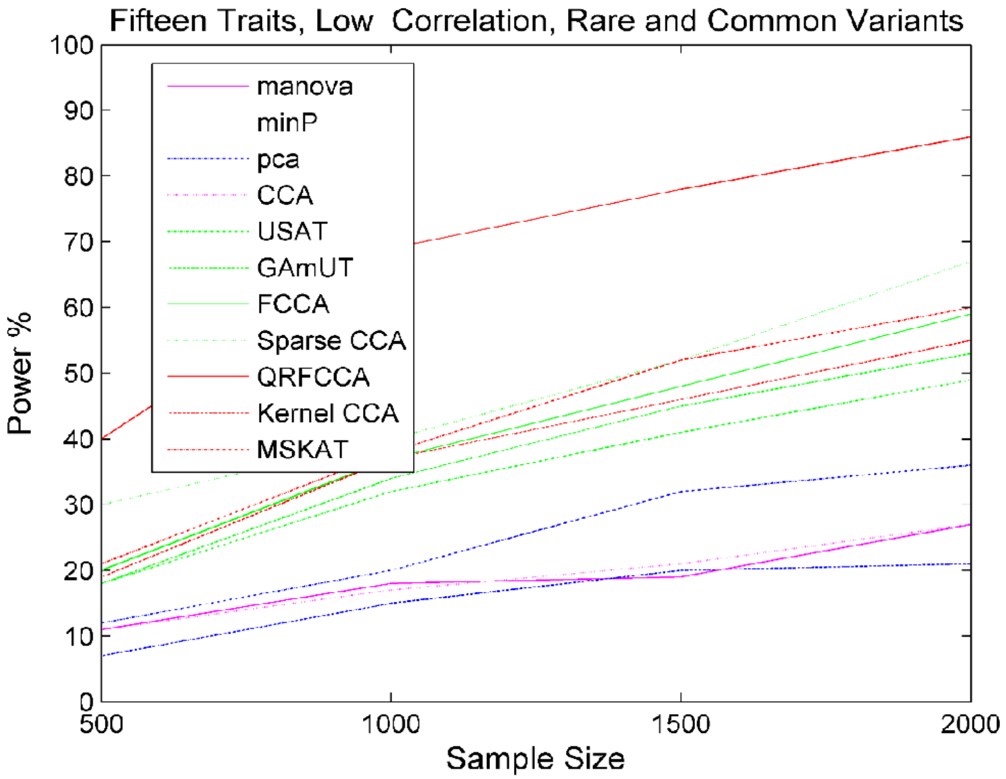


**Fig S17.** The power of curves as a function of sample sizes of 11 statistics for collectively testing the association of the gene including both rare and common variants with 15 traits with low correlations for the scenario 1 at the significance level $\alpha = 0.05$ .

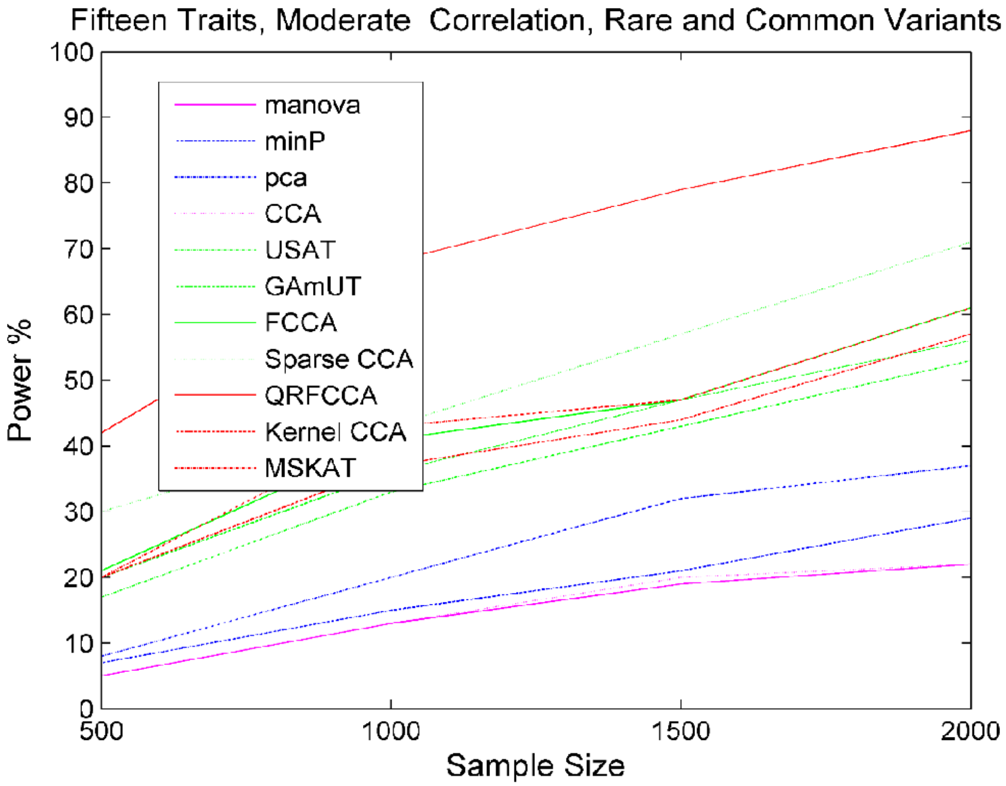


**Fig S18**. The power of curves as a function of sample sizes of 11 statistics for collectively testing the association of the gene including both rare and common variants with 15 traits with moderate correlations for the scenario 1 at the significance level $\alpha = 0.05$ .

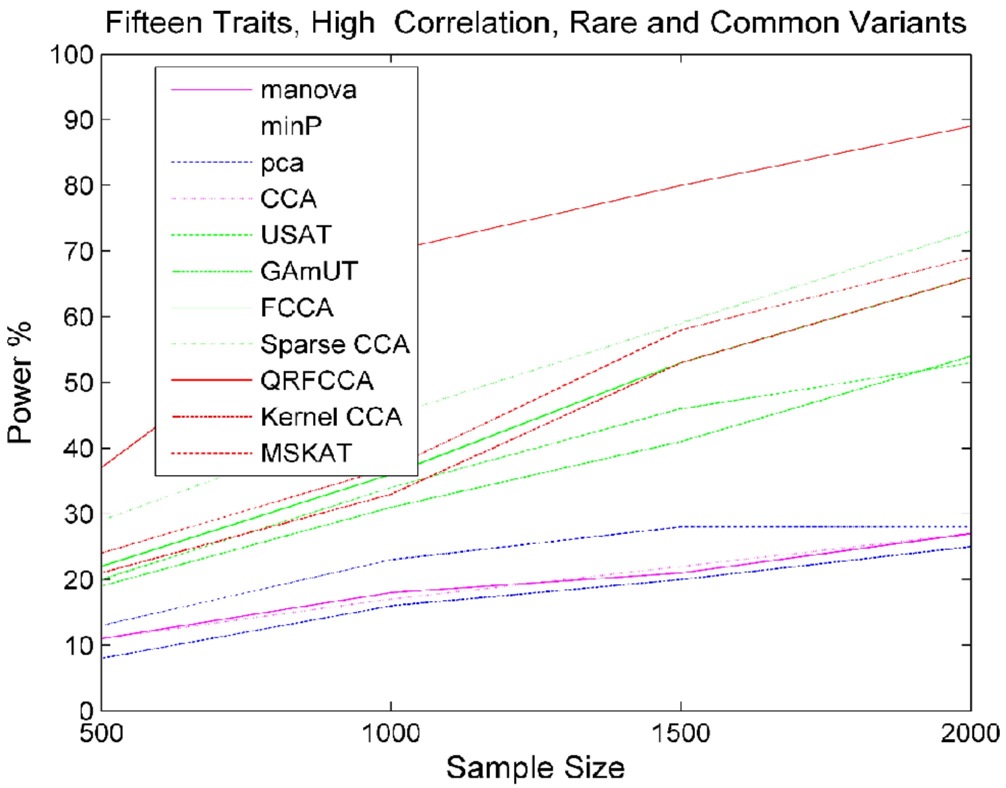


**Fig S19.** The power of curves as a function of sample sizes of 11 statistics for collectively testing the association of the gene including both rare and common variants with 15 traits with high correlations for the scenario 1 at the significance level $\alpha = 0.05$ .

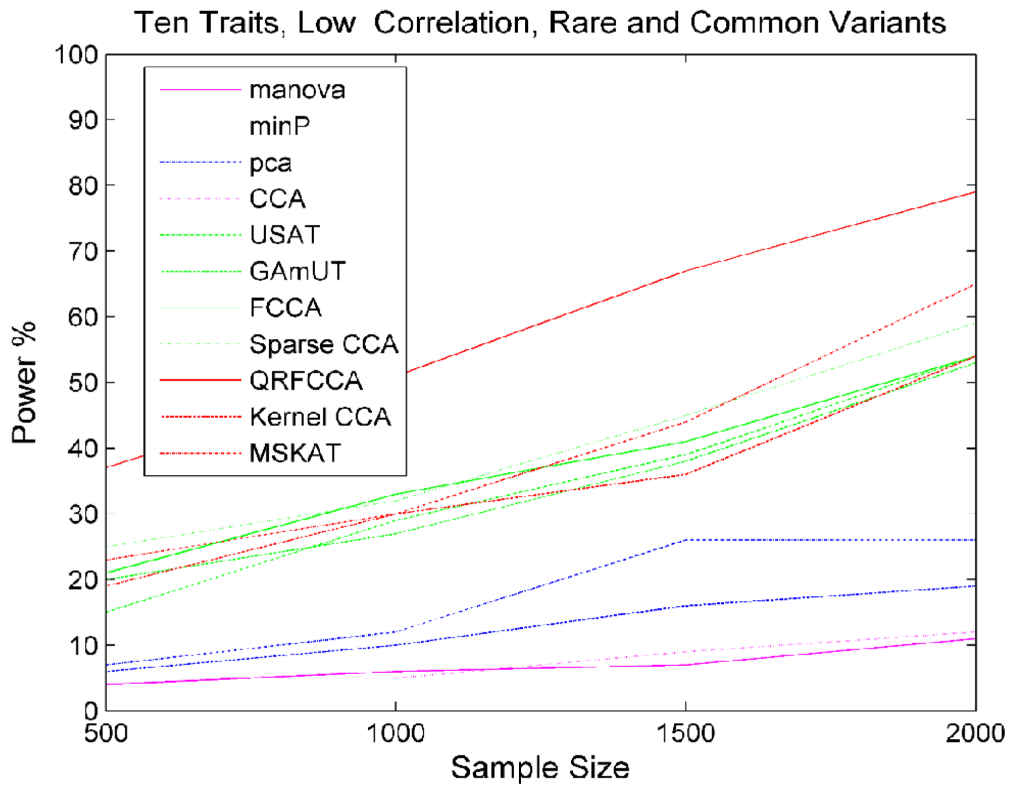


**Fig S20.** The power of curves as a function of sample sizes of 11 statistics for collectively testing the association of the gene including both rare and common variants with 10 traits with low correlations for the scenario 2 at the significance level $\alpha = 0.05$.

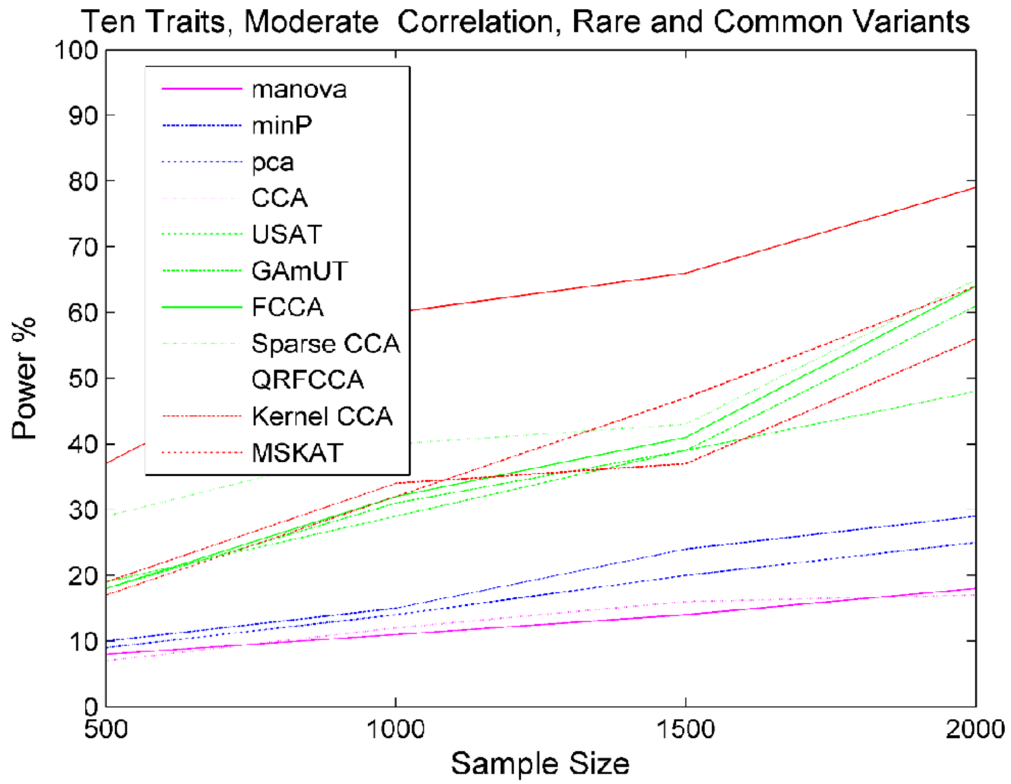


**Fig S21**. The power of curves as a function of sample sizes of 11 statistics for collectively testing the association of the gene including both rare and common variants with 10 traits with moderate correlations for the scenario 2 at the significance level $\alpha = 0.05$ .

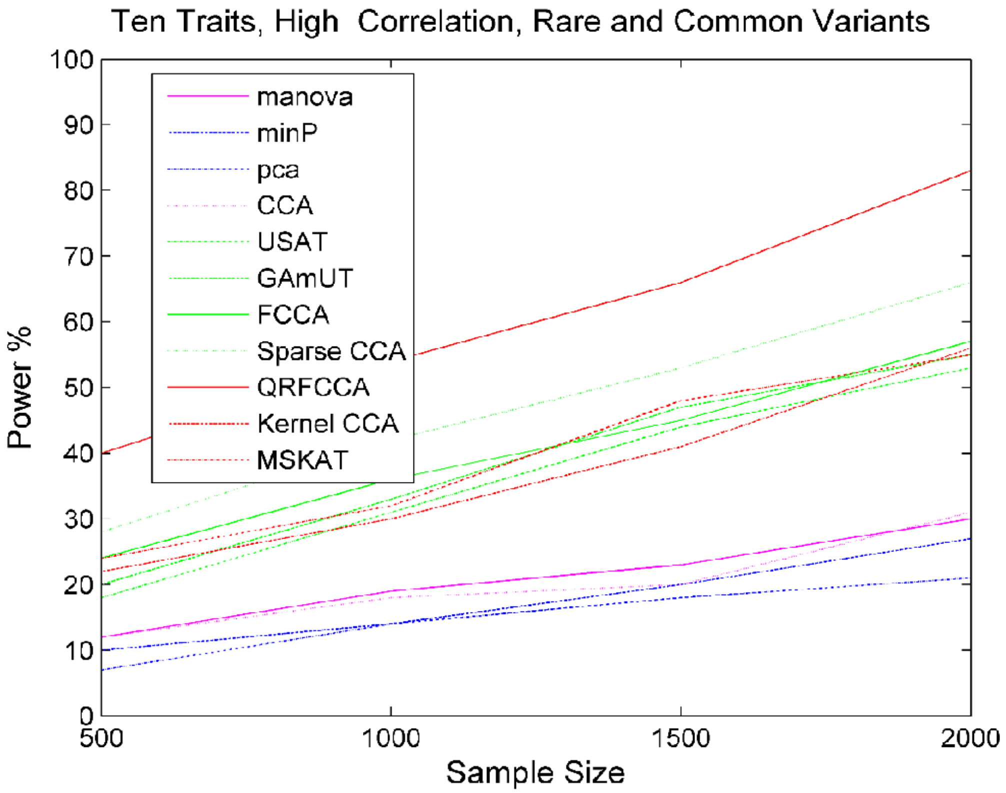


**Fig S22.** The power of curves as a function of sample sizes of 11 statistics for collectively testing the association of the gene including both rare and common variants with 10 traits with high correlations for the scenario 2 at the significance level $\alpha = 0.05$ .

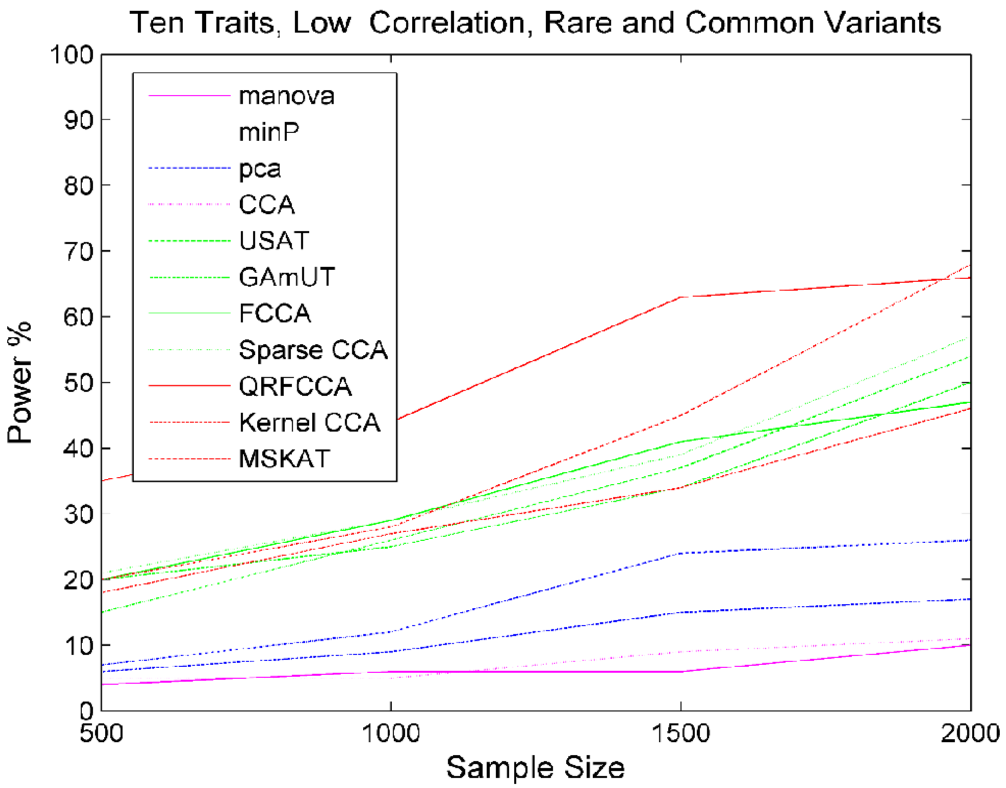


**Fig S23.** The power of curves as a function of sample sizes of 11 statistics for collectively testing the association of the gene including both rare and common variants with 10 traits with low correlations for the scenario 3 at the significance level $\alpha = 0.05$ .

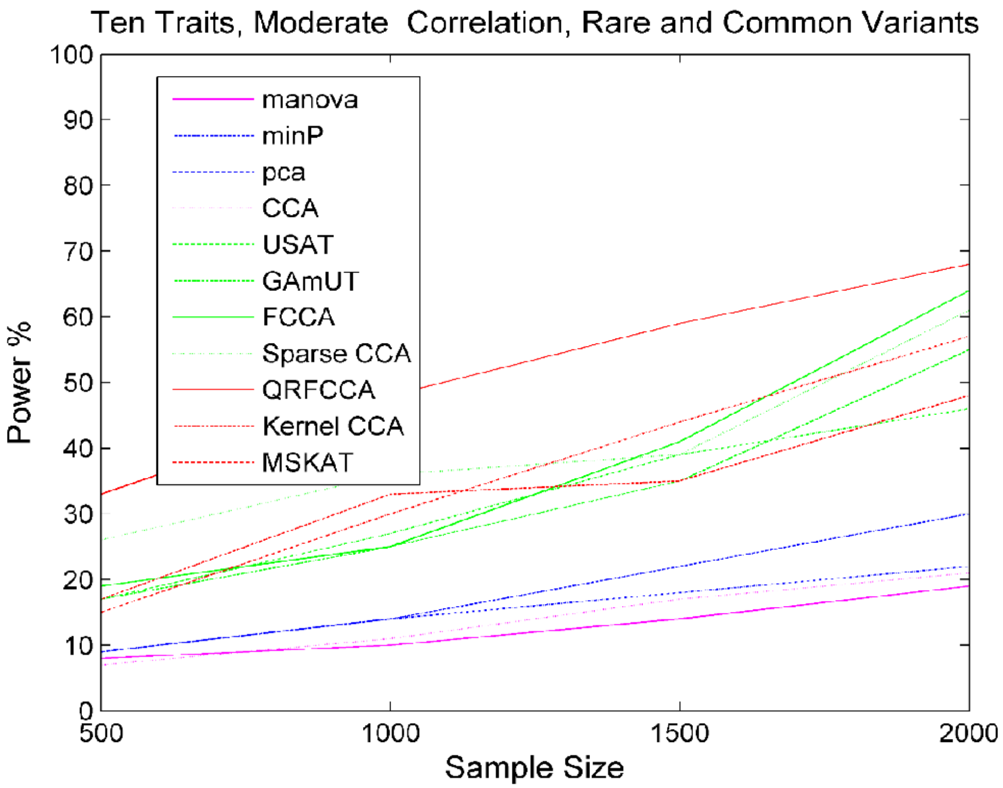


**Fig S24**. The power of curves as a function of sample sizes of 11 statistics for collectively testing the association of the gene including both rare and common variants with 10 traits with moderate correlations for the scenario 3 at the significance level $\alpha = 0.05$ .

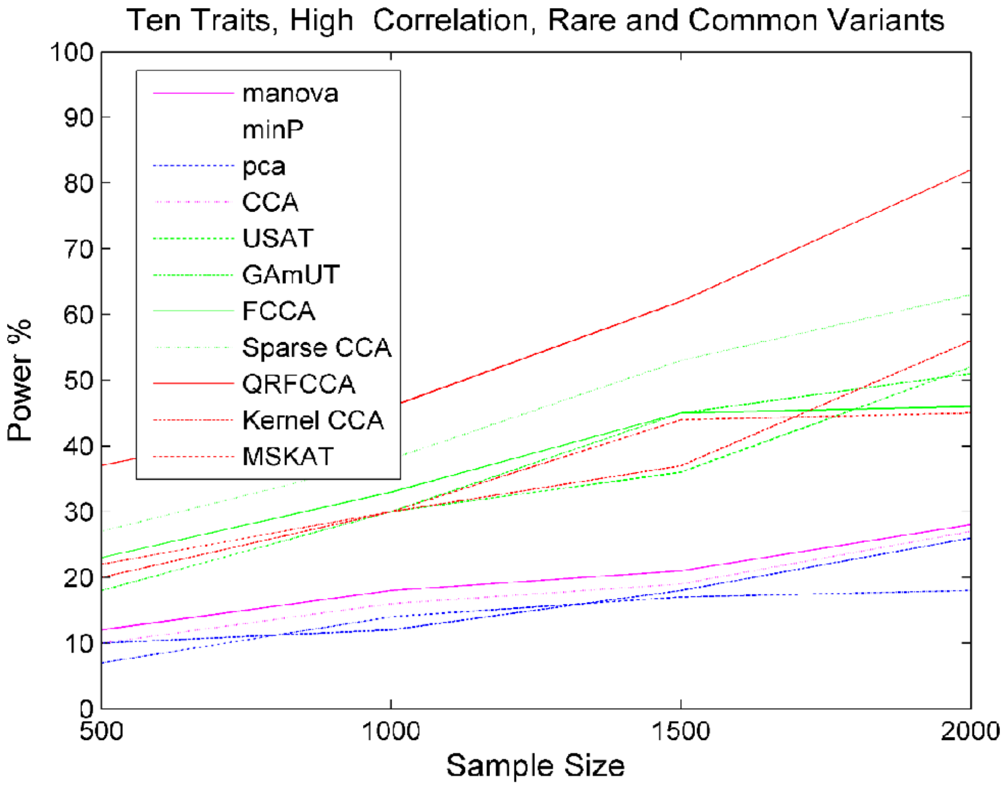


**Fig S25.** The power of curves as a function of sample sizes of 11 statistics for collectively testing the association of the gene including both rare and common variants with 10 traits with high correlations for the scenario 3 at the significance level $\alpha = 0.05$.

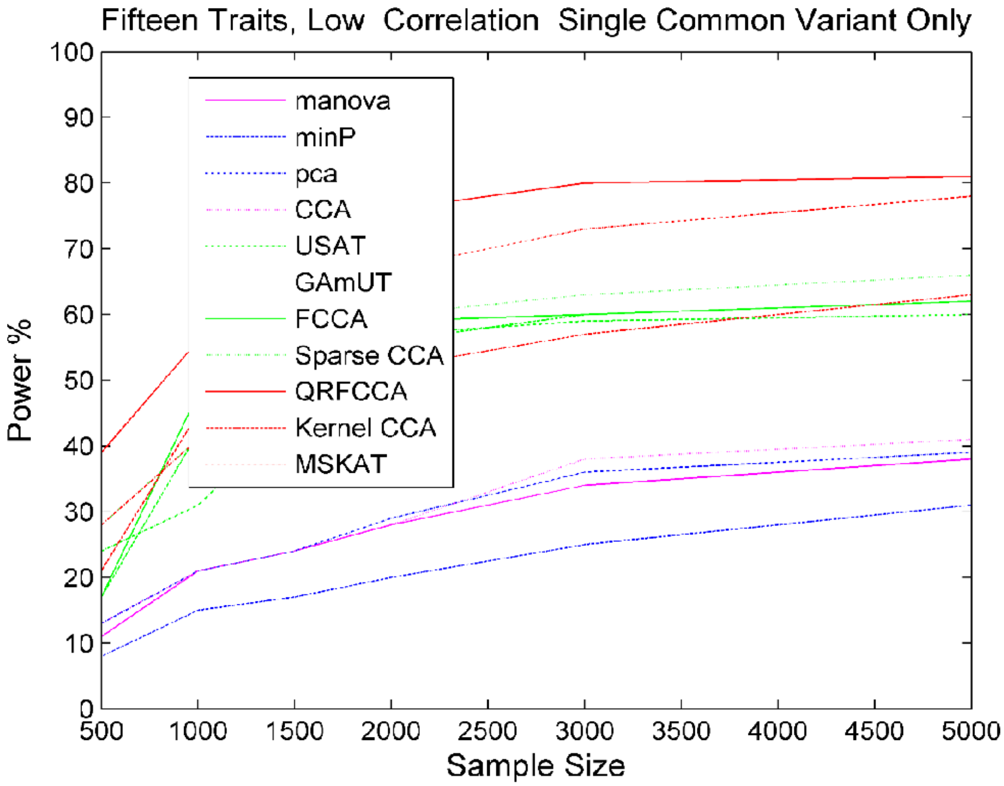


**Fig S26.** The power of curves as a function of sample sizes of 11 statistics for testing the power of a single common variant with 15 low correlated traits at the significance level $\alpha = 0.05$.

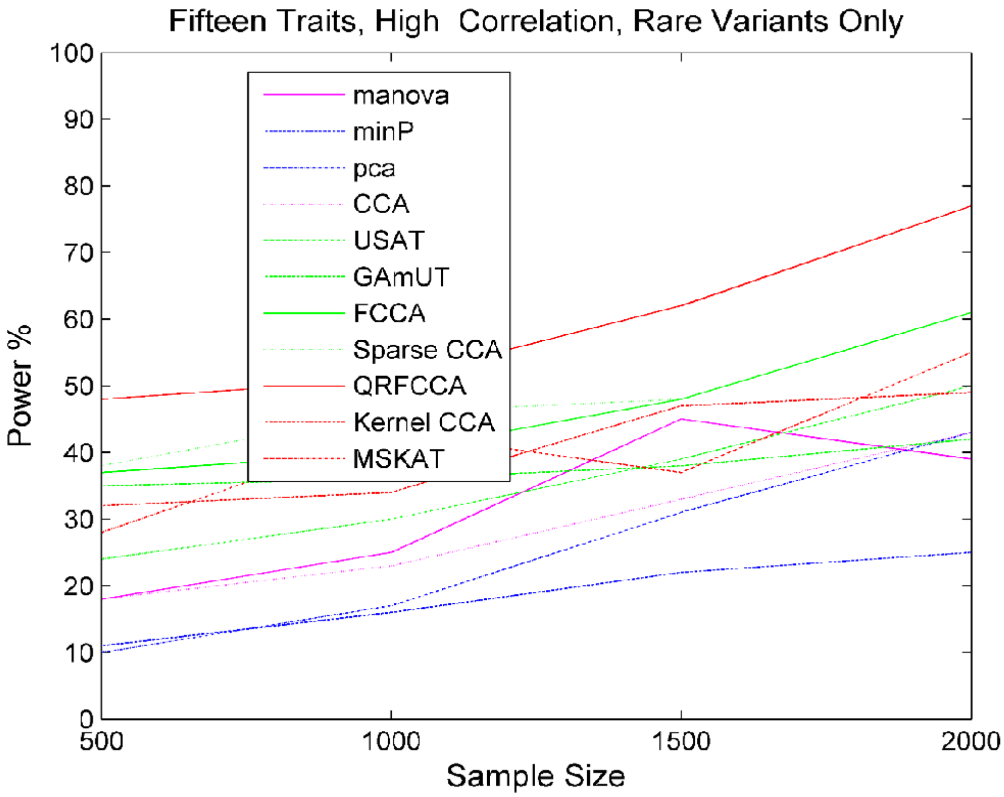


**Fig S27.** The power of curves as a function of sample sizes of 11 statistics for collectively testing the association of the gene including rare variants only with 15 highly correlated traits in scenario 1 at the significance level $\alpha = 0.00001$ .

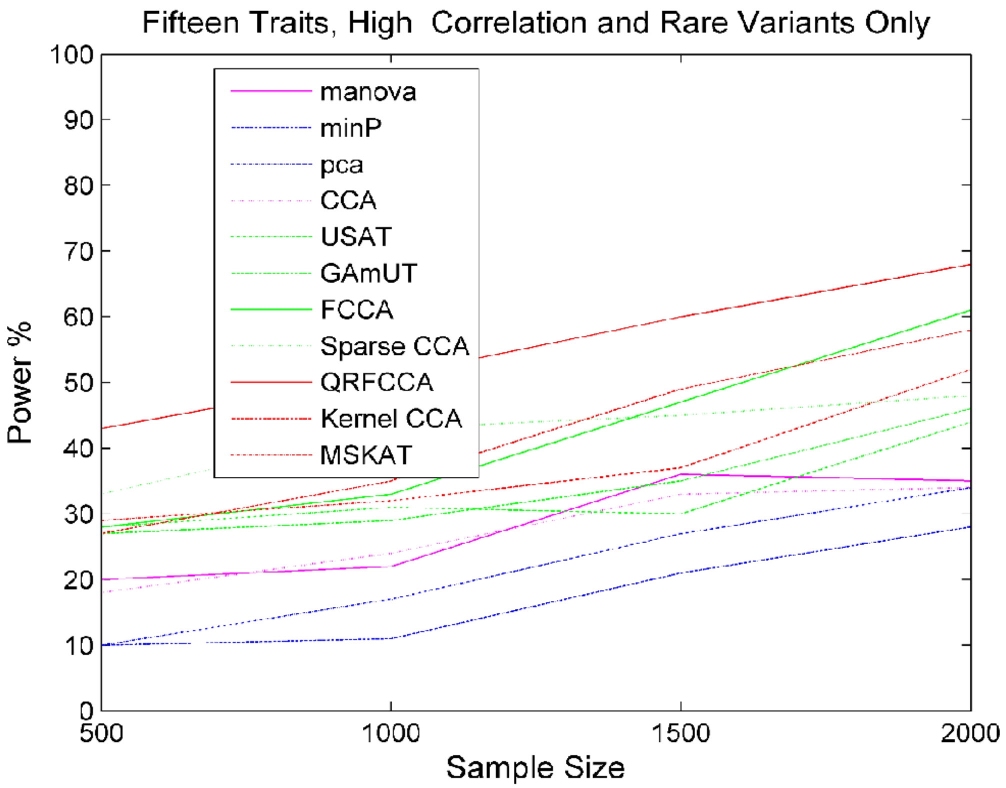


**Fig S28.** The power of curves as a function of sample sizes of 11 statistics for collectively testing the association of the gene including rare variants only with 15 highly correlated traits in scenario 2 at the significance level $\alpha = 0.00001$ .

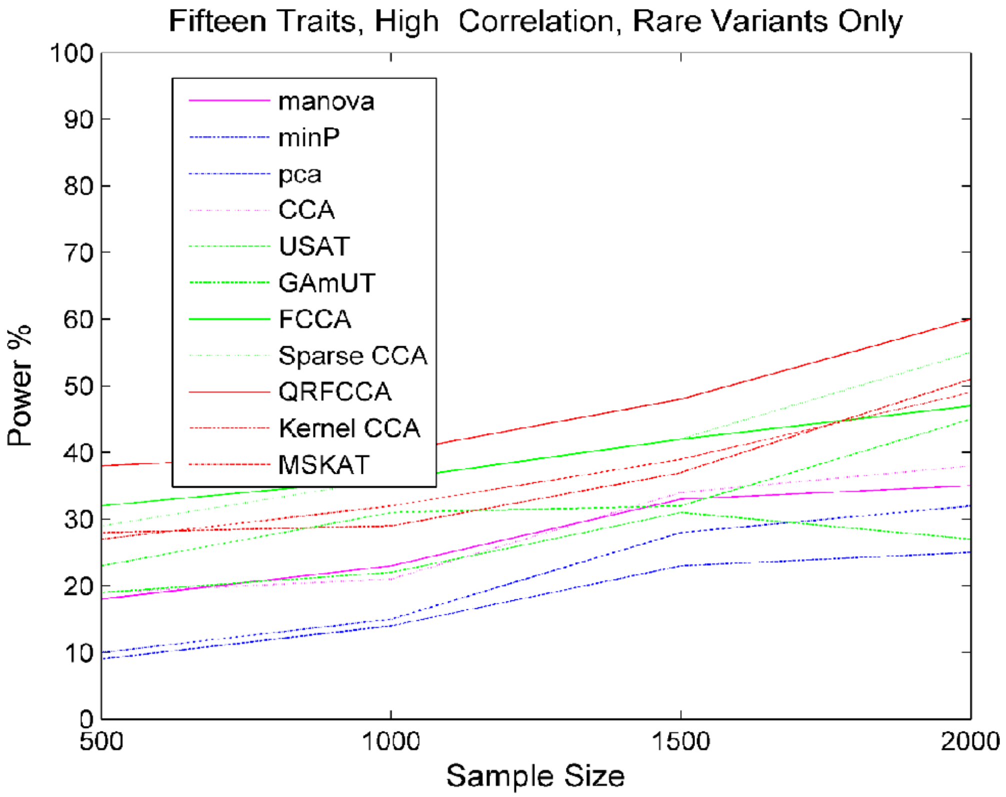


**Fig S29.** The power of curves as a function of sample sizes of 11 statistics for collectively testing the association of the gene including rare variants only with 15 highly correlated traits in scenario 3 at the significance level $\alpha = 0.00001$ .

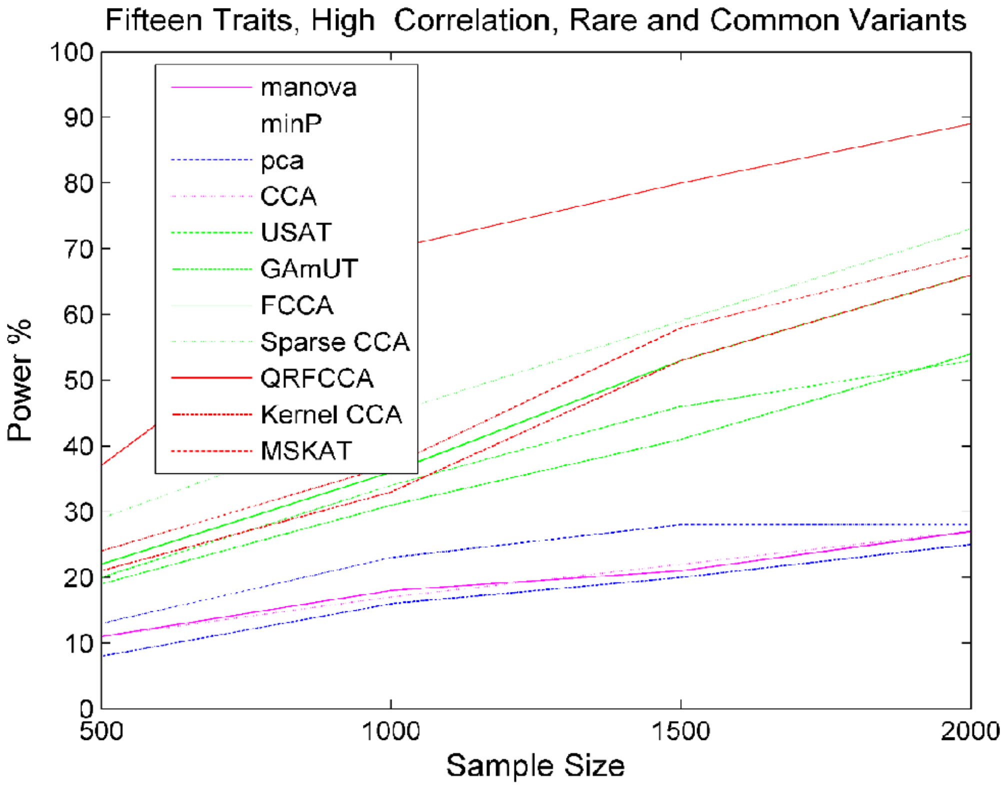


**Fig S30.** The power of curves as a function of sample sizes of 11 statistics for collectively testing the association of the gene including both rare and common variants with 15 highly correlated traits in scenario 1 at the significance level $\alpha = 0.00001$ .

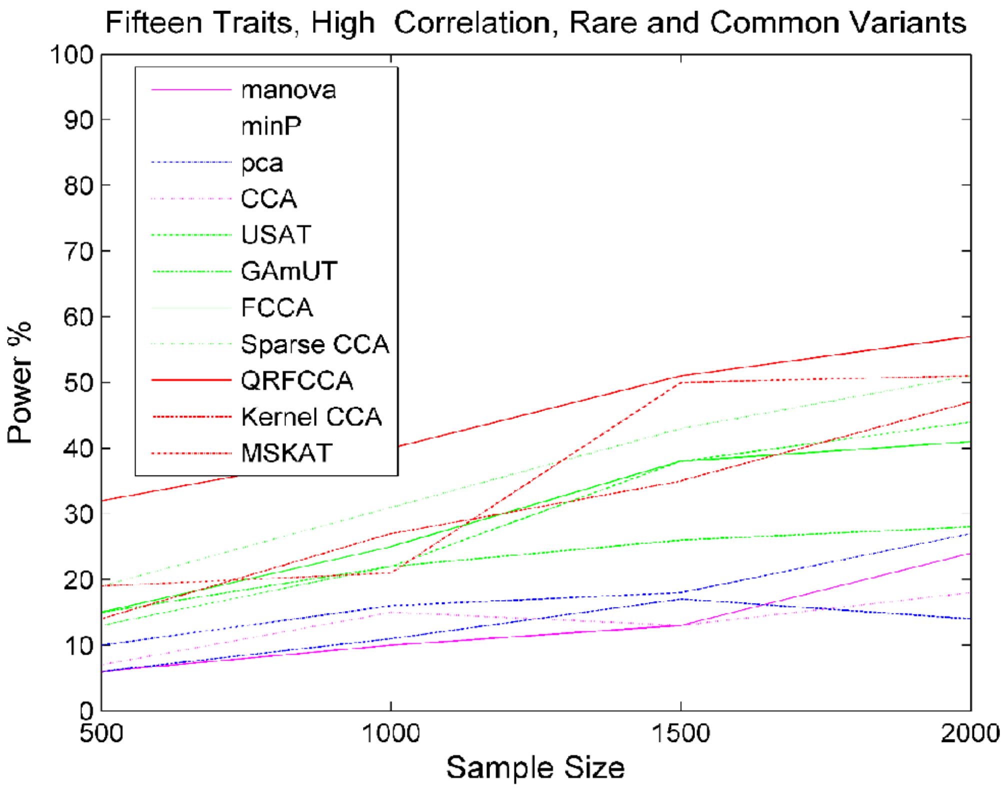


**Fig S31.** The power of curves as a function of sample sizes of 11 statistics for collectively testing the association of the gene including both rare and common variants with 15 highly correlated traits in scenario 2 at the significance level $\alpha = 0.00001$ .

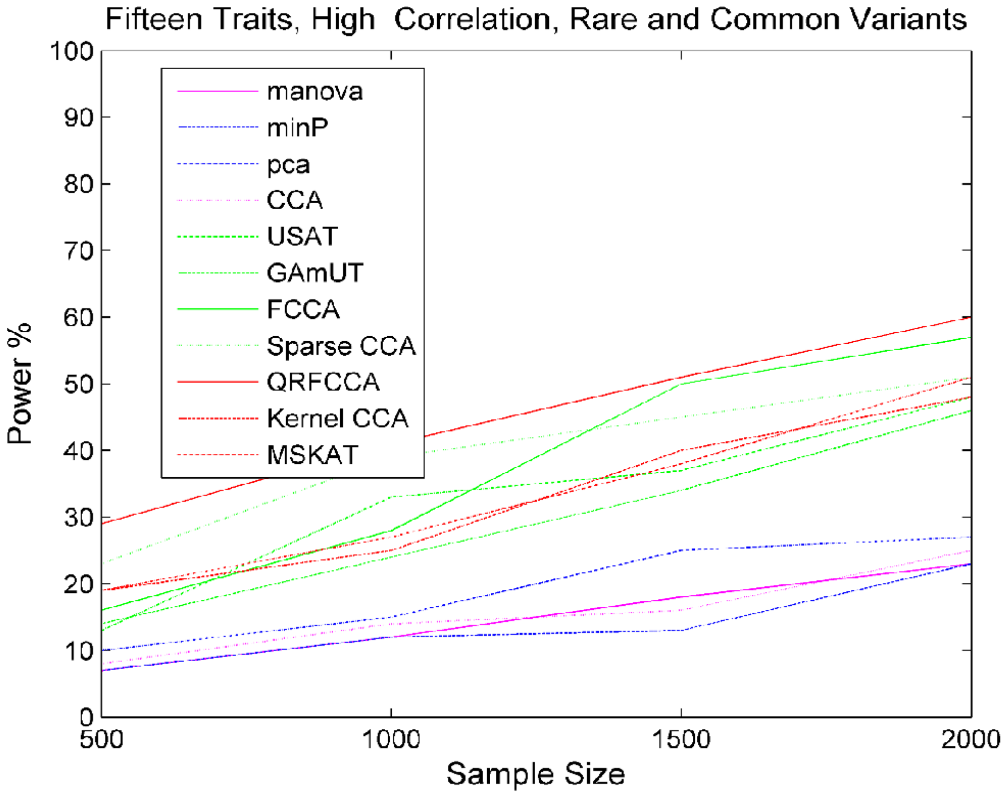


**Fig S32.** The power of curves as a function of sample sizes of 11 statistics for collectively testing the association of the gene including both rare and common variants with 15 highly correlated traits in scenario 3 at the significance level $\alpha = 0.00001$ .

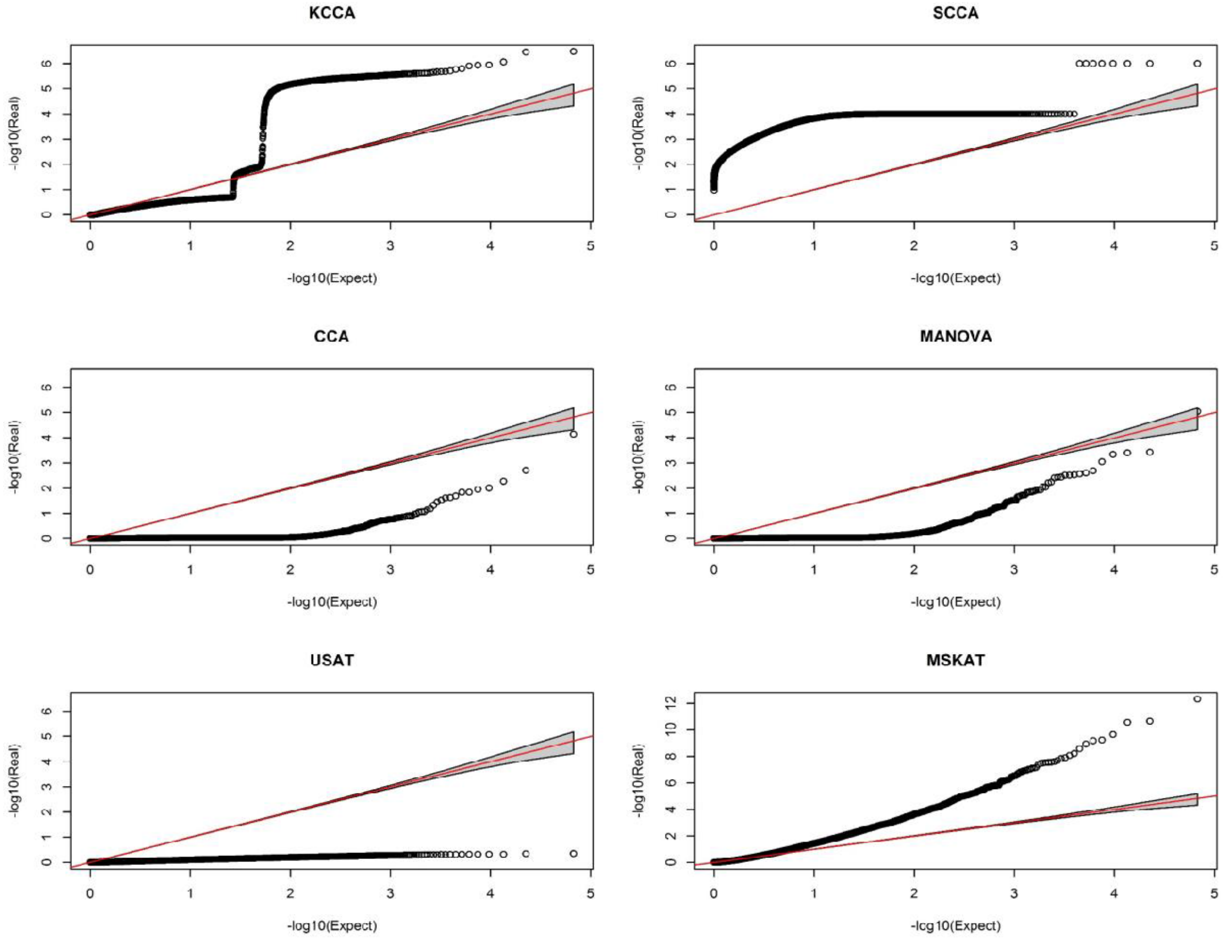


**Fig S33.** QQ plot of MSKAT, KCCA, CCA, USAT, SCCA, and MANOVA with 95% confidence interval for rare variants. The negative logarithm of the observed ( $y$ axis) and the expected ( $x$ axis) P value is plotted for each gene (dot), and the red line indicates the null hypothesis of no true association.

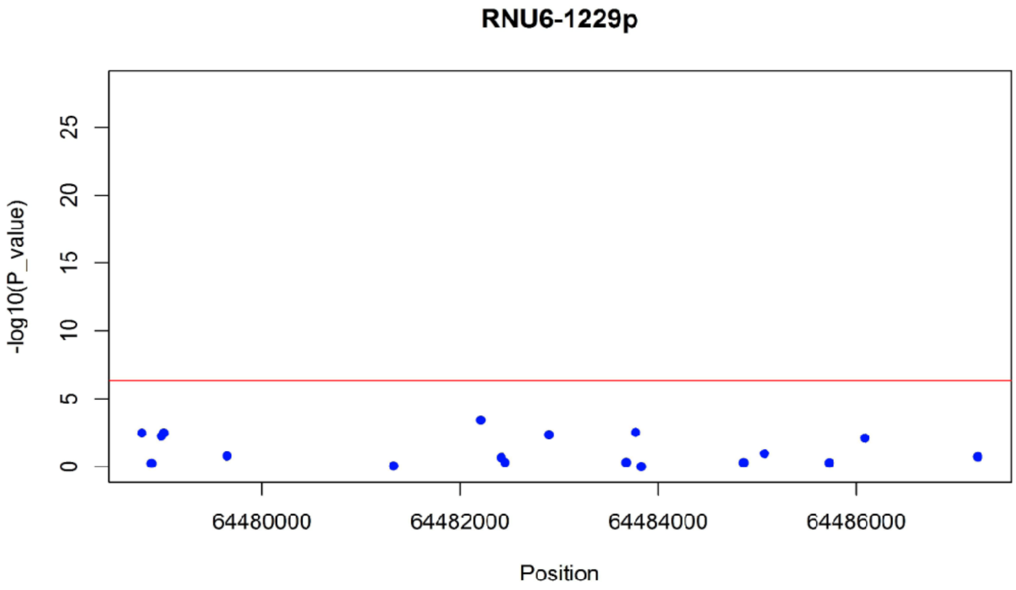


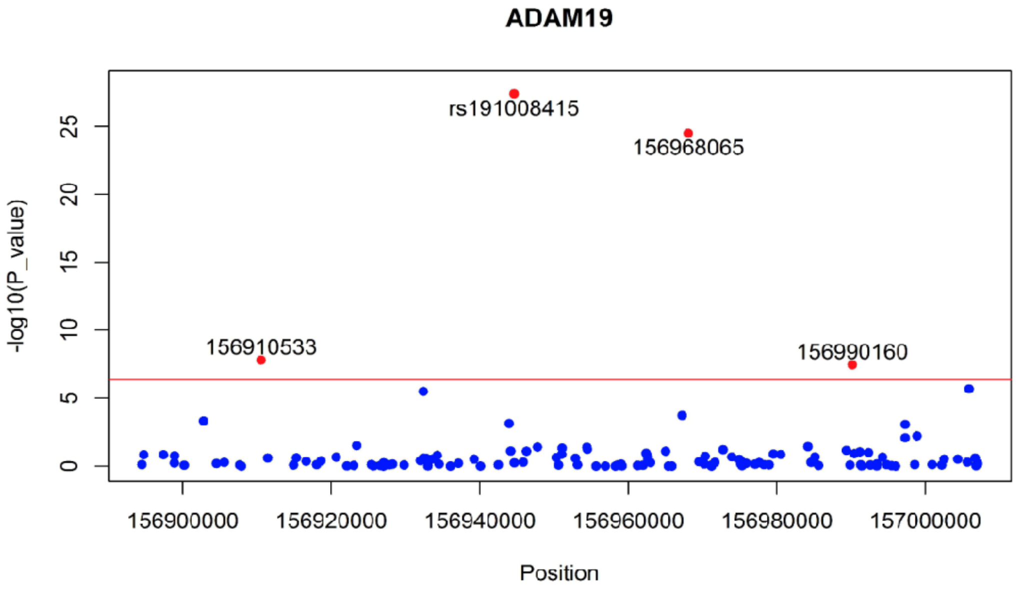


**Fig S34**. Association signals of SNPs within genes *RNU6-1229P* and *ADAM19*. In the top plot, we showed the P-valuesof the SNPs within *RNU6-1229P* associated with 46 traits. In the bottom plot, we showed the P-valuesof the SNPs within *ADAM19* associated with 46 traits where the numbers next to the red points were the RS number or genomic position of the SNPs.

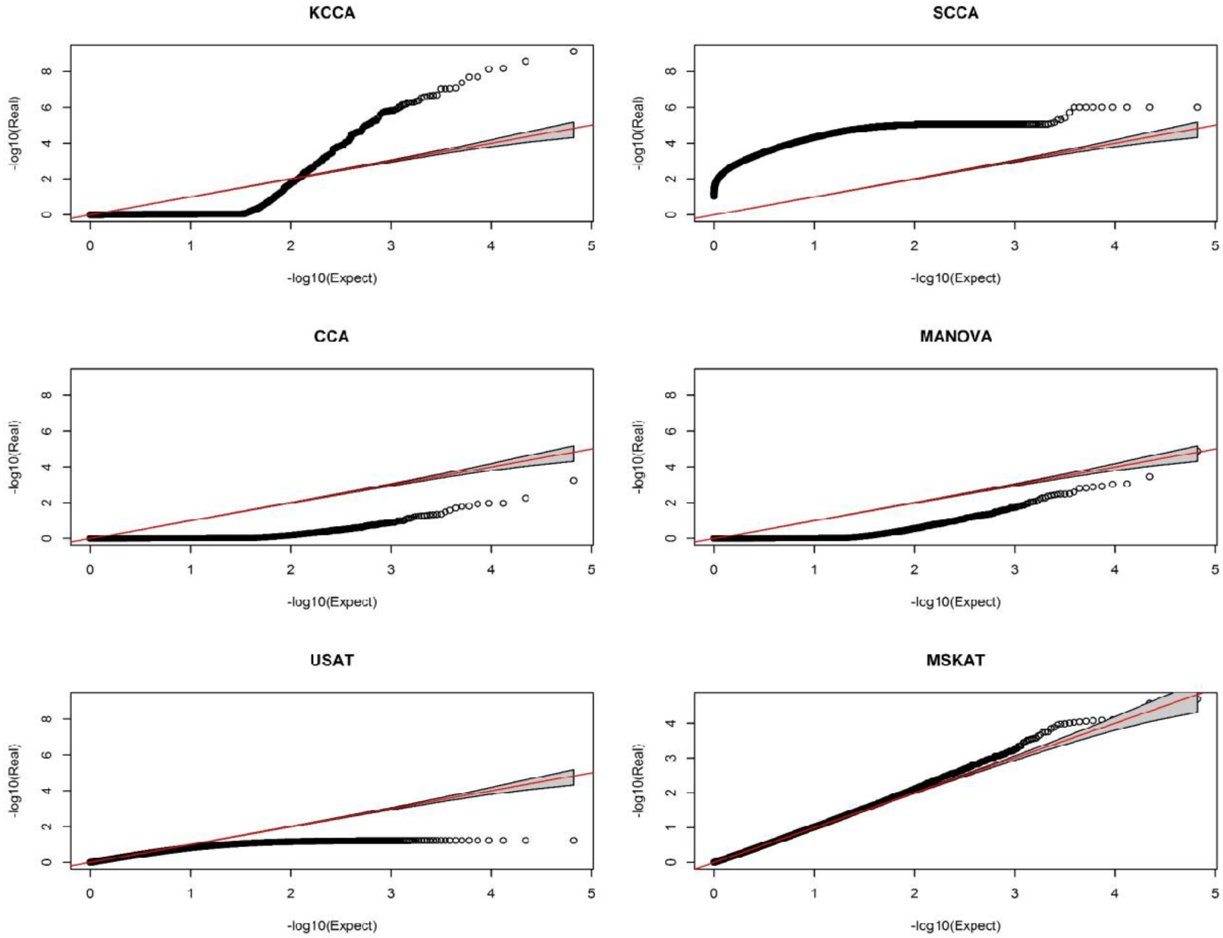


**Fig S35**. QQ plot of KCCA, CCA, USAT, SCCA, and MANOVA with 95% confidence interval for common variants. The negative logarithm of the observed ( $y$ axis) and the expected ( $x$ axis) P value is plotted for each gene (dot), and the red line indicates the null hypothesis of no true association.

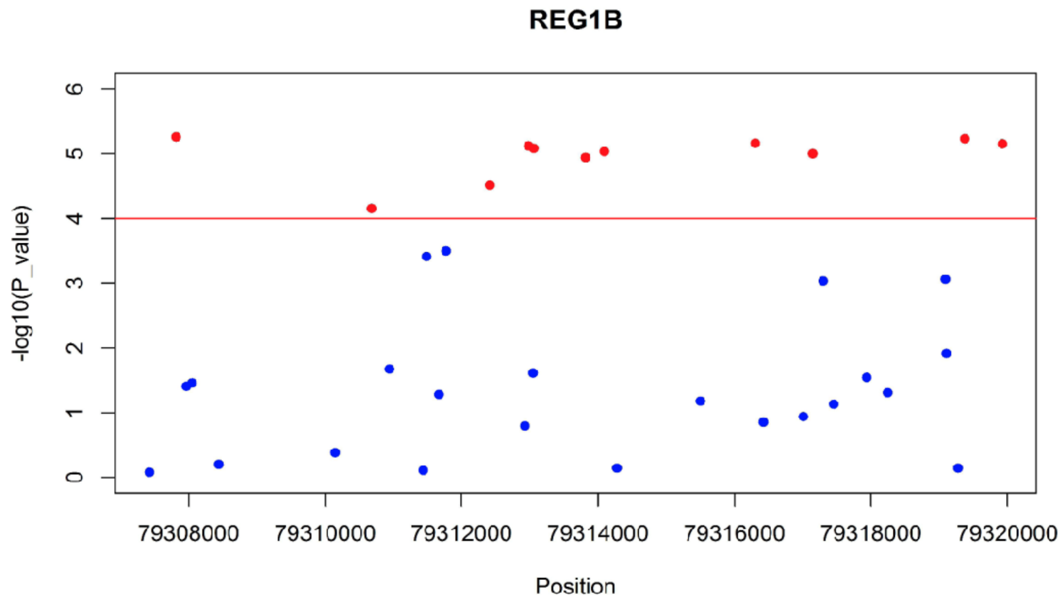


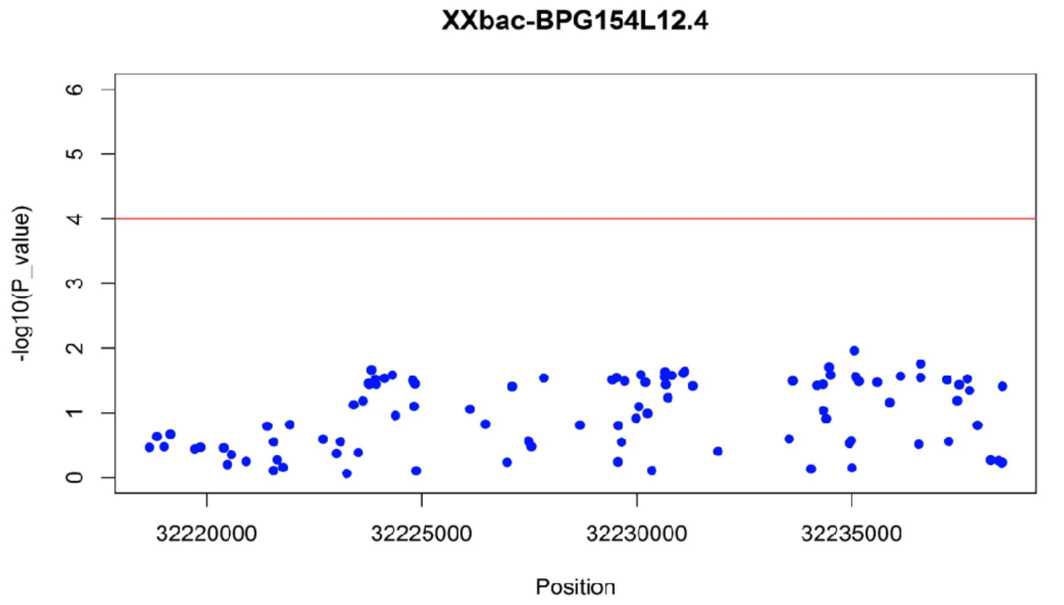


**Fig S36.** Association signals of SNPs within genes *REG1B* and *XXbac-BPG154L12.4*. In the top plot, we showed the P-values of the SNPs within *REG1B* associated with the 46 traits. In the bottom plot, we showed the P-values of the SNPs within *XXbac-BPG154L12.4* associated with the 46 traits where the numbers next to the red points were the RS number or genomic position of the SNPs.
