# Supplementary material for: A quadratically regularized functional canonical correlation analysis for identifying the global structure of pleiotropy with NGS data
Source: PLoS Comput Biol. 2017 Oct 17;13(10):e1005788. doi: 10.1371/journal.pcbi.1005788 (PMC5659802; doi:10.1371/journal.pcbi.1005788)
Supplement: S1 Table — Table S1–Table S25. (PDF) [file pcbi.1005788.s003.pdf]

Table S1. Type 1 error rates of 11 statistics for testing the association of rare variants in a gene with 5 low correlated traits.

Sample Size	Nominal Level	QRFCCA	FCCA	GAMuT	SCCA	USAT	MANOVA	CCA	PCA	KCCA	MSKAT	minP
500	0.05	0.0501	0.0508	0.0479	0.0498	0.0382	0.0498	0.0465	0.0527	0.0465	0.05	0.0297
	0.01	0.0097	0.0106	0.0099	0.0102	0.0082	0.0093	0.0097	0.0101	0.0095	0.0103	0.0069
	0.001	0.001	0.001	0.0009	0.001	0.0008	0.001	0.001	0.0011	0.001	0.0009	0.0007
	0.0001	0.0001	0.0001	0.00009	0.0001	0.0001	0.0001	0.00011	0.00011	0.0001	0.0001	0.00011
	0.00001	0.00001	0.00001	0.00001	0.00001	0.00001	0.00001	0.00001	0.00001	0.00001	0.00001	0.00001
1000	0.05	0.0548	0.0479	0.052	0.0509	0.0582	0.0498	0.0499	0.0464	0.045	0.0495	0.026
	0.01	0.0097	0.0097	0.0118	0.0097	0.011	0.0103	0.0106	0.0095	0.0087	0.0099	0.0053
	0.001	0.001	0.001	0.0011	0.001	0.0012	0.001	0.001	0.001	0.001	0.001	0.0005
	0.0001	0.00009	0.00009	0.0001	0.00009	0.0001	0.0001	0.0001	0.0001	0.0001	0.0001	0.0001
	0.00001	0.00001	0.00001	0.00001	0.00001	0.00001	0.00001	0.00001	0.00001	0.00001	0.00001	0.00001
2000	0.05	0.0522	0.0522	0.0568	0.0507	0.063	0.0472	0.0488	0.0506	0.0483	0.0482	0.0256
	0.01	0.0101	0.0105	0.0108	0.0096	0.0135	0.0099	0.0107	0.0096	0.0096	0.0098	0.0056
	0.001	0.001	0.0009	0.0011	0.001	0.0013	0.0011	0.001	0.001	0.0008	0.001	0.0005
	0.001	0.00104	0.00095	0.00105	0.00102	0.00112	0.00101	0.00108	0.00101	0.001	0.00099	0.00107
	0.00001	0.00001	0.00001	0.00001	0.00001	0.00001	0.00001	0.00001	0.00001	0.00001	0.00001	0.00001

Table S2. Type 1 error rates of 11 statistics for testing the association of rare variants in a gene with 5 moderately correlated traits.

Sample Size	Nominal Level	QRFCCA	FCCA	GAMuT	SCCA	USAT	MANOVA	CCA	PCA	KCCA	MSKAT	minP
500	0.05	0.0531	0.0532	0.0503	0.0534	0.0373	0.0457	0.0484	0.0512	0.0514	0.0519	0.0323
	0.01	0.0101	0.0096	0.0097	0.0107	0.0074	0.0105	0.0092	0.0101	0.0093	0.0101	0.006
	0.001	0.001	0.001	0.001	0.001	0.0008	0.0009	0.0009	0.001	0.0009	0.0011	0.0006
	0.0001	0.00011	0.00012	0.00011	0.00011	0.00012	0.0001	0.00011	0.00011	0.00011	0.0001	0.0001
	0.00001	0.00001	0.00001	0.00001	0.00001	0.00001	0.00001	0.00001	0.00001	0.00001	0.00001	0.00001
1000	0.05	0.0487	0.0497	0.0551	0.0534	0.0578	0.0508	0.0514	0.0507	0.0448	0.0491	0.0256
	0.01	0.01	0.0096	0.0108	0.0101	0.0123	0.0102	0.0101	0.0097	0.0091	0.0102	0.0049
	0.001	0.0011	0.001	0.0012	0.001	0.0012	0.001	0.001	0.001	0.0009	0.0009	0.0005
	0.0001	0.00009	0.00011	0.00012	0.0001	0.00011	0.00011	0.0001	0.00011	0.00009	0.0001	0.00011
	0.00001	0.00001	0.00001	0.00001	0.00001	0.00001	0.00001	0.00001	0.00001	0.00001	0.00001	0.00001
2000	0.05	0.048	0.0475	0.0513	0.0528	0.0637	0.0483	0.0505	0.0511	0.0473	0.0476	0.0252
	0.01	0.0105	0.0107	0.0112	0.0106	0.0125	0.0093	0.0099	0.0098	0.0104	0.0098	0.0048
	0.001	0.0009	0.0009	0.0011	0.001	0.0013	0.001	0.001	0.001	0.0008	0.001	0.0005
	0.001	0.00008	0.00009	0.00009	0.00008	0.00009	0.0001	0.0001	0.0001	0.00008	0.0001	0.00009
	0.00001	0.00001	0.00001	0.00001	0.00001	0.00001	0.00001	0.00001	0.00001	0.00001	0.00001	0.00001

Table S3. Type 1 error rates of 11 statistics for testing the association of rare variants in a gene with 5 highly correlated traits.

Sample Size	Nominal Level	QRFCCA	FCCA	GAMuT	SCCA	USAT	MANOVA	CCA	PCA	KCCA	MSKAT	minP
500	0.05	0.0489	0.0524	0.0495	0.0509	0.0419	0.0485	0.0527	0.049	0.0522	0.0503	0.0297
	0.01	0.0103	0.0097	0.0108	0.0096	0.0077	0.01	0.0102	0.0102	0.0091	0.0105	0.0055
	0.001	0.001	0.001	0.0011	0.001	0.0008	0.0011	0.001	0.001	0.0009	0.0011	0.0006
	0.0001	0.00009	0.00009	0.00009	0.00009	0.00009	0.0001	0.0001	0.0001	0.00009	0.0001	0.0001
	0.00001	0.00001	0.00001	0.00001	0.00001	0.00001	0.00001	0.00001	0.00001	0.00001	0.00001	0.00001
1000	0.05	0.0495	0.0498	0.0531	0.0513	0.0563	0.0504	0.049	0.0478	0.0485	0.0486	0.0272
	0.01	0.0099	0.0097	0.0117	0.0098	0.0113	0.0098	0.0101	0.0101	0.0089	0.0097	0.0048
	0.001	0.001	0.001	0.0012	0.001	0.0011	0.001	0.001	0.0009	0.0009	0.0009	0.0006
	0.0001	0.00008	0.00009	0.00009	0.00008	0.0001	0.0001	0.0001	0.0001	0.00008	0.0001	0.0001
	0.00001	0.00001	0.00001	0.00001	0.00001	0.00001	0.00001	0.00001	0.00001	0.00001	0.00001	0.00001
2000	0.05	0.0533	0.0502	0.0593	0.0525	0.0638	0.0482	0.0491	0.0505	0.0475	0.054	0.0254
	0.01	0.0098	0.0096	0.0106	0.0098	0.0131	0.0099	0.01	0.0103	0.0092	0.0101	0.0052
	0.001	0.0011	0.0011	0.001	0.001	0.0013	0.001	0.001	0.001	0.001	0.0009	0.0006
	0.001	0.00011	0.00011	0.0001	0.00011	0.0001	0.0001	0.0001	0.00011	0.0001	0.00011	0.0001
	0.00001	0.00001	0.00001	0.00001	0.00001	0.00001	0.00001	0.00001	0.00001	0.00001	0.00001	0.00001

Table S4. Type 1 error rates of 11 statistics for testing the association of rare variants in a gene with 10 low correlated traits.

Sample Size	Nominal Level	QRFCCA	FCCA	GAMuT	SCCA	USAT	MANOVA	CCA	PCA	KCCA	MSKAT	minP
500	0.05	0.0478	0.0508	0.0472	0.0493	0.0393	0.0494	0.0521	0.0487	0.0456	0.0503	0.0271
	0.01	0.0104	0.0098	0.01	0.0102	0.0076	0.0105	0.0105	0.0098	0.0095	0.0103	0.0064
	0.001	0.001	0.001	0.001	0.001	0.0008	0.001	0.001	0.0011	0.001	0.001	0.0006
	0.0001	0.00008	0.00008	0.00008	0.00008	0.00009	0.0001	0.00009	0.00009	0.00008	0.00011	0.0001
	0.00001	0.00001	0.00001	0.00001	0.00001	0.00001	0.00001	0.00001	0.00001	0.00001	0.00001	0.00001
1000	0.05	0.0504	0.0474	0.0523	0.0495	0.0579	0.0475	0.0499	0.0513	0.0456	0.0484	0.0242
	0.01	0.0102	0.0106	0.0112	0.0096	0.0111	0.0099	0.01	0.0103	0.0103	0.0094	0.0055
	0.001	0.001	0.001	0.0011	0.001	0.0011	0.001	0.001	0.0011	0.001	0.001	0.0006
	0.0001	0.00011	0.00011	0.00012	0.00011	0.00011	0.0001	0.00011	0.00011	0.00012	0.0001	0.0001
	0.00001	0.00001	0.00001	0.00001	0.00001	0.00001	0.00001	0.00001	0.00001	0.00001	0.00001	0.00001
2000	0.05	0.0501	0.0541	0.0553	0.0503	0.0677	0.0494	0.049	0.0516	0.0522	0.05	0.0247
	0.01	0.0109	0.0102	0.0102	0.0098	0.0131	0.0104	0.0102	0.0104	0.0093	0.01	0.0055
	0.001	0.001	0.001	0.0011	0.001	0.0012	0.001	0.001	0.001	0.001	0.0011	0.0005
	0.001	0.0001	0.0001	0.0001	0.0001	0.0001	0.0001	0.00011	0.00011	0.0001	0.0001	0.00011
	0.00001	0.00001	0.00001	0.00001	0.00001	0.00001	0.00001	0.00001	0.00001	0.00001	0.00001	0.00001

Table S5. Type 1 error rates of 11 statistics for testing the association of rare variants in a gene with 10 moderately correlated traits.

Sample Size	Nominal Level	QRFCCA	FCCA	GAMuT	SCCA	USAT	MANOVA	CCA	PCA	KCCA	MSKAT	minP
	0.05	0.0509	0.049	0.0469	0.0515	0.042	0.0497	0.0461	0.0516	0.0475	0.0498	0.0303
	0.01	0.0104	0.0099	0.01	0.0105	0.0072	0.0099	0.01	0.0097	0.0091	0.0097	0.0061
500	0.001	0.001	0.001	0.001	0.001	0.0007	0.001	0.001	0.001	0.001	0.0009	0.0006
	0.0001	0.0001	0.0001	0.0001	0.0001	0.0001	0.0001	0.0001	0.0001	0.00009	0.0001	0.00011
	0.00001	0.00001	0.00001	0.00001	0.00001	0.00001	0.00001	0.00001	0.00001	0.00001	0.00001	0.00001
	0.05	0.053	0.0517	0.0549	0.0506	0.0592	0.0467	0.0512	0.0487	0.0502	0.0511	0.0287
	0.01	0.0104	0.0106	0.0109	0.0095	0.0114	0.01	0.0102	0.0103	0.0106	0.01	0.0047
1000	0.001	0.0009	0.001	0.0011	0.001	0.0012	0.001	0.001	0.001	0.0009	0.001	0.0005
	0.0001	0.0001	0.00012	0.00011	0.00011	0.00011	0.0001	0.00011	0.0001	0.0001	0.00011	0.0001
	0.00001	0.00001	0.00001	0.00001	0.00001	0.00001	0.00001	0.00001	0.00001	0.00001	0.00001	0.00001
	0.05	0.0485	0.0533	0.0568	0.0499	0.0635	0.0498	0.0519	0.0533	0.0483	0.0487	0.0284
	0.01	0.0091	0.0095	0.011	0.0101	0.0128	0.0103	0.0099	0.0098	0.0087	0.01	0.0057
2000	0.001	0.001	0.001	0.0011	0.0011	0.0013	0.0011	0.001	0.001	0.001	0.001	0.0006
	0.001	0.00009	0.00009	0.00009	0.00009	0.0001	0.0001	0.0001	0.0001	0.00009	0.0001	0.0001
	0.00001	0.00001	0.00001	0.00001	0.00001	0.00001	0.00001	0.00001	0.00001	0.00001	0.00001	0.00001

Table S6. Type 1 error rates of 11 statistics for testing the association of rare variants in a gene with 10 highly correlated traits

Sample Size	Nominal Level	QRFCCA	FCCA	GAMuT	SCCA	USAT	MANOVA	CCA	PCA	KCCA	MSKAT	minP
	0.05	0.0495	0.0515	0.0516	0.046	0.0408	0.0536	0.0476	0.0544	0.0482	0.0487	0.0293
	0.01	0.0094	0.01	0.0101	0.0096	0.0083	0.0091	0.0093	0.0101	0.0099	0.0102	0.0061
500	0.001	0.001	0.001	0.001	0.001	0.0008	0.001	0.001	0.001	0.0009	0.001	0.0006
	0.0001	0.00009	0.00009	0.00009	0.00009	0.00009	0.0001	0.0001	0.00009	0.00009	0.0001	0.0001
	0.00001	0.00001	0.00001	0.00001	0.00001	0.00001	0.00001	0.00001	0.00001	0.00001	0.00001	0.00001
	0.05	0.0507	0.0512	0.0549	0.0462	0.0547	0.0521	0.052	0.049	0.0481	0.0464	0.0248
	0.01	0.0103	0.01	0.0113	0.0108	0.0108	0.0106	0.01	0.0097	0.0093	0.0104	0.0049
1000	0.001	0.001	0.001	0.0011	0.001	0.0011	0.001	0.001	0.0011	0.001	0.0009	0.0006
	0.0001	0.00009	0.00009	0.00009	0.00009	0.00009	0.0001	0.00008	0.00009	0.00009	0.0001	0.00009
	0.00001	0.00001	0.00001	0.00001	0.00001	0.00001	0.00001	0.00001	0.00001	0.00001	0.00001	0.00001
	0.05	0.0517	0.049	0.0557	0.0503	0.0667	0.0472	0.0474	0.0465	0.0476	0.0468	0.0288
	0.01	0.0102	0.0097	0.0106	0.0104	0.0131	0.0103	0.0097	0.0103	0.009	0.0102	0.0058
2000	0.001	0.001	0.001	0.001	0.001	0.0013	0.001	0.001	0.001	0.0009	0.001	0.0005
	0.001	0.00008	0.00009	0.00008	0.00008	0.00009	0.00009	0.00009	0.00009	0.00008	0.0001	0.00009
	0.00001	0.00001	0.00001	0.00001	0.00001	0.00001	0.00001	0.00001	0.00001	0.00001	0.00001	0.00001

Table S7. Type 1 error rates of 11 statistics for testing the association of rare variants in a gene with 15 low correlated traits.

Sample Size	Nominal Level	QRFCCA	FCCA	GAMuT	SCCA	USAT	MANOVA	CCA	PCA	KCCA	MSKAT	minP
500	0.05	0.0477	0.0475	0.0535	0.0523	0.0419	0.0524	0.0468	0.0496	0.0439	0.052	0.0321
	0.01	0.0098	0.01	0.0091	0.0096	0.0075	0.0097	0.0106	0.0108	0.0097	0.0104	0.0061
	0.001	0.001	0.0011	0.0009	0.001	0.0008	0.0011	0.0009	0.001	0.001	0.001	0.0006
	0.0001	0.00013	0.00012	0.00012	0.00012	0.00011	0.00011	0.00011	0.00011	0.00012	0.0001	0.0001
	0.00001	0.00001	0.00001	0.00001	0.00001	0.00001	0.00001	0.00001	0.00001	0.00001	0.00001	0.00001
1000	0.05	0.0479	0.0501	0.0569	0.0538	0.0578	0.048	0.0481	0.0508	0.0461	0.0506	0.0258
	0.01	0.0093	0.0095	0.0106	0.0099	0.0114	0.0103	0.0094	0.0094	0.009	0.0099	0.0049
	0.001	0.0011	0.001	0.001	0.0011	0.0012	0.001	0.001	0.001	0.001	0.001	0.0005
	0.0001	0.00012	0.00011	0.00012	0.00012	0.00011	0.00011	0.00011	0.00011	0.00013	0.0001	0.00011
	0.00001	0.00001	0.00001	0.00001	0.00001	0.00001	0.00001	0.00001	0.00001	0.00001	0.00001	0.00001
2000	0.05	0.051	0.0479	0.0522	0.0488	0.0621	0.0505	0.0502	0.0531	0.0442	0.0487	0.0249
	0.01	0.0098	0.0099	0.0111	0.0097	0.0126	0.0096	0.0105	0.0097	0.0097	0.0098	0.005
	0.001	0.001	0.001	0.001	0.0009	0.0013	0.001	0.0009	0.001	0.001	0.001	0.0005
	0.001	0.0001	0.00009	0.00009	0.00009	0.00009	0.00011	0.0001	0.0001	0.00009	0.00009	0.0001
	0.00001	0.00001	0.00001	0.00001	0.00001	0.00001	0.00001	0.00001	0.00001	0.00001	0.00001	0.00001

Table S8. Type 1 error rates of 11 statistics for testing the association of rare variants in a gene with 15 moderately correlated traits.

Sample Size	Nominal Level	QRFCCA	FCCA	GAMuT	SCCA	USAT	MANOVA	CCA	PCA	KCCA	MSKAT	minP
	0.05	0.0493	0.0519	0.0502	0.0523	0.0417	0.0512	0.0526	0.048	0.0507	0.0523	0.0343
	0.01	0.0099	0.0107	0.0104	0.0102	0.0082	0.0101	0.0108	0.0104	0.0101	0.0097	0.0057
500	0.001	0.0011	0.0011	0.001	0.001	0.0008	0.001	0.001	0.001	0.0011	0.001	0.0006
	0.0001	0.00011	0.00012	0.00011	0.00012	0.00011	0.0001	0.0001	0.00011	0.0001	0.00011	0.0001
	0.00001	0.00001	0.00001	0.00001	0.00001	0.00001	0.00001	0.00001	0.00001	0.00001	0.00001	0.00001
	0.05	0.0487	0.0481	0.0543	0.0485	0.0535	0.0491	0.0509	0.0499	0.0478	0.0529	0.0271
	0.01	0.01	0.0107	0.0108	0.0102	0.0121	0.0108	0.01	0.0107	0.0106	0.0099	0.0049
1000	0.001	0.001	0.0009	0.0011	0.001	0.0011	0.001	0.001	0.001	0.0008	0.001	0.0005
	0.0001	0.00011	0.00011	0.00011	0.00011	0.00011	0.0001	0.00011	0.00011	0.00012	0.0001	0.0001
	0.00001	0.00001	0.00001	0.00001	0.00001	0.00001	0.00001	0.00001	0.00001	0.00001	0.00001	0.00001
	0.05	0.0518	0.0514	0.0549	0.0501	0.0628	0.0499	0.0489	0.0471	0.0508	0.0483	0.0284
	0.01	0.0101	0.0101	0.0107	0.0105	0.0133	0.0094	0.0102	0.0097	0.0093	0.0095	0.0053
2000	0.001	0.001	0.001	0.0011	0.001	0.0013	0.0011	0.0009	0.0009	0.0009	0.0009	0.0006
	0.001	0.00011	0.0001	0.0001	0.00011	0.00009	0.0001	0.0001	0.0001	0.0001	0.00011	0.00011
	0.00001	0.00001	0.00001	0.00001	0.00001	0.00001	0.00001	0.00001	0.00001	0.00001	0.00001	0.00001

Table S9. Type 1 error rates of 11 statistics for testing the association of common and rare variants in a gene with 5 low correlated traits.

Sample Size	Nominal Level	QRFCCA	FCCA	GAMuT	SCCA	USAT	MANOVA	CCA	PCA	KCCA	MSKAT	minP
500	0.05	0.0524	0.0518	0.0537	0.0518	0.0402	0.049	0.0467	0.0529	0.0517	0.0493	0.0305
	0.01	0.0105	0.0105	0.0105	0.0097	0.0083	0.0107	0.0097	0.01	0.0104	0.0101	0.0065
	0.001	0.0009	0.001	0.001	0.001	0.0008	0.0009	0.001	0.0011	0.0009	0.0009	0.0006
	0.0001	0.00009	0.00009	0.00009	0.00009	0.00008	0.00009	0.00009	0.00009	0.00009	0.00011	0.00009
	0.00001	0.00001	0.00001	0.00001	0.00001	0.00001	0.00001	0.00001	0.00001	0.00001	0.00001	0.00001
1000	0.05	0.0472	0.0481	0.0525	0.0499	0.0577	0.0485	0.054	0.0482	0.0437	0.0524	0.0272
	0.01	0.0098	0.0096	0.0107	0.0104	0.0114	0.0096	0.01	0.0097	0.0093	0.0105	0.0054
	0.001	0.001	0.001	0.0011	0.001	0.0012	0.001	0.001	0.001	0.0009	0.001	0.0005
	0.0001	0.00008	0.00009	0.00009	0.00009	0.00009	0.0001	0.00009	0.00009	0.00008	0.0001	0.0001
	0.00001	0.00001	0.00001	0.00001	0.00001	0.00001	0.00001	0.00001	0.00001	0.00001	0.00001	0.00001
2000	0.05	0.0486	0.0508	0.0545	0.0534	0.0684	0.0484	0.0466	0.0508	0.0504	0.0513	0.026
	0.01	0.0103	0.0097	0.011	0.0106	0.0131	0.0099	0.0101	0.0095	0.0093	0.0104	0.0056
	0.001	0.001	0.001	0.0011	0.0009	0.0013	0.001	0.001	0.001	0.001	0.001	0.0006
	0.001	0.0001	0.0001	0.0001	0.00011	0.0001	0.0001	0.0001	0.0001	0.0001	0.0001	0.0001
	0.00001	0.00001	0.00001	0.00001	0.00001	0.00001	0.00001	0.00001	0.00001	0.00001	0.00001	0.00001

Table S10. Type 1 error rates of 11 statistics for testing the association of common and rare variants in a gene with 5 moderately correlated traits.

Sample Size	Nominal Level	QRFCCA	FCCA	GAMuT	SCCA	USAT	MANOVA	CCA	PCA	KCCA	MSKAT	minP
500	0.05	0.0478	0.0521	0.0508	0.0479	0.0375	0.0501	0.0518	0.0486	0.0469	0.0504	0.0309
	0.01	0.0104	0.0104	0.0103	0.0102	0.0073	0.0094	0.0107	0.0097	0.01	0.0093	0.0067
	0.001	0.001	0.001	0.001	0.001	0.0008	0.001	0.0011	0.001	0.001	0.001	0.0005
	0.0001	0.00008	0.00009	0.0001	0.00008	0.00009	0.0001	0.0001	0.00009	0.00008	0.0001	0.0001
	0.00001	0.00001	0.00001	0.00001	0.00001	0.00001	0.00001	0.00001	0.00001	0.00001	0.00001	0.00001
1000	0.05	0.048	0.0534	0.0542	0.0499	0.0583	0.0467	0.0475	0.0516	0.0525	0.0506	0.0273
	0.01	0.0104	0.0097	0.0105	0.0092	0.0111	0.0098	0.0102	0.0097	0.0091	0.0102	0.005
	0.001	0.001	0.001	0.0011	0.001	0.0011	0.0011	0.001	0.001	0.001	0.001	0.0005
	0.0001	0.00008	0.00009	0.0001	0.00009	0.0001	0.0001	0.0001	0.00009	0.00008	0.0001	0.0001
	0.00001	0.00001	0.00001	0.00001	0.00001	0.00001	0.00001	0.00001	0.00001	0.00001	0.00001	0.00001
2000	0.05	0.0503	0.0489	0.0523	0.0496	0.0671	0.0484	0.0514	0.0522	0.0463	0.0475	0.0257
	0.01	0.01	0.0103	0.0108	0.0096	0.0128	0.0098	0.0107	0.0097	0.0102	0.0097	0.0053
	0.001	0.001	0.001	0.0011	0.001	0.0013	0.001	0.001	0.001	0.001	0.001	0.0005
	0.001	0.00011	0.00011	0.0001	0.00011	0.00011	0.0001	0.0001	0.0001	0.00011	0.0001	0.0001
	0.00001	0.00001	0.00001	0.00001	0.00001	0.00001	0.00001	0.00001	0.00001	0.00001	0.00001	0.00001

Table S11. Type 1 error rates of 11 statistics for testing the association of common and rare variants in a gene with 5 highly correlated traits.

Sample Size	Nominal Level	QRFCCA	FCCA	GAMuT	SCCA	USAT	MANOVA	CCA	PCA	KCCA	MSKAT	minP
	0.05	0.051	0.0497	0.0534	0.0506	0.0407	0.0502	0.0517	0.0534	0.0454	0.0491	0.031
	0.01	0.0105	0.0106	0.0108	0.0107	0.0077	0.0097	0.0106	0.0105	0.0099	0.0097	0.0063
500	0.001	0.001	0.0011	0.001	0.001	0.0009	0.001	0.0009	0.001	0.0011	0.001	0.0005
	0.0001	0.0001	0.0001	0.0001	0.0001	0.0001	0.0001	0.0001	0.00011	0.0001	0.0001	0.00011
	0.00001	0.00001	0.00001	0.00001	0.00001	0.00001	0.00001	0.00001	0.00001	0.00001	0.00001	0.00001
	0.05	0.0524	0.0485	0.0526	0.0455	0.0581	0.0485	0.0479	0.0488	0.0436	0.0498	0.0267
	0.01	0.0101	0.0107	0.011	0.0101	0.0109	0.0103	0.0096	0.0098	0.0104	0.0094	0.0052
1000	0.001	0.0009	0.001	0.0012	0.001	0.0011	0.0009	0.001	0.001	0.0009	0.001	0.0005
	0.0001	0.00009	0.00009	0.0001	0.00008	0.00009	0.00009	0.00009	0.00009	0.00008	0.00009	0.00009
	0.00001	0.00001	0.00001	0.00001	0.00001	0.00001	0.00001	0.00001	0.00001	0.00001	0.00001	0.00001
	0.05	0.0479	0.0484	0.0562	0.0503	0.0661	0.0501	0.053	0.0523	0.0451	0.0483	0.0273
	0.01	0.0098	0.0106	0.0109	0.0104	0.0127	0.01	0.0105	0.01	0.0098	0.0097	0.0056
2000	0.001	0.001	0.0009	0.0012	0.001	0.0014	0.001	0.0009	0.0009	0.0009	0.0011	0.0006
	0.001	0.00011	0.00011	0.0001	0.0001	0.00009	0.0001	0.00009	0.0001	0.00011	0.0001	0.0001
	0.00001	0.00001	0.00001	0.00001	0.00001	0.00001	0.00001	0.00001	0.00001	0.00001	0.00001	0.00001

Table S12. Type 1 error rates of 11 statistics for testing the association of common and rare variants in a gene with 10 low correlated traits.

Sample Size	Nominal Level	QRFCCA	FCCA	GAMuT	SCCA	USAT	MANOVA	CCA	PCA	KCCA	MSKAT	minP
	0.05	0.0479	0.049	0.0488	0.0502	0.0365	0.0489	0.0501	0.0518	0.0475	0.0503	0.0319
	0.01	0.0107	0.0102	0.01	0.0099	0.0078	0.01	0.0099	0.01	0.0093	0.01	0.0056
500	0.001	0.001	0.001	0.001	0.001	0.0008	0.001	0.001	0.001	0.0009	0.0011	0.0007
	0.0001	0.00011	0.0001	0.0001	0.0001	0.0001	0.0001	0.0001	0.0001	0.00011	0.0001	0.0001
	0.00001	0.00001	0.00001	0.00001	0.00001	0.00001	0.00001	0.00001	0.00001	0.00001	0.00001	0.00001
	0.05	0.0486	0.0501	0.0537	0.0471	0.0569	0.0475	0.0496	0.0489	0.047	0.0515	0.0264
	0.01	0.0101	0.01	0.0114	0.0108	0.0118	0.01	0.0094	0.0099	0.0092	0.0103	0.0047
1000	0.001	0.001	0.001	0.0011	0.001	0.0011	0.001	0.001	0.001	0.001	0.001	0.0006
	0.0001	0.0001	0.0001	0.0001	0.0001	0.0001	0.0001	0.0001	0.0001	0.0001	0.0001	0.0001
	0.00001	0.00001	0.00001	0.00001	0.00001	0.00001	0.00001	0.00001	0.00001	0.00001	0.00001	0.00001
	0.05	0.0528	0.0502	0.0539	0.0516	0.0632	0.0511	0.0471	0.0514	0.047	0.0464	0.0292
	0.01	0.0098	0.0105	0.0109	0.0096	0.0127	0.0102	0.0101	0.0109	0.0101	0.0102	0.0052
2000	0.001	0.001	0.001	0.0011	0.0009	0.0013	0.0011	0.001	0.001	0.0009	0.001	0.0005
	0.001	0.00009	0.00009	0.0001	0.00009	0.0001	0.0001	0.0001	0.0001	0.00009	0.0001	0.00011
	0.00001	0.00001	0.00001	0.00001	0.00001	0.00001	0.00001	0.00001	0.00001	0.00001	0.00001	0.00001

Table S13. Type 1 error rates of 11 statistics for testing the association of common and rare variants in a gene with 10 moderately correlated traits.

Sample Size	Nominal Level	QRFCCA	FCCA	GAMuT	SCCA	USAT	MANOVA	CCA	PCA	KCCA	MSKAT	minP
500	0.05	0.0478	0.0524	0.0525	0.0524	0.0399	0.0499	0.052	0.0498	0.0507	0.0536	0.0308
	0.01	0.0104	0.0105	0.0099	0.0097	0.0079	0.0104	0.0098	0.0108	0.0105	0.0106	0.0058
	0.001	0.001	0.001	0.001	0.0009	0.0008	0.001	0.0011	0.001	0.0009	0.001	0.0006
	0.0001	0.00012	0.00011	0.00011	0.00012	0.00011	0.0001	0.00011	0.00011	0.00012	0.0001	0.00011
	0.00001	0.00001	0.00001	0.00001	0.00001	0.00001	0.00001	0.00001	0.00001	0.00001	0.00001	0.00001
1000	0.05	0.05	0.0468	0.052	0.0506	0.0619	0.0501	0.0485	0.0507	0.0425	0.0513	0.0252
	0.01	0.0107	0.0104	0.0116	0.01	0.0119	0.0107	0.0101	0.0095	0.0098	0.0097	0.0051
	0.001	0.001	0.001	0.001	0.001	0.0012	0.001	0.0011	0.001	0.001	0.001	0.0005
	0.0001	0.00009	0.0001	0.0001	0.0001	0.0001	0.0001	0.0001	0.0001	0.0001	0.0001	0.0001
	0.00001	0.00001	0.00001	0.00001	0.00001	0.00001	0.00001	0.00001	0.00001	0.00001	0.00001	0.00001
2000	0.05	0.05	0.0502	0.0557	0.0524	0.061	0.0504	0.0502	0.0477	0.0474	0.0481	0.0253
	0.01	0.0099	0.0098	0.0102	0.0103	0.0129	0.0095	0.0094	0.0109	0.0095	0.0092	0.0056
	0.001	0.001	0.001	0.0012	0.001	0.0013	0.0009	0.001	0.0011	0.001	0.001	0.0005
	0.001	0.00011	0.00011	0.00011	0.00011	0.00011	0.0001	0.0001	0.0001	0.00011	0.0001	0.00009
	0.00001	0.00001	0.00001	0.00001	0.00001	0.00001	0.00001	0.00001	0.00001	0.00001	0.00001	0.00001

Table S14. Type 1 error rates of 11 statistics for testing the association of common and rare variants in a gene with 10 highly correlated traits.

Sample Size	Nominal Level	QRFCCA	FCCA	GAMuT	SCCA	USAT	MANOVA	CCA	PCA	KCCA	MSKAT	minP
500	0.05	0.053	0.0469	0.0508	0.0517	0.0377	0.0504	0.0503	0.0512	0.0455	0.0497	0.0308
	0.01	0.0101	0.0104	0.0101	0.0099	0.0084	0.0094	0.01	0.01	0.0102	0.0105	0.006
	0.001	0.001	0.001	0.0011	0.0009	0.0008	0.001	0.001	0.001	0.001	0.001	0.0006
	0.0001	0.00008	0.00009	0.00008	0.00008	0.00008	0.0001	0.00009	0.00009	0.00008	0.0001	0.0001
	0.00001	0.00001	0.00001	0.00001	0.00001	0.00001	0.00001	0.00001	0.00001	0.00001	0.00001	0.00001
1000	0.05	0.0476	0.048	0.0542	0.0513	0.0532	0.0478	0.0491	0.0493	0.0452	0.0487	0.0257
	0.01	0.0103	0.0102	0.0107	0.0097	0.0121	0.0107	0.0098	0.0103	0.0101	0.0096	0.005
	0.001	0.001	0.001	0.0011	0.0011	0.0011	0.0011	0.001	0.0009	0.0009	0.0011	0.0005
	0.0001	0.0001	0.0001	0.00011	0.0001	0.00011	0.0001	0.0001	0.0001	0.0001	0.0001	0.0001
	0.00001	0.00001	0.00001	0.00001	0.00001	0.00001	0.00001	0.00001	0.00001	0.00001	0.00001	0.00001
2000	0.05	0.0489	0.0515	0.0555	0.047	0.065	0.0464	0.0494	0.0525	0.0484	0.0463	0.0267
	0.01	0.0098	0.0098	0.0109	0.0093	0.0129	0.0093	0.0106	0.0097	0.0092	0.0103	0.005
	0.001	0.001	0.001	0.0011	0.001	0.0013	0.0011	0.001	0.001	0.0009	0.0011	0.0005
	0.001	0.0001	0.00009	0.00009	0.00009	0.0001	0.0001	0.00011	0.00011	0.0001	0.0001	0.00011
	0.00001	0.00001	0.00001	0.00001	0.00001	0.00001	0.00001	0.00001	0.00001	0.00001	0.00001	0.00001

Table S15. Type 1 error rates of 11 statistics for testing the association of common and rare variants in a gene with 15 low correlated traits.

Sample Size	Nominal Level	QRFCCA	FCCA	GAMuT	SCCA	USAT	MANOVA	CCA	PCA	KCCA	MSKAT	minP
	0.05	0.0482	0.0478	0.0493	0.0486	0.0425	0.0527	0.0512	0.0498	0.0472	0.0509	0.0292
	0.01	0.0093	0.0095	0.0103	0.0098	0.0071	0.01	0.0099	0.0101	0.0091	0.0093	0.0062
500	0.001	0.001	0.001	0.001	0.001	0.0008	0.0011	0.001	0.001	0.001	0.001	0.0006
	0.0001	0.0001	0.0001	0.0001	0.0001	0.0001	0.0001	0.0001	0.0001	0.00009	0.0001	0.0001
	0.00001	0.00001	0.00001	0.00001	0.00001	0.00001	0.00001	0.00001	0.00001	0.00001	0.00001	0.00001
	0.05	0.0522	0.0536	0.0543	0.046	0.0578	0.0539	0.0483	0.0507	0.05	0.0525	0.0251
	0.01	0.0097	0.0103	0.0117	0.0102	0.0117	0.0103	0.0098	0.0101	0.0095	0.0102	0.0054
1000	0.001	0.001	0.001	0.0011	0.0009	0.0011	0.001	0.001	0.001	0.001	0.001	0.0005
	0.0001	0.00011	0.00011	0.00011	0.00011	0.0001	0.00011	0.0001	0.00011	0.00011	0.0001	0.00011
	0.00001	0.00001	0.00001	0.00001	0.00001	0.00001	0.00001	0.00001	0.00001	0.00001	0.00001	0.00001
	0.05	0.0487	0.049	0.0515	0.0496	0.0643	0.0523	0.0508	0.0505	0.0487	0.0488	0.0257
	0.01	0.0104	0.0097	0.0101	0.0098	0.013	0.0103	0.0099	0.0093	0.0097	0.0106	0.0055
2000	0.001	0.001	0.0009	0.001	0.001	0.0013	0.001	0.001	0.001	0.0008	0.001	0.0005
	0.001	0.00008	0.00008	0.00008	0.00008	0.00009	0.0001	0.00009	0.00009	0.00008	0.0001	0.0001
	0.00001	0.00001	0.00001	0.00001	0.00001	0.00001	0.00001	0.00001	0.00001	0.00001	0.00001	0.00001

Table S16. Type 1 error rates of 11 statistics for testing the association of common and rare variants in a gene with 15 moderately correlated traits.

Sample Size	Nominal Level	QRFCCA	FCCA	GAMuT	SCCA	USAT	MANOVA	CCA	PCA	KCCA	MSKAT	minP
500	0.05	0.0522	0.0508	0.0501	0.0511	0.0418	0.0523	0.0527	0.0497	0.0488	0.0527	0.0314
	0.01	0.0099	0.01	0.0095	0.01	0.0086	0.0096	0.0098	0.0108	0.0089	0.0099	0.0065
	0.001	0.001	0.001	0.001	0.001	0.0008	0.001	0.001	0.001	0.0009	0.001	0.0006
	0.0001	0.00012	0.00012	0.00012	0.00012	0.00011	0.00011	0.00012	0.00011	0.00012	0.0001	0.00011
	0.00001	0.00001	0.00001	0.00001	0.00001	0.00001	0.00001	0.00001	0.00001	0.00001	0.00001	0.00001
1000	0.05	0.0533	0.0494	0.0535	0.0523	0.0583	0.0506	0.0525	0.0541	0.0474	0.0531	0.0281
	0.01	0.01	0.0098	0.011	0.0106	0.0112	0.0093	0.0103	0.0103	0.0096	0.0098	0.0058
	0.001	0.001	0.001	0.0011	0.001	0.0011	0.0009	0.001	0.001	0.0009	0.001	0.0005
	0.0001	0.0001	0.0001	0.00009	0.0001	0.0001	0.0001	0.00009	0.0001	0.00011	0.0001	0.0001
	0.00001	0.00001	0.00001	0.00001	0.00001	0.00001	0.00001	0.00001	0.00001	0.00001	0.00001	0.00001
2000	0.05	0.0485	0.0485	0.0561	0.0506	0.0633	0.0507	0.0494	0.0489	0.0465	0.0498	0.0279
	0.01	0.0092	0.0101	0.0112	0.0098	0.0135	0.0096	0.0109	0.0097	0.0094	0.0097	0.005
	0.001	0.001	0.0011	0.0011	0.001	0.0012	0.001	0.001	0.001	0.0011	0.001	0.0006
	0.001	0.00011	0.00011	0.00011	0.00011	0.00012	0.00011	0.00011	0.00011	0.00011	0.0001	0.00011
	0.00001	0.00001	0.00001	0.00001	0.00001	0.00001	0.00001	0.00001	0.00001	0.00001	0.00001	0.00001

Table S17. A list of the 46 traits in 13 major phenotype groups.

Blood	Hgb
	MCV
	PCV
	PLT
	RBC
	WBC
	MCH
Homocysteine	Homocysteine
Glycaemia	Glucose
	Insulin
Insulin resistent	HOMA_b
	HOMA_ir
Lipid	TotalCholesterol
	HDL
	LDL
	VLDL
	ApoA1
	ApoB
	Triglycerides
Inflammatory	HsCRP
Kidney	Uric_Acid
	Bicarbonate
	Creatinine
	Phosphate
	Sodium
	Urea
Liver	Albumin
	Alkaline
	Bilirubin
	GGT
Body	Height
	Weight
	BMI
	Waist
	Hip
	WHR
Electrical	ECG HeartRate
	RR Interval

	QT Interval
BP	Systolic BP<br>Diastolic BP
Lung	FVC<br>FEV1<br>FEV1.FVC Ratio
Extended	Total Fat Mass<br>Total Lean Mass

Table S18. A list of P-values of 54 genes significantly associated with 46 traits us

	Statistical Methods						
Gene	QRFCCA	FCCA	GAMuT	SCCA	USAT	MANOVA	CCA
VILL	1.7E-08	2.4E-07	3.0E-06	1.2E-04	9.1E-01	9.8E-01	9.5E-01
KRT27	2.0E-08	3.6E-08	9.4E-02	2.3E-03	9.8E-01	9.7E-01	9.6E-01
RNU6-1243P	2.3E-08	1.3E-07	1.4E-05	1.3E-04	8.1E-01	8.4E-01	9.1E-01
RP11-6N17.10	2.5E-08	5.3E-08	8.5E-06	2.7E-04	8.0E-01	8.4E-01	9.3E-01
AL358134.2	2.8E-08	1.1E-08	1.6E-02	8.1E-04	9.3E-01	9.1E-01	9.9E-01
GAPDH	3.0E-08	6.5E-07	7.3E-04	1.1E-04	9.3E-01	8.1E-01	8.9E-01
DERL1	3.5E-08	1.1E-06	8.0E-04	2.0E-04	9.9E-01	9.3E-01	9.4E-01
RP1-102E24.9	4.3E-08	2.9E-06	3.1E-06	1.5E-04	8.6E-01	9.5E-01	9.7E-01
PPBP	4.8E-08	2.8E-08	9.2E-06	7.0E-04	9.8E-01	9.4E-01	9.6E-01
LINC00443	5.1E-08	6.1E-05	5.4E-06	2.3E-04	9.6E-01	9.6E-01	9.3E-01
RP11-10O17.1	5.2E-08	1.2E-04	1.4E-01	2.7E-04	9.7E-01	9.5E-01	9.5E-01
PROCA1	5.6E-08	4.2E-07	9.8E-04	5.9E-03	7.1E-01	6.7E-01	9.6E-01
CHMP2B	6.0E-08	3.3E-08	5.1E-06	3.0E-04	8.2E-01	9.3E-01	9.2E-01
IGKV2-4	6.3E-08	2.8E-07	5.9E-06	1.3E-04	9.9E-01	8.1E-01	9.1E-01
AC017104.1	8.2E-08	1.2E-06	1.4E-03	2.2E-03	9.2E-01	8.3E-01	9.5E-01
RNU6-980P	8.2E-08	2.7E-07	5.0E-04	3.2E-04	9.7E-01	7.0E-01	8.3E-01
AC016691.2	8.9E-08	8.3E-07	5.5E-03	1.9E-04	9.3E-01	9.2E-01	9.9E-01
RNA5SP19	9.0E-08	2.4E-07	1.1E-03	6.8E-04	9.9E-01	6.2E-01	9.6E-01
snoU13	9.2E-08	9.8E-05	9.9E-06	1.3E-04	9.0E-01	9.1E-01	9.4E-01
MLN	9.5E-08	6.6E-07	4.7E-06	1.1E-04	9.0E-01	9.4E-01	9.5E-01
RP11-55L4.2	1.0E-07	8.9E-07	1.1E-03	6.3E-03	8.2E-01	3.6E-01	7.0E-01
CTC-505O3.2	1.2E-07	4.0E-07	9.9E-03	2.3E-03	9.5E-01	9.3E-01	9.6E-01
ISCA1P1	1.2E-07	2.2E-07	1.3E-03	1.3E-04	9.9E-01	9.4E-01	9.1E-01
JUN	1.3E-07	8.7E-08	4.6E-02	1.0E-06	7.9E-01	9.9E-01	9.9E-01
AC140061.12	1.4E-07	1.8E-07	8.0E-03	1.2E-04	8.3E-01	1.2E-02	1.1E-01
RP11-697E2.10	1.8E-07	2.7E-07	5.4E-03	4.0E-04	6.2E-01	6.1E-01	8.5E-01
RNU6-717P	1.9E-07	2.5E-07	9.1E-03	1.7E-03	9.8E-01	2.9E-01	5.8E-01
RP11-98D18.17	2.4E-07	2.5E-07	1.1E-03	6.6E-03	9.2E-01	3.8E-01	6.8E-01
PPIAP13	2.4E-07	2.3E-06	3.6E-02	2.4E-04	9.6E-01	9.5E-01	9.1E-01
SETP2	2.7E-07	2.6E-06	2.7E-04	6.0E-04	8.0E-01	9.7E-01	9.4E-01
AC008691.1	3.2E-07	7.4E-07	3.4E-04	1.1E-04	9.1E-01	9.2E-01	9.9E-01
RP1-40E16.2	3.4E-07	5.7E-06	1.3E-04	1.8E-04	9.1E-01	7.9E-01	9.8E-01
RP11-79P5.9	3.5E-07	2.5E-07	1.6E-03	7.9E-04	9.9E-01	7.6E-01	9.7E-01
RN7SL699P	3.7E-07	1.6E-06	2.3E-02	8.0E-03	6.6E-01	9.3E-01	9.3E-01
OR4A44P	3.9E-07	3.8E-05	4.9E-03	1.3E-03	8.4E-01	9.8E-01	9.3E-01
C7orf71	4.8E-07	1.2E-07	1.6E-04	7.2E-04	1.0E+00	8.9E-01	9.8E-01
COMP	4.8E-07	1.4E-06	1.3E-03	1.0E-03	8.8E-01	6.7E-01	9.8E-01
PCDHB10	5.0E-07	1.1E-06	5.2E-01	1.8E-04	9.8E-01	8.5E-01	8.8E-01
HOXB1	5.3E-07	7.8E-07	3.9E-03	7.1E-04	9.1E-01	9.0E-01	9.8E-01
RN7SKP280	5.4E-07	5.8E-07	5.2E-03	4.2E-03	9.2E-01	9.6E-01	9.7E-01
SEC31A	5.5E-07	2.7E-05	3.5E-04	2.3E-03	9.7E-01	9.1E-01	9.3E-01

RP11-363E6.4	6.6E-07	8.3E-07	3.6E-02	1.0E-06	9.0E-01	9.4E-01	9.8E-01
SHISA4	6.8E-07	4.0E-07	1.0E-02	3.0E-03	9.8E-01	5.7E-01	9.4E-01
DAZAP2P1	8.0E-07	3.2E-05	6.5E-06	1.5E-04	9.2E-01	1.2E-02	3.0E-02
PGLYRP3	9.0E-07	2.5E-07	4.8E-03	1.3E-04	9.1E-01	9.1E-01	9.5E-01
RP11-138H8.4	1.1E-06	1.9E-05	2.0E-03	1.4E-04	9.2E-01	6.3E-01	9.2E-01
RP11-153M7.3	1.1E-06	2.5E-04	7.0E-03	6.0E-04	8.7E-01	9.6E-01	1.0E+00
EPS8L2	1.2E-06	1.7E-06	5.5E-02	1.6E-03	9.6E-01	9.7E-01	9.7E-01
HPN	1.2E-06	4.7E-06	2.0E-02	1.3E-04	7.2E-01	9.6E-01	9.3E-01
AC092685.1	1.3E-06	4.0E-04	6.5E-06	1.1E-04	9.8E-01	9.0E-01	9.2E-01
AC079781.1	1.4E-06	2.5E-04	2.2E-04	1.4E-03	8.8E-01	5.7E-02	1.5E-01
CTD-2014B16.2	1.4E-06	7.1E-06	9.8E-06	1.3E-03	9.5E-01	9.6E-01	9.8E-01
SULT1B1	1.4E-06	1.7E-05	1.6E-03	1.5E-03	7.0E-01	9.8E-01	9.1E-01
RP11-57G22.1	1.4E-06	3.7E-05	8.2E-03	1.6E-03	8.1E-01	2.4E-01	6.6E-01

sing QRFCCA

PCA	KCCA	MSKAT
7.0E-05	9.0E-01	1.32E-05
1.2E-01	9.3E-01	0.000331
8.2E-05	6.9E-01	8.87E-09
5.8E-08	3.2E-01	6.68E-10
5.0E-01	9.0E-01	0.015107
8.7E-07	1.0E+00	5.56E-06
2.4E-02	9.6E-01	0.027771
7.8E-06	8.1E-01	7.64E-06
3.7E-04	6.7E-01	1.67E-06
7.5E-03	4.3E-01	0.002061
7.7E-02	2.2E-01	0.089856
7.4E-02	8.3E-01	1.14E-06
5.5E-02	4.6E-01	4.04E-06
4.4E-04	1.0E+00	0.000662
2.7E-04	1.0E+00	2.77E-05
1.1E-07	4.1E-01	0.000294
1.3E-07	6.5E-01	0.002929
3.0E-06	9.4E-01	1.30E-08
9.9E-01	5.6E-01	5.76E-06
2.6E-04	9.4E-01	5.77E-10
3.6E-01	9.6E-03	0.000578
4.6E-02	2.4E-01	9.57E-08
3.9E-06	5.3E-01	0.018924
7.8E-01	2.6E-01	0.000111
1.1E-02	3.6E-01	6.43E-06
9.9E-01	1.1E-02	0.000469
5.6E-01	2.5E-06	0.006229
2.9E-07	8.6E-03	0.009645
8.2E-02	9.0E-01	7.86E-07
5.6E-04	3.7E-01	0.045012
4.0E-03	4.6E-01	6.88E-06
7.6E-02	9.2E-01	7.73E-05
2.8E-01	7.5E-01	0.000329
7.5E-01	4.8E-01	6.88E-05
1.8E-02	2.2E-01	0.002266
6.3E-02	3.3E-01	0.037812
5.0E-02	2.2E-01	7.54E-07
7.3E-01	3.7E-01	0.001004
2.1E-03	5.1E-01	0.240587
4.4E-02	9.2E-01	8.68E-07
1.6E-06	3.1E-01	0.060277

2.7E-01	6.3E-01	0.008889
5.2E-01	1.0E+00	0.002748
5.4E-06	2.7E-06	0.009093
2.3E-01	5.7E-01	2.34E-06
1.2E-01	3.7E-01	2.86E-05
7.1E-02	9.5E-01	0.000218
3.4E-01	4.3E-01	0.003988
4.7E-01	3.1E-01	0.000156
4.4E-03	1.0E+00	0.001274
6.4E-08	7.8E-03	7.85E-05
6.4E-02	5.4E-01	1.48E-07
9.0E-02	9.4E-01	0.000431
2.1E-03	3.9E-01	0.009357

Table S19. A list of top ten genes ranked by GAMuT.

Gene	Chr	QRFCCA	GAMuT
AC008694.3	5	1.39E-08	2.43E-06
RP4-742C19.12	22	1.04E-05	2.78E-06
VILL	3	1.69E-08	2.96E-06
RP1-102E24.9	12	4.32E-08	3.06E-06
RNU6-1229P	7	5.83E-03	3.41E-06
TNNT3	11	4.94E-06	3.52E-06
MLN	6	9.47E-08	4.70E-06
CHMP2B	3	6.03E-08	5.06E-06
LINC00443	13	5.08E-08	5.37E-06
RP11-400N9.1	2	2.73E-05	5.41E-06

Table S20. A list of P-value of all rare SNPs within gene ADAM19.

	P-value
QFCCA (ADAM19)	6.07E-11
GAMuT (ADAM19)	8.17E-01
SNP or Genomic Position	
rs191008415	4.05E-28
156968065	3.18E-25
156910533	1.57E-08
156990160	3.51E-08
rs35911462	2.08E-06
156932396	3.20E-06
rs149236204	1.93E-04
rs146419018	4.76E-04
rs147457580	7.28E-04
156997304	8.48E-04
rs139626715	6.10E-03
rs201250158	8.31E-03
rs143506109	3.10E-02
rs188856674	3.77E-02
rs149634622	4.06E-02
rs188436117	4.18E-02
rs34603646	4.95E-02
156954511	5.82E-02
rs78468979	6.33E-02
rs184699772	7.06E-02
156944190	7.81E-02
rs11466758	8.18E-02
rs62388506	8.18E-02
rs11465266	9.26E-02
rs141181941	1.03E-01
rs188195103	1.15E-01
156990480	1.24E-01
rs184288975	1.33E-01
rs141821577	1.37E-01
rs115527546	1.43E-01
rs62388487	1.47E-01
rs115502782	1.49E-01
rs139340220	1.62E-01
rs4704869	1.73E-01
rs114882516	1.88E-01
rs56243836	1.97E-01
rs116556518	2.10E-01
rs7725400	2.13E-01
rs11744541	2.23E-01
rs72811356	2.24E-01
rs55717305	2.38E-01
rs11466803	2.41E-01

rs151138638	2.52E-01
rs184623076	2.55E-01
rs185647117	2.56E-01
rs143031163	2.65E-01
rs72811314	2.66E-01
156933734	2.92E-01
rs186859909	3.05E-01
rs181070111	3.05E-01
rs6556092	3.09E-01
rs79116280	3.09E-01
rs144608237	3.14E-01
rs181426847	3.15E-01
rs138725997	3.68E-01
rs184205806	4.02E-01
rs34882961	4.12E-01
rs2902557	4.27E-01
rs183481901	4.45E-01
rs62388508	4.69E-01
rs115525841	5.03E-01
rs72811306	5.13E-01
rs146616158	5.22E-01
rs147490600	5.27E-01
rs141699542	5.30E-01
rs10078120	5.51E-01
rs150874473	5.56E-01
rs114799047	5.66E-01
156944672	5.79E-01
156898877	5.99E-01
156937135	6.04E-01
rs116298982	6.13E-01
rs148264060	6.26E-01
rs142509617	6.72E-01
rs77760678	6.87E-01
rs200159796	6.88E-01
rs114064162	7.06E-01
rs78564361	7.06E-01
rs72811340	7.07E-01
156959046	7.10E-01
rs187328397	7.19E-01
rs144038216	7.55E-01
rs191259967	7.57E-01
rs112724886	7.59E-01
rs182111619	7.60E-01
rs11465245	7.65E-01
rs114912544	7.67E-01
rs149216255	7.76E-01
rs34750645	7.82E-01

rs11465265	7.89E-01
156927814	7.94E-01
rs115231378	8.01E-01
rs62388526	8.02E-01
rs56384823	8.10E-01
rs11466808	8.13E-01
rs149445339	8.15E-01
rs183178230	8.22E-01
156989890	8.24E-01
rs11465278	8.30E-01
rs112307138	8.31E-01
rs11466814	8.35E-01
rs181287300	8.65E-01
rs115133320	8.68E-01
rs146558727	8.72E-01
rs185494681	8.76E-01
rs138324979	8.83E-01
rs189254582	8.84E-01
rs147064466	8.88E-01
156971132	8.94E-01
156995485	9.09E-01
rs113511170	9.18E-01
rs189607262	9.35E-01
rs11465257	9.49E-01
rs61753548	9.49E-01
rs114990221	9.50E-01
rs181394073	9.60E-01
rs141189988	9.61E-01
rs111644980	9.67E-01
rs150670035	9.77E-01
rs143341739	9.77E-01
rs11466811	9.79E-01
rs72811335	9.79E-01
rs78606727	9.82E-01
rs188402615	9.86E-01
rs11466767	9.95E-01
rs146539692	9.98E-01
rs75821300	9.98E-01
rs115271681	9.99E-01
156958272	9.99E-01

Table S21. A list of traits which a single gene was associated with.

Gene	Chormosome				
CTC-498M16.2	5	ALL	5.74E-22	HOMA_ir	6.56E-26
TRAJ22	14	ALL	2.16E-20	HOMA_ir	3.56E-28
AP000351.10	22	ALL	2.09E-18	HOMA_ir	4.68E-25
HAR1B	20	ALL	7.81E-18	Creatinine	1.23E-51
IGHVII-20-1	14	ALL	7.49E-16	Creatinine	2.01E-29
RP11-4F5.2	15	ALL	9.94E-16	HOMA_ir	2.03E-20
RNVU1-17	1	ALL	3.90E-13	Total_Fat_Ma	0.000718852
PNOC	8	ALL	1.63E-12	VLDL	9.15E-09
COTL1P1	17	ALL	8.71E-12	HOMA_ir	7.30E-09
LINC00273	16	ALL	4.41E-11	Creatinine	8.57E-13
snoU13	12	ALL	4.95E-11	HOMA_ir	9.84E-15
ADAM19	5	ALL	6.07E-11	HOMA_b	1.02E-31
CTD-2026G6.2	3	ALL	1.91E-10	HOMA_b	2.57E-22
MIR409	14	ALL	2.77E-10	Creatinine	7.40E-19
RP1-276E15.1	11	ALL	3.13E-10	Creatinine	3.23E-09
HMGN1P6	2	ALL	4.46E-10	HsCRP	0.000126436
HOXA7	7	ALL	2.18E-09	Creatinine	1.41E-24
RNA5SP99	2	ALL	2.41E-09	Creatinine	6.03E-12
AC021660.1	3	ALL	2.69E-09	Hip	0.001745979
RP11-561N12.1	7	ALL	3.12E-09	HOMA_ir	1.80E-13
FBXL5	4	ALL	4.53E-09	Creatinine	6.40E-05
PPIAP23	13	ALL	5.40E-09	Glucose	8.37E-10
HOXB2	17	ALL	7.00E-09	HOMA_b	0.000564076
RP11-170N16.1	4	ALL	7.09E-09	HOMA_ir	4.97E-05
AC008694.3	5	ALL	1.39E-08	Creatinine	0.003954873
VILL	3	ALL	1.69E-08	Creatinine	2.23E-08
KRT27	17	ALL	2.04E-08	HOMA_ir	1.40E-05
RNU6-1243P	11	ALL	2.28E-08	Creatinine	2.75E-05
RP11-6N17.10	17	ALL	2.49E-08	HOMA_ir	1.95E-07
AL358134.2	6	ALL	2.83E-08	Creatinine	1.33E-21
GAPDH	12	ALL	2.96E-08	Creatinine	3.45E-08
DERL1	8	ALL	3.45E-08	Creatinine	5.04E-06
RP1-102E24.9	12	ALL	4.32E-08	Creatinine	5.21E-07
PPBP	4	ALL	4.79E-08	Creatinine	0.000282027
LINC00443	13	ALL	5.08E-08	Creatinine	1.66E-11
RP11-10O17.1	15	ALL	5.19E-08	Glucose	2.90E-08
PROCA1	17	ALL	5.62E-08	HOMA_ir	8.28E-07
CHMP2B	3	ALL	6.03E-08	Creatinine	0.002346817
IGKV2-4	2	ALL	6.33E-08	Creatinine	0.000421234
AC017104.1	2	ALL	8.15E-08	HOMA_ir	8.77E-11
RNU6-980P	19	ALL	8.16E-08	HOMA_ir	1.30E-12
AC016691.2	2	ALL	8.89E-08	Creatinine	1.12E-08

RNA5SP19	1	ALL	9.03E-08	HOMA_ir	9.48E-09
snoU13	6	ALL	9.16E-08	HsCRP	0.000380108
MLN	6	ALL	9.47E-08	Glucose	6.24E-11
RP11-55L4.2	17	ALL	1.03E-07	HOMA_ir	2.18E-11
CTC-505O3.2	5	ALL	1.22E-07	Creatinine	1.37E-06
ISCA1P1	5	ALL	1.23E-07	VLDL	1.41E-07
JUN	1	ALL	1.25E-07	Creatinine	3.44E-20
AC140061.12	12	ALL	1.41E-07	Creatinine	2.11E-16
RP11-697E2.10	15	ALL	1.77E-07	Creatinine	2.22E-21
RNU6-717P	12	ALL	1.91E-07	HsCRP	2.50E-12
RP11-98D18.17	1	ALL	2.36E-07	HOMA_ir	2.77E-06
PPIAP13	10	ALL	2.43E-07	Creatinine	1.90E-06
SETP2	14	ALL	2.67E-07	Creatinine	1.15E-22
AC008691.1	5	ALL	3.15E-07	Creatinine	2.56E-07
RP1-40E16.2	6	ALL	3.38E-07	HsCRP	6.49E-10
RP11-79P5.9	5	ALL	3.54E-07	Glucose	3.99E-07
RN7SL699P	17	ALL	3.69E-07	HOMA_ir	2.86E-09
OR4A44P	11	ALL	3.94E-07	GGT	0.005636472
C7orf71	7	ALL	4.80E-07	PLT	0.000205223
COMP	19	ALL	4.83E-07	Creatinine	9.58E-15
PCDHB10	5	ALL	4.97E-07	HsCRP	3.02E-11
HOXB1	17	ALL	5.27E-07	HOMA_ir	7.99E-07
RN7SKP280	7	ALL	5.39E-07	Alkaline	0.000294435
SEC31A	4	ALL	5.51E-07	Uric_Acid	0.000657855
RP11-363E6.4	8	ALL	6.56E-07	PLT	3.81E-12
SHISA4	1	ALL	6.84E-07	HOMA_ir	1.48E-08
DAZAP2P1	2	ALL	7.97E-07	Creatinine	0.000290958
PGLYRP3	1	ALL	8.97E-07	Creatinine	8.97E-10
RP11-138H8.4	15	ALL	1.09E-06	Bilirubin	5.60E-05
RP11-153M7.3	4	ALL	1.10E-06	PCV	0.01321897
EPS8L2	11	ALL	1.15E-06	Creatinine	2.49E-08
HPN	19	ALL	1.15E-06	Creatinine	9.61E-07
AC092685.1	7	ALL	1.34E-06	Creatinine	4.49E-08
AC079781.1	7	ALL	1.36E-06	HsCRP	1.50E-10
CTD-2014B16.2	14	ALL	1.41E-06	Phosphate	0.000388972
SULT1B1	4	ALL	1.43E-06	TotalCholeste	0.000987772
RP11-57G22.1	18	ALL	1.44E-06	Diastolic_BP	0.000361512

Insulin	4.26E-11	Glucose	3.84E-08	Systolic_I	0.001956	Diastolic_	0.029416	
Glucose	7.53E-12	Insulin	2.47E-11	Urea	0.006493	Uric_Acic	0.048675	
Insulin	6.69E-10	Glucose	2.13E-07	Phosphate	0.013555	Height	0.022553	ApoA1
Urea	2.17E-07	GGT	0.008421	WHR	0.011319	Waist	0.019045	Albumin
Urea	6.33E-05	WBC	0.010865	ECG_Hea	0.01918	RR_Interv	0.02229	TotalChol
Insulin	5.26E-10	Homocyst	2.56E-06	Glucose	0.001579	HsCRP	0.026557	Urea
Hip	0.000771	BMI	0.001168	Weight	0.001773	HOMA_ir	0.002227	Insulin
Triglyceride	9.15E-09	HOMA_ir	1.21E-07	Insulin	0.002612	Glucose	0.00826	FEV1
Glucose	2.41E-08	GGT	5.35E-07	Insulin	0.006809			
Glucose	1.15E-05	HOMA_ir	3.35E-05	LDL	0.001232	TotalChol	0.007672	Height
Insulin	1.70E-06	ApoB	0.001961	MCH	0.003053	Glucose	0.010992	
Creatinine	1.16E-05	Glucose	0.002678	HsCRP	0.004913	Insulin	0.029635	Triglyceri
GGT	6.17E-07	Bicarbona	0.016391					
Hip	7.19E-07	Total_Lea	2.15E-06	Weight	1.65E-05	BMI	0.000148	Urea
HOMA_ir	7.14E-08	Insulin	1.40E-05	HOMA_b	0.0011	Homocyst	0.001438	Waist
HOMA_b	0.001411	HOMA_ir	0.001467	Glucose	0.041752			
Urea	8.97E-06	HOMA_b	0.017358	Bilirubin	0.01787	Total_Lea	0.034116	PCV
Diastolic_E	0.007365	HsCRP	0.019323	Insulin	0.019335	Bicarbona	0.020459	Total_Lea
GGT	0.002209	HOMA_b	0.005389	Systolic_I	0.012018	BMI	0.012338	Creatinine
Glucose	7.41E-08	Insulin	5.07E-07	Sodium	1.85E-05	ApoB	0.001165	HsCRP
PCV	0.002071	Hgb	0.002115	Phosphate	0.00811	Alkaline	0.022042	RBC
Creatinine	2.48E-06	HOMA_ir	4.31E-06	Insulin	0.001131	FVC	0.002255	FEV1.FV
Homocyste	0.001419	Hgb	0.002353	PCV	0.004896	Sodium	0.008226	QT_Interv
Insulin	0.01038	Total_Fat_	0.01354	HsCRP	0.016426	FEV1.FV	0.022457	Homocyst
ECG_Heart	0.005142	RR_Interv	0.006365	Sodium	0.022489	Bilirubin	0.023863	Alkaline
TotalChole	0.001057	LDL	0.002498	ApoB	0.002941	HsCRP	0.006357	Waist
VLDL	0.000197	Triglyceri	0.000197	HsCRP	0.000407	Creatinine	0.000792	Bicarbona
Glucose	9.44E-05	GGT	0.002032	FEV1.FV	0.003483	Total_Lea	0.00548	WBC
Creatinine	2.93E-06	Insulin	3.27E-05	Diastolic_	0.000887	Glucose	0.002072	QT_Interv
Urea	0.033409	FVC	0.046864					
HOMA_ir	1.43E-05	VLDL	4.62E-05	Triglyceri	4.62E-05	Glucose	0.000164	HsCRP
Urea	0.000999	Alkaline	0.001949	Uric_Acic	0.003094	Glucose	0.005175	HOMA_ir
GGT	0.001544	Uric_Acic	0.00814	HsCRP	0.008199	MCV	0.017594	PLT
HOMA_ir	0.001707	GGT	0.006442	MCH	0.03834	MCV	0.039668	HsCRP
Urea	0.006732	Diastolic_	0.025616	HOMA_b	0.045981	Total_Lea	0.047506	WBC
HOMA_ir	6.99E-08	Creatinine	5.23E-06	Bicarbona	5.23E-05	Insulin	0.000102	Sodium
Insulin	0.000451	Phosphate	0.002443	Urea	0.00394	Alkaline	0.00543	Height
ApoA1	0.006587	Albumin	0.014431	TotalChol	0.015285	HOMA_ir	0.015898	Alkaline
ApoB	0.002251	Insulin	0.016273	Glucose	0.022997	TotalChol	0.034153	HOMA_b
Triglyceride	3.00E-05	VLDL	3.00E-05	GGT	7.96E-05	Insulin	9.39E-05	Diastolic_
Insulin	1.61E-06	Glucose	0.000767	Hip	0.020059	FVC	0.028685	GGT
HOMA_ir	6.26E-07	HOMA_b	2.07E-06	Insulin	1.34E-05	RBC	0.013824	Weight

Insulin	0.000458	FEV1.FV	0.003888	Homocyst	0.014141	WBC	0.02851	
Uric_Acid	0.03257							
RR_Interva	5.47E-05	ECG_Hea	9.24E-05	HOMA_ir	0.001933	MCV	0.004181	QT_Interv
Insulin	2.81E-05	Glucose	0.011116	Albumin	0.033516	FEV1	0.040244	
HsCRP	7.38E-06	PLT	0.000999	HOMA_ir	0.007889	Alkaline	0.024527	GGT
Triglyceride	1.41E-07	GGT	0.000152	FEV1	0.001886	Hgb	0.012808	PCV
HsCRP	7.82E-05	Urea	0.002911	MCH	0.008826	Weight	0.028407	MCV
Urea	9.78E-06	Sodium	0.035051	FVC	0.047822			
Urea	0.001289	GGT	0.013236	Diastolic_	0.029205	Sodium	0.038107	
Urea	0.019226	Albumin	0.03704	WHR	0.037243			
HsCRP	3.35E-06	Homocyst	0.001038	FVC	0.0016	Insulin	0.001639	Total_Lea
HsCRP	1.96E-05	GGT	2.04E-05	ApoB	0.030931	Height	0.033145	
Urea	0.00034	Bilirubin	0.036947	Height	0.040664			
GGT	6.12E-05	HOMA_ir	0.00272	Waist	0.005602	WHR	0.00708	HOMA_b
HOMA_b	0.019383							
HOMA_ir	2.49E-06	ECG_Hea	0.003014	RR_Interv	0.003645	QT_Interv	0.022067	Weight
Triglyceride	2.68E-05	VLDL	2.68E-05	Insulin	0.000281	Homocyst	0.00589	Glucose
Glucose	0.006122							
MCV	0.002319	PCV	0.003021	Creatinine	0.003439	HOMA_ir	0.004737	MCH
Albumin	0.005359	VLDL	0.007411	Triglyceri	0.007411	HOMA_b	0.042892	
Homocyste	9.79E-10	Diastolic_	0.000348	Systolic_I	0.006508			
Insulin	1.83E-05	VLDL	0.000498	Triglyceri	0.000498	Total_Lea	0.003318	Weight
Urea	0.002344	HDL	0.003536	Bilirubin	0.004372	Creatinine	0.005524	Bicarbona
PCV	0.002636	Albumin	0.016639	Hgb	0.023312	RBC	0.026391	
HOMA_b	3.53E-05							
Insulin	0.000242	ApoA1	0.000997	Glucose	0.009315	Sodium	0.020391	
HOMA_b	0.002674	Bilirubin	0.038279					
HOMA_ir	1.38E-05	Insulin	0.003741	HDL	0.018741	QT_Interv	0.024385	Systolic_I
Creatinine	0.00034	Systolic_I	0.000721	PLT	0.028488	HOMA_ir	0.030747	
VLDL	0.038599	Triglyceri	0.038599					
HsCRP	0.00138	Waist	0.007796	Homocyst	0.013655	TotalChol	0.01674	LDL
Urea	0.000528	Glucose	0.006229	HOMA_ir	0.011997	Diastolic_	0.016194	HsCRP
HOMA_ir	6.15E-05	Insulin	0.006397	PLT	0.008098	Glucose	0.019831	RBC
Creatinine	0.000345	Bilirubin	0.017111	Urea	0.040224			
MCV	0.000412	MCH	0.000563	RBC	0.002557	HsCRP	0.006245	HOMA_b
MCV	0.00747	MCH	0.007912	Sodium	0.008705	HOMA_ir	0.011954	Hgb
Sodium	0.001378	HOMA_b	0.016447	FEV1	0.034765	Albumin	0.037049	Systolic_I

0.02525	ECG_Hea	0.03049	RR_Interv	0.04365					
0.03739									
0.03686	QT_Interv	0.03774	Systolic_I	0.03906	Sodium	0.03999			
0.03519	FEV1	0.03717	HOMA_b	0.03738					
0.0171	Waist	0.01799	Total_Lea	0.03105	RBC	0.04576			
0.01372	Albumin	0.02241	FEV1.FV	0.03082					
0.01615	ApoB	0.01743	Total_Lea	0.01918	Hgb	0.02495	Phosphate	0.03771	WHR
0.03395	VLDL	0.03395							
0.00026	Waist	0.00035	Uric_Acic	0.00206	Total_Fat_	0.0024	HsCRP	0.03814	Insulin
0.00256	RBC	0.00636	Urea	0.01393	WHR	0.01409	BMI	0.01537	Total_Lea
0.03621									
0.03352	Glucose	0.03451	Triglyceri	0.0478	VLDL	0.0478			
0.01659	Diastolic_	0.01743	Bicarbona	0.02732	Sodium	0.03585	Height	0.03943	HOMA_i
0.00434	FEV1.FV	0.00536	BMI	0.00849	VLDL	0.00979	Triglyceri	0.00979	Albumin
0.0243									
0.00483	FEV1	0.00681	Alkaline	0.01493	Height	0.01873	PLT	0.03135	Phosphate
0.00984	ECG_Hea	0.01326	Insulin	0.01521	RR_Interv	0.01787	FEV1.FV	0.03069	Diastolic_
0.0271	HOMA_b	0.03586	Urea	0.04716					
0.02777	MCH	0.03854	MCV	0.04543	HsCRP	0.04685			
0.0083	BMI	0.02207	HOMA_i	0.02618	Bicarbona	0.04052			
0.00328	PLT	0.01206	Insulin	0.01342	Hgb	0.03425	QT_Interv	0.04934	
0.00642	HOMA_b	0.00842	ApoA1	0.01216	BMI	0.01447	Weight	0.03267	Diastolic_
0.0092	ECG_Hea	0.00959	Systolic_I	0.01572	RR_Interv	0.01974	Weight	0.02842	HDL
0.00113	Systolic_I	0.03504	Insulin	0.0444					
0.00884	HOMA_b	0.00953	WBC	0.01846	Insulin	0.02524	VLDL	0.04373	Triglyceri
0.02181	FEV1.FV	0.02501	Urea	0.03353	Insulin	0.04318	MCH	0.04625	
0.04319									
0.04829	GGT	0.04881	QT_Interv	0.04924	FEV1	0.04952			
0.00039	Homocyst	0.02875							
0.00649	Uric_Acic	0.00811	Systolic_I	0.01451	Creatinine	0.0187	GGT	0.04389	
0.02332	Bilirubin	0.02373	Hip	0.03508	Phosphate	0.03719			
0.0356									
0.00166	Glucose	0.00856	Height	0.03327	Total_Lea	0.04348			
0.02979	Sodium	0.04044	FEV1	0.04109					
0.01919	Hip	0.02757	BMI	0.03119	Systolic_I	0.03384	MCV	0.04497	

0.00782	RBC	0.01174	MCH	0.01522	Bilirubin	0.02582	BMI	0.02987	HsCRP
0.03343									
0.01294	FVC	0.01431	WHR	0.02436	Bilirubin	0.03443			
0.03015									
0.00216	FEV1	0.01157	BMI	0.01347	Weight	0.02145	Systolic_I	0.04599	Total_Fat_
0.01947	Total_Lea	0.02162	Insulin	0.02283	Total_Fat_	0.02878	Weight	0.02913	BMI
0.02791	VLDL	0.0369	Triglyceri	0.0369	Total_Lea	0.04158	Insulin	0.04671	Total_Fat_
0.00997	Urea	0.03917							
0.00488	RBC	0.00767	WBC	0.01534	Hgb	0.02067	RR_Interv	0.02213	Urea
0.01617	Glucose	0.02728							
0.00636	Total_Fat_	0.01651	RBC	0.01796	BMI	0.02298	Waist	0.03608	LDL
0.03971									
0.02185	Height	0.04661							
0.01971									
0.03197									
0.00745	WHR	0.01033	WBC	0.01243	Systolic_I	0.0132	Bicarbona	0.03434	QT_Interv
0.01576	QT_Interv	0.02616	GGT	0.03635	Bilirubin	0.04596	LDL	0.04968	
0.04199	FEV1.FV	0.0441							

0.04524									
0.04015									
0.02513	WBC	0.029847	Weight	0.030837					
0.0475									
0.01049	Total_Fat_	0.014248	LDL	0.017979	PLT	0.024192	TotalChole	0.025487	Alkaline
0.03718	ApoA1	0.039995	GGT	0.040511					
0.03209	GGT	0.03463	HOMA_ir	0.035082	RBC	0.043982	HsCRP	0.047932	
0.03995	Albumin	0.041229							
0.02898	Height	0.029157	Total_Lear	0.031394	Waist	0.036981	BMI	0.040466	Hip
0.04373									

0.03668	Total_Lear	0.03782	Insulin	0.039445	Phosphate	0.042606
0.04808						
0.04033	Height	0.045741				
0.04797	Homocyste	0.048268				
0.03522	Triglycerid	0.043163	VLDL	0.043163		
0.03663	Hip	0.037032				
0.04624						

0.040268

0.041994 Urea 0.042429

Table S22. Number of genes consisting of only rare variants significantly associated with single trai

Blood							Homocysteine
Hgb	MCV	PCV	PLT	RBC	WBC	MCH	Homocysteine
0	0	0	3	2	0	0	10
**Lipid**							**Inflammatory**
TotalChol	HDL	LDL	VLDL	ApoA1	ApoB	Triglycerides	HsCRP
0	0	0	11	0	0	11	72
**Kidney**							**Liver**
Uric Acid	Bicarbona	Creatinine	Phosphate	Sodium	Urea	Albumin	Alkaline
0	1	345	0	0	3	0	0
**Body**							**Electrical**
Height	Weight	BMI	Waist	Hip	WHR	ECG Heart Rate	RR Interval
1	0	0	0	1	0	0	0

t.

Glycaemia		Insulin Resistance		
Glucose	Insulin	HOMA-B	HOMA-IR	
21	15	20	108	
Extended				
Total Fat I	Total Lean Mass			
0	0			
		Lung		
Bilirubin	GGT	FVC	FEV1	FEV1-FVC-Ratio
2	14	2	2	0
	BP			
QT Interva	SBP	DBP		
0	0	1		

Table S23. A list of P-values of top 42 genes with common variants only significantly associated w

	Statistical Methods						
Gene	QRFCCA	FCCA	GAMuT	SCCA	USAT	MANOVA	CCA
UBE2U	2.9E-15	4.0E-15	2.6E-02	1.2E-04	8.3E-01	9.0E-01	9.3E-01
BATF2	5.9E-15	2.3E-11	6.9E-01	3.2E-03	8.0E-02	9.3E-01	9.6E-01
C5orf51	1.0E-14	7.1E-15	1.0E-03	2.0E-06	4.9E-01	9.8E-01	9.6E-01
RP11-814P5.1	1.3E-14	3.6E-15	7.2E-01	4.5E-04	6.0E-01	1.0E+00	9.7E-01
USP44	3.5E-14	1.3E-14	2.7E-01	1.5E-03	8.2E-01	9.9E-01	9.1E-01
C12orf5	4.9E-14	5.2E-14	4.7E-01	1.0E-03	6.9E-01	9.8E-01	9.3E-01
SSH2	5.6E-14	3.0E-14	7.4E-03	3.3E-05	8.4E-01	9.9E-01	9.0E-01
PHIP	3.0E-13	2.5E-13	4.2E-01	1.7E-05	2.5E-01	9.8E-01	9.2E-01
ARRDC4	1.7E-12	1.8E-12	7.7E-01	6.9E-04	3.8E-01	9.7E-01	9.9E-01
RP3-477O4.16	4.3E-12	9.7E-12	9.3E-01	2.8E-02	5.2E-01	1.0E+00	1.0E+00
FAM210B	5.6E-11	2.4E-11	2.5E-03	1.6E-05	3.9E-01	9.4E-01	9.6E-01
AF001550.7	6.5E-11	3.3E-11	5.8E-02	1.5E-04	5.9E-01	9.3E-01	1.0E+00
COLGALT1	9.5E-11	9.1E-11	9.9E-01	7.5E-04	8.1E-01	9.9E-01	9.1E-01
IGSF5	1.2E-10	1.3E-06	1.2E-01	1.1E-03	9.2E-01	9.1E-01	9.3E-01
GPR137B	3.5E-10	1.8E-10	3.1E-01	4.8E-04	5.4E-01	9.7E-01	9.4E-01
RCL1	4.9E-10	5.3E-09	7.1E-02	1.0E-06	6.1E-01	9.8E-01	9.6E-01
OXR1	1.8E-09	3.7E-09	4.0E-03	1.0E-06	9.4E-01	9.9E-01	9.9E-01
CTD-2503O16.4	1.5E-08	5.7E-09	1.7E-01	1.4E-04	9.8E-01	9.1E-01	9.7E-01
C10orf76	1.9E-08	9.1E-08	2.7E-01	3.0E-05	1.6E-01	9.0E-01	9.6E-01
SRRM4	2.3E-08	1.4E-06	1.0E-01	1.2E-04	4.7E-01	9.8E-01	9.3E-01
RASAL3	5.3E-08	3.6E-07	7.0E-01	1.9E-04	1.6E-01	8.7E-01	9.9E-01
MTHFD2P6	5.7E-08	8.3E-08	3.7E-05	1.6E-05	4.0E-01	1.5E-03	1.1E-02
UQCRBP3	7.6E-08	7.5E-08	5.4E-01	2.3E-03	5.0E-01	9.3E-01	1.0E+00
SEC14L3	7.9E-08	8.2E-07	2.4E-02	1.5E-05	5.1E-01	9.6E-01	9.4E-01
UGCG	8.0E-08	1.1E-07	5.5E-01	3.0E-03	1.4E-01	9.4E-01	9.7E-01
ZNF680	1.2E-07	4.0E-05	3.1E-01	3.8E-04	3.2E-01	9.1E-01	9.3E-01
GPR56	1.3E-07	3.8E-07	6.1E-01	6.1E-05	3.6E-01	9.8E-01	9.5E-01
CCDC26	2.0E-07	2.1E-07	1.4E-01	1.0E-06	1.1E-01	9.1E-01	9.3E-01
SOX5	2.5E-07	1.2E-05	8.6E-01	1.0E-06	4.9E-01	9.0E-01	9.1E-01
AC009892.10	3.2E-07	4.3E-07	9.8E-01	2.4E-02	3.6E-01	4.0E-01	6.6E-01
HPSE	3.2E-07	3.2E-05	4.7E-01	1.0E-06	3.3E-01	9.7E-01	9.4E-01
SLC35E4	5.1E-07	1.5E-05	3.9E-01	3.8E-04	7.1E-01	9.5E-01	9.1E-01
CTC-575D19.1	6.3E-07	2.7E-06	6.5E-01	6.8E-04	7.6E-01	1.0E+00	1.0E+00
EFTUD1	7.9E-07	6.2E-07	2.9E-02	9.0E-06	3.8E-01	9.7E-01	9.4E-01
RP4-742N3.1	9.4E-07	4.1E-06	1.9E-01	1.1E-04	7.3E-01	9.1E-01	9.3E-01
RP11-568J23.7	9.7E-07	5.5E-04	1.0E-02	1.5E-03	8.1E-01	9.8E-01	1.0E+00
AC138655.1	1.0E-06	7.7E-06	6.7E-01	7.0E-06	6.3E-01	9.7E-01	1.0E+00
EYA4	1.0E-06	1.1E-04	9.0E-01	2.1E-04	2.4E-01	9.4E-01	9.6E-01
P2RY6	1.1E-06	1.4E-06	9.2E-01	7.0E-04	8.7E-01	9.5E-01	9.5E-01
BACH2	1.2E-06	1.8E-06	6.0E-02	5.0E-06	6.7E-01	9.6E-01	9.4E-01
MSH6	1.4E-06	6.8E-06	7.2E-01	1.0E-06	9.7E-01	9.4E-01	9.9E-01

CTC-497E21.5	1.5E-06	4.8E-06	3.3E-01	1.0E-06	4.3E-01	9.5E-01	9.6E-01

ith 46 traits using QRFCCA.

PCA	KCCA	MSKAT
1.1E-01	9.0E-01	0.647115
4.2E-01	9.5E-01	0.012373
6.4E-01	9.7E-01	8.23E-05
3.5E-01	9.6E-01	0.461213
5.2E-01	9.9E-01	0.00138
5.5E-01	9.2E-01	0.0199
2.2E-01	9.7E-01	0.185851
7.1E-01	9.2E-01	0.042022
8.6E-01	9.7E-01	0.337233
8.8E-01	9.5E-01	0.861207
5.3E-02	9.4E-01	0.524432
9.1E-02	9.7E-01	0.139853
5.7E-02	9.6E-01	0.041859
1.7E-01	9.0E-01	0.547865
5.0E-01	9.9E-01	0.446398
5.9E-01	9.2E-01	0.908544
6.7E-01	9.6E-01	0.102838
1.7E-01	9.8E-01	0.298597
1.2E-01	9.4E-01	0.167248
3.2E-01	9.1E-01	0.117424
3.4E-01	9.1E-01	0.023521
2.7E-02	9.7E-07	2.05E-05
1.5E-01	1.0E+00	0.260786
1.1E-03	9.9E-01	0.184885
8.8E-01	9.0E-01	0.009934
7.9E-02	9.2E-01	0.001049
8.5E-01	9.3E-01	0.08076
1.1E-01	9.7E-01	2.69E-05
7.9E-01	9.3E-01	0.13182
9.2E-01	1.0E+00	0.986532
8.0E-01	1.0E+00	0.173608
5.6E-01	9.6E-01	0.00046
1.1E-01	9.4E-01	0.018417
1.5E-01	9.5E-01	0.153352
3.4E-02	9.5E-01	0.213592
1.6E-01	9.9E-01	9.47E-05
1.7E-04	9.5E-01	0.315623
6.5E-01	9.5E-01	0.000302
7.5E-01	9.6E-01	0.340408
5.0E-01	9.5E-01	0.458542
4.2E-01	9.6E-01	0.792843

3.7E-01	9.9E-01	0.078548

Table S24. A list of traits which a single gene was associated with.

Gene	Chormosome				
REG1B	2	ALL	1.65E-116	HOMA-IR	5.02E-94
RP11-665C14.1	4	ALL	1.38E-93	Creatinine	1.39E-187
ZNF160	19	ALL	1.98E-91	HOMA-IR	2.34E-80
LEF1	4	ALL	7.44E-83	HsCRP	1.22E-112
DYNC1H1	14	ALL	3.46E-58	HOMA-IR	4.76E-108
DOCK7	1	ALL	4.42E-51	HsCRP	3.66E-53
SHC3	9	ALL	7.56E-42	HOMA-IR	1.90E-49
Y_RNA	7	ALL	1.89E-36	HsCRP	1.04E-71
CTD-2122P11.1	5	ALL	1.62E-33	HsCRP	3.86E-45
GBF1	10	ALL	6.30E-28	HOMA-B	2.91E-17
RP1-8B22.1	1	ALL	1.75E-27	HOMA-B	2.03E-26
VPS13D	1	ALL	2.62E-26	Creatinine	1.41E-17
RP11-68I3.2	17	ALL	3.20E-24	Creatinine	1.04E-46
SLC13A3	20	ALL	8.51E-24	HsCRP	2.03E-13
RP11-167N24.3	12	ALL	4.33E-23	HOMA-B	6.43E-18
UBA6	4	ALL	5.26E-22	Creatinine	1.04E-42
GAN	16	ALL	1.49E-21	HsCRP	1.04E-15
RP4-794H19.2	1	ALL	4.07E-21	HOMA-IR	4.85E-19
RP11-142I20.1	18	ALL	5.29E-21	HsCRP	3.22E-18
METAP2	12	ALL	2.62E-20	HOMA-B	3.14E-32
SLCO1C1	12	ALL	5.89E-20	HOMA-B	4.25E-08
AC105443.2	7	ALL	2.47E-17	HsCRP	1.01E-46
GRN	17	ALL	1.63E-16	HOMA-IR	2.28E-29
INTS12	4	ALL	1.72E-16	HsCRP	3.85E-27
RP11-323I15.5	15	ALL	9.29E-16	Homocysteine	1.42E-07
UBE2U	1	ALL	2.88E-15	PLT	6.44E-16
BATF2	11	ALL	5.90E-15	HsCRP	4.68E-47
C5orf51	5	ALL	1.02E-14	Glucose	2.30E-18
RP11-814P5.1	15	ALL	1.28E-14	Creatinine	1.66E-32
USP44	12	ALL	3.49E-14	HsCRP	1.81E-31
C12orf5	12	ALL	4.91E-14	Creatinine	1.16E-15
SSH2	17	ALL	5.59E-14	RR_Interval	6.94E-07
PHIP	6	ALL	2.96E-13	HsCRP	7.78E-34
ARRDC4	15	ALL	1.73E-12	Creatinine	3.09E-10
RP3-477O4.16	20	ALL	4.33E-12	Creatinine	3.23E-32
FAM210B	20	ALL	5.59E-11	Triglycerides	2.99E-11
AF001550.7	16	ALL	6.53E-11	HsCRP	3.47E-15
COLGALT1	19	ALL	9.53E-11	HOMA-B	1.43E-18
IGSF5	21	ALL	1.24E-10	Triglycerides	5.02E-25
GPR137B	1	ALL	3.54E-10	GGT	6.49E-09
RCL1	9	ALL	4.88E-10	Creatinine	6.46E-09
OXR1	8	ALL	1.77E-09	Creatinine	2.27E-05

CTD-2503O16.4	5	ALL	1.48E-08	HsCRP	2.40E-05
C10orf76	10	ALL	1.89E-08	HOMA-IR	1.03E-12
SRRM4	12	ALL	2.26E-08	HsCRP	4.14E-06
RASAL3	19	ALL	5.31E-08	Creatinine	4.60E-07
MTHFD2P6	5	ALL	5.65E-08	Total_Fat_Mas	3.09E-06
UQCRBP3	5	ALL	7.58E-08	Glucose	5.54E-06
SEC14L3	22	ALL	7.90E-08	Bilirubin	0.000531772
UGCG	9	ALL	7.98E-08	HOMA-IR	1.70E-08
ZNF680	7	ALL	1.23E-07	HOMA-B	2.43E-10
GPR56	16	ALL	1.26E-07	Total_Fat_Mas	0.000154365
CCDC26	8	ALL	1.97E-07	Diastolic_BP	0.000755246
SOX5	12	ALL	2.54E-07	Creatinine	0.001588142
AC009892.10	19	ALL	3.17E-07	HOMA-IR	1.82E-11
HPSE	4	ALL	3.21E-07	FEV1.FVC_Ra	5.85E-06
SLC35E4	22	ALL	5.08E-07	HOMA-IR	8.74E-28
CTC-575D19.1	5	ALL	6.25E-07	ApoB	0.000153801
EFTUD1	15	ALL	7.87E-07	QT_Interval	0.000109083
RP4-742N3.1	7	ALL	9.42E-07	HOMA-IR	1.06E-10
RP11-568J23.7	16	ALL	9.69E-07	Creatinine	2.46E-12
AC138655.1	2	ALL	1.01E-06	Total_Fat_Mas	2.24E-06
EYA4	6	ALL	1.05E-06	HDL	0.000701759
P2RY6	11	ALL	1.11E-06	HsCRP	9.58E-22
BACH2	6	ALL	1.24E-06	Height	0.001786206
MSH6	2	ALL	1.42E-06	Homocysteine	4.24E-06
CTC-497E21.5	11	ALL	1.49E-06	HOMA-IR	1.54E-06

Insulin	1.81E-24	VLDL	2.72E-23	Triglycerides	2.72E-23
Urea	4.04E-13	HOMA-IR	6.55E-10	Insulin	1.23E-06
Glucose	6.20E-46	Insulin	3.02E-18	HsCRP	1.44E-15
HOMA-B	7.17E-52	Homocysteine	2.24E-14	Sodium	0.000119
Insulin	2.83E-28	Glucose	1.07E-10	Creatinine	2.57E-09
HOMA-B	6.42E-13	FEV1.FVC_Ratio	9.00E-07	BMI	9.36E-07
Creatinine	5.69E-14	Insulin	1.66E-13	Glucose	2.43E-06
Creatinine	0.000461	BMI	0.002677	Homocysteine	0.002837
HOMA-B	3.77E-09	Waist	2.55E-07	Hip	4.46E-06
Weight	1.37E-05	Total_Lean_Mass	6.16E-05	Waist	7.70E-05
Creatinine	4.49E-13	TotalCholesterol	0.000584	LDL	0.001085
VLDL	6.07E-12	Triglycerides	6.07E-12	HOMA-B	1.32E-09
HOMA-B	7.06E-10	Urea	0.002398	Insulin	0.002598
GGT	3.12E-08	HOMA-B	1.21E-07	VLDL	4.85E-06
GGT	3.89E-12	Glucose	2.68E-05	Hgb	0.006287
HOMA-B	3.76E-14	BMI	4.16E-06	Urea	1.07E-05
HOMA-B	3.79E-13	Glucose	8.21E-06	FEV1.FVC_Ratio	0.000691
Creatinine	3.18E-06	Insulin	2.56E-05	HOMA-B	4.27E-05
HOMA-B	1.11E-05	WHR	0.001233	VLDL	0.008252
HOMA-IR	3.73E-17	Insulin	8.61E-10	Glucose	2.41E-05
HOMA-IR	9.80E-07	Glucose	7.22E-05	Insulin	0.00016
HOMA-B	3.19E-07	WHR	0.000333	Insulin	0.00095
Insulin	7.89E-10	Creatinine	1.52E-05	Bicarbonate	0.000866
Creatinine	4.57E-07	Weight	0.000154	Total_Fat_Mass	0.000261
PLT	7.61E-07	MCH	9.69E-05	MCV	0.000147
Bilirubin	3.55E-05	HOMA-B	0.001161	LDL	0.001454
HDL	0.043139	Phosphate	0.044932		
Total_Fat_Mass	3.75E-05	Hip	0.000112	BMI	0.000167
HOMA-IR	0.000354	Weight	0.005078	Total_Lean_Mass	0.005568
HOMA-IR	7.75E-11	PLT	5.99E-05	Glucose	0.004112
GGT	1.41E-06	HOMA-IR	2.80E-06	Homocysteine	0.000104
ECG_HeartRate	5.29E-06	QT_Interval	0.001606	Bilirubin	0.002129
HOMA-B	3.87E-10	Phosphate	0.015091	Weight	0.037039
HsCRP	1.12E-06	Glucose	1.31E-06	FEV1.FVC_Ratio	7.38E-05
Urea	0.004779	Triglycerides	0.005126	VLDL	0.005126
VLDL	2.99E-11	Bilirubin	9.32E-07	HOMA-IR	1.25E-06
Hip	0.005354	Bilirubin	0.015295	Glucose	0.027995
HOMA-IR	0.000917	Diastolic_BP	0.00571	Glucose	0.009317
VLDL	5.02E-25	Alkaline	0.002263	PLT	0.00899
HOMA-B	1.06E-07	MCV	0.00048	Height	0.000577
Glucose	0.004099	ApoB	0.004803	HOMA-IR	0.029542
Glucose	0.000568	Homocysteine	0.002654	Urea	0.004073

PLT	0.000443	VLDL	0.022382	Triglycerides	0.022382
Glucose	2.11E-11	HsCRP	3.57E-09	Insulin	0.000125
Creatinine	0.00041	Hgb	0.00344	Alkaline	0.004603
HsCRP	1.25E-05	Phosphate	0.006127	TotalCholesterol	0.013511
Hip	7.59E-06	Weight	8.23E-06	BMI	1.34E-05
Alkaline	0.000196	Hip	0.001602	Weight	0.004886
ApoA1	0.000565	WBC	0.000922	GGT	0.014172
Insulin	0.003289	Weight	0.004129	Total_Lean_Mass	0.005566
HsCRP	0.001447	Hip	0.001457	Weight	0.001727
Phosphate	0.002479	Albumin	0.004063	HOMA-IR	0.007726
WBC	0.008621	Homocysteine	0.010815	PCV	0.011788
Insulin	7.18E-05	Glucose	0.021864		
RBC	1.85E-05	PCV	0.000236	FEV1	0.001912
Insulin	2.33E-08	Glucose	0.000238	RR_Interval	0.001502
HOMA-B	0.000771	HDL	0.001531	Albumin	0.001641
PCV	0.001651	Bilirubin	0.003559	Weight	0.011849
Insulin	0.000664	Glucose	0.001042	PCV	0.013281
Bicarbonate	0.000883	GGT	0.001962	VLDL	0.004607
Waist	3.95E-06	Hip	6.58E-06	Weight	2.10E-05
ApoA1	0.00644	Alkaline	0.007152	ApoB	0.008848
Homocysteine	5.46E-06	WBC	0.000756	Systolic_BP	0.02079
Systolic_BP	0.00394	Creatinine	0.008234	Albumin	0.019751
PLT	0.001577	HOMA-B	0.002078	Uric_Acid	0.008233
Uric_Acid	0.001223	Insulin	0.001659	Glucose	0.034376

PLT	2.09E-22	Glucose	1.31E-21	Urea	0.001561
Glucose	1.70E-05	WHR	0.000901	Hgb	0.008246
GGT	1.59E-07	Alkaline	0.000339	ApoB	0.000795
RR_Interval	0.0054	FEV1.FVC_Ratio	0.01839	ECG_HeartRate	0.033583
RBC	0.002369				
Weight	1.23E-05	Hip	0.000329	Total_Fat_Mass	0.000794
PCV	9.33E-05	QT_Interval	0.000714	Sodium	0.000903
Weight	0.004683	Urea	0.005013	Hip	0.015885
Weight	4.52E-06	Total_Fat_Mass	9.47E-06	BMI	1.70E-05
BMI	7.76E-05	Hip	0.00019	MCH	0.000218
MCV	0.003455	MCH	0.004398	ApoB	0.008071
HOMA-IR	1.02E-06	Urea	0.001802	Albumin	0.005415
Triglycerides	0.004864	VLDL	0.004864	Albumin	0.041153
Triglycerides	4.85E-06	Glucose	0.000181	FEV1.FVC_Ratio	0.000203
ApoA1	0.008889	PLT	0.010418	Uric_Acid	0.010497
Weight	0.000153	PCV	0.000156	Hip	0.000186
PLT	0.00521	RR_Interval	0.00715	VLDL	0.037518
Triglycerides	4.29E-05	VLDL	4.29E-05	HDL	0.000786
Triglycerides	0.008252	Sodium	0.013139	RR_Interval	0.0485
Height	0.008252	Uric_Acid	0.013208	WHR	0.014709
FVC	0.008165	Hgb	0.009453	Albumin	0.02424
BMI	0.005179	Total_Lean_Mass	0.008607	ECG_HeartRate	0.010927
Waist	0.002229	Uric_Acid	0.003206	HDL	0.003898
BMI	0.000288	Diastolic_BP	0.000306	FVC	0.001513
HOMA-B	0.000856	Diastolic_BP	0.010965	RR_Interval	0.022709
ApoB	0.002595	WBC	0.002639	ApoA1	0.007485
Weight	0.000228	Waist	0.000846	VLDL	0.002308
Urea	0.017938	BMI	0.018468	RBC	0.021578
Insulin	0.007591				
Height	0.010275	Urea	0.015338	Insulin	0.034351
Height	0.022761	ApoA1	0.027094	GGT	0.030202
Sodium	0.000159				
Insulin	3.73E-05	RR_Interval	0.000234	Total_Lean_Mass	0.00054
Total_Lean_Mass	0.030428	GGT	0.031343	Weight	0.032576
Height	0.045353	LDL	0.047224		
PCV	0.011564	Hgb	0.033835	RBC	0.035129
MCH	0.00064	Phosphate	0.04973		
Urea	0.048241				
Systolic_BP	0.014734	GGT	0.035515	WBC	0.048575

Total_Fat_Mass	0.035956	Phosphate	0.040017		
RBC	0.00609	Albumin	0.016666	HDL	0.038753
PCV	0.02823	ECG_HeartRate	0.038164	WHR	0.042638
Bicarbonate	0.035498	Total_Lean_Mass	0.044415		
Waist	2.70E-05	Glucose	0.000789	Total_Lean_Mass	0.000936
BMI	0.005105	Total_Lean_Mass	0.006675		
WHR	0.029127	Waist	0.038068		
BMI	0.007046	PCV	0.007442	Urea	0.012348
BMI	0.002234	Waist	0.003188	HDL	0.00408
HOMA-IR	0.012135	Height	0.041186		
Hgb	0.002284	Triglycerides	0.005669	VLDL	0.005669
ECG_HeartRate	0.004691				
ApoA1	0.002871	Sodium	0.003639	Waist	0.010095
BMI	0.012308	MCV	0.013736	Creatinine	0.016137
Diastolic_BP	0.028085	Triglycerides	0.031963	VLDL	0.031963
Triglycerides	0.004607	Diastolic_BP	0.012267		
BMI	3.32E-05	Insulin	0.000104	HOMA-B	0.000198
ECG_HeartRate	0.018078	Systolic_BP	0.020054	Homocysteine	0.03018
Sodium	0.032962	HOMA-B	0.048787		
Urea	0.010551	WHR	0.021964		

Bilirubin	0.003339	ApoA1	0.01104	Bicarbonate	0.02593
Triglycerides	0.01328	VLDL	0.01328	Bilirubin	0.0156
WBC	0.000861	Uric_Acid	0.0019	Urea	0.00247
Waist	0.001016	MCV	0.00719	WHR	0.00821
Homocysteine	0.000927	Total_Fat_	0.00097	Hgb	0.00147
Total_Fat_Mass	0.028278	Uric_Acid	0.04458	Waist	0.04702
Glucose	5.16E-05	Bilirubin	0.00038	Albumin	0.00074
Total_Fat_Mass	0.000324	RR_Interv	0.00129	MCV	0.00244
PCV	0.039889	Urea	0.04213		
Weight	0.005525	Uric_Acid	0.00573	Total_Lean_Mass	0.00836
Height	0.043719				
Homocysteine	0.002334	WBC	0.00803	RR_Interval	0.00816
Height	0.010503	FEV1.FV(	0.03703	Alkaline	0.03774
Hgb	0.000299	Total_Lea	0.00056	Waist	0.00136
Triglycerides	0.037518	ECG_Hea	0.04026	QT_Interval	0.04668
Glucose	0.006554	MCV	0.03515	ApoA1	0.04226
Urea	0.040728				
PCV	0.030418	WHR	0.03142	FEV1	0.03518
Urea	0.013039	Weight	0.02942	Waist	0.04994
Triglycerides	0.016871	VLDL	0.01687	WBC	0.02665
Waist	0.005047	Systolic_E	0.00506	Insulin	0.00983
ECG_HeartRate	0.023678	QT_Interv	0.03694	WHR	0.04544
TotalCholesterol	0.00909	Albumin	0.01882		
Triglycerides	0.002308	Creatinine	0.00518	Total_Lean_Mass	0.02466
HsCRP	0.035597	Hip	0.03774	Total_Fat_Mass	0.03868
RR_Interval	0.045788	Total_Lea	0.04725		
Weight	0.035397	Phosphate	0.04154		
Hip	0.001597	Weight	0.00163	ECG_HeartRate	0.00205
Phosphate	0.036744	PLT	0.04983		
Diastolic_BP	0.048804				

PLT	0.001363	Creatinine	0.01492	Triglycerides	0.03008
Total_Fat_Mass	0.015874	Hgb	0.03062	Hip	0.03753
PLT	0.007771	Bilirubin	0.00968	Urea	0.01288
Sodium	0.035945	BMI	0.04015	HOMA-IR	0.0414
Insulin	0.012658	QT_Interv	0.01341	Phosphate	0.02754
Total_Lean_Mass	0.030202	Total_Fat_	0.03398	RBC	0.04251
Hgb	0.039197	Albumin	0.04169		
Uric_Acid	0.000326	Total_Lea	0.00247	Diastolic_BP	0.0074
Diastolic_BP	0.038143	WBC	0.04066		

Alkaline	0.03522					
Bicarbonate	0.03188	Homocysteine	0.03202			
MCH	0.01115	Bilirubin	0.01316	WHR	0.01544	MCV
Total_Lean_Mass	0.00937	ApoB	0.01441	Bilirubin	0.0146	MCH
Weight	0.00157	BMI	0.00258	RBC	0.00321	WBC
Total_Lean_Mass	0.00107	FEV1.FVC_Ratio	0.00341	GGT	0.0044	WBC
QT_Interval	0.00366	Hgb	0.00458	LDL	0.01044	HDL
BMI	0.01097	Bilirubin	0.03248	LDL	0.03263	Hip
QT_Interval	0.01406	HDL	0.01604	ApoA1	0.02886	Sodium
Total_Lean_Mass	0.03806	PCV	0.04273	Bicarbona	0.04298	HDL
RBC	0.00139	Total_Fat_Mass	0.00661	Insulin	0.00785	Bilirubin
Weight	0.04664					
Glucose	0.02759	Hip	0.0309	Diastolic_	0.03633	Albumin
Homocysteine	0.01109	FEV1	0.01287	Albumin	0.01523	WBC
Systolic_BP	0.04897					
Bilirubin	0.04091					
Bicarbonate	0.04538					
BMI	0.0023	Glucose	0.0296			

VLDL	0.03008	Insulin	0.03262	RBC	0.03332	WBC
WBC	0.04381					
ApoA1	0.01469	Homocysteine	0.02003	Creatinine	0.03036	
WBC	0.03174	ECG_HeartRate	0.0337	Glucose	0.03528	FEV1.FV[illegible]
Hgb	0.04311	Glucose	0.0448			
Glucose	0.04032	HsCRP	0.04368	Creatinine	0.04573	

0.02861	Waist	0.03393						
0.03141	Urea	0.04543						
0.00396	PLT	0.00577	Hip	0.00778	Total_Lea	0.04461		
0.03371								
0.02101	ECG_Hea	0.03429	HsCRP	0.04106	WHR	0.04756		
0.04276	FVC	0.04688						
0.03993	ECG_Hea	0.04519	Insulin	0.04895				
0.04587								
0.01278	PLT	0.02546	ApoA1	0.04789				
0.0425								
0.01906	GGT	0.02066	Height	0.02288	Hip	0.03367	Uric_Acid	0.03734

0.03762

0.04553	LDL	0.0478	Uric_Acid	0.04891

Table S25. Number of genes consisting of only common variants significantly associated with si

Blood							Homocysteine
Hgb	MCV	PCV	PLT	RBC	WBC	MCH	Homocysteine
0	1	0	4	0	0	1	2
Lipid							Inflammatory
TotalChol	HDL	LDL	VLDL	ApoA1	ApoB	Triglyceri	HsCRP
0	0	0	6	0	1	6	29
Kidney							Liver
Uric Acid	Bicarbona	Creatinine	Phosphate	Sodium	Urea	Albumin	Alkaline
0	0	34	0	0	1	0	0
Body							Electrical
Height	Weight	BMI	Waist	Hip	WHR	ECG Hear	RR Interval
0	0	1	3	0	0	0	1

ingle trait.

Glycaemia		Insulin Resistance		
Glucose	Insulin	HOMA-B	HOMA-IR	
9	8	23	23	

Extended				
Total Fat I	Total Lean Mass			
1	1			

		Lung		
Bilirubin	GGT	FVC	FEV1	FEV1-FVC-Ratio
3	7	0	0	1

	BP			
QT Interva	SBP	DBP		
0	0	0		
